# RSK1-driven TRIM28/E2F1 feedback loop promotes castration-resistant prostate cancer progression

**DOI:** 10.1172/JCI185119

**Published:** 2025-06-16

**Authors:** Miyeong Kim, Jinpeng Liu, Yanquan Zhang, Ruixin Wang, Ryan Goettl, Jennifer Grasso, Derek B. Allison, Chi Wang, Tianyan Gao, Xiaoqi Liu, Ka-Wing Fong

**Affiliations:** 1 Department of Toxicology and Cancer Biology,; 2 Department of Biostatistics, College of Public Health,; 3 Department of Pathology & Laboratory Medicine,; 4 Department of Molecular and Cellular Biochemistry, and; 5 Markey Cancer Center, University of Kentucky, Lexington, Kentucky, USA.

**Keywords:** Cell biology, Endocrinology, Prostate cancer

## Abstract

Castration-resistant prostate cancer (CRPC) marks the advanced and lethal stage of prostate cancer (PCa). TRIM28, also known as KAP1, is a transcriptional regulator recently shown to promote CRPC cell proliferation and xenograft tumor growth. Nonetheless, knowledge gaps persist regarding the mechanisms underlying TRIM28 upregulation in CRPC as well as the genomic targets regulated by TRIM28. Here, we report that *TRIM28* is a E2F1 target in CRPC. Using an integrated genomic approach, we have demonstrated that TRIM28 forms a positive feedback loop to promote the transcriptional activation and genomic function of E2F1 independent of retinoblastoma (Rb) status. Furthermore, we identified RSK1 as a kinase that directly phosphorylates TRIM28 at S473, and, as such, RSK1 drives the TRIM28/E2F1 feedback loop. Accordingly, pS473-TRIM28 promotes CRPC progression, which is mitigated by RSK inhibition. In summary, our study reveals a critical role of the RSK1–TRIM28–E2F1 axis in CRPC progression, which may be exploited as a vulnerability in treating Rb-deficient CRPC.

## Abstract 2

Our research reveals how the RSK1-TRIM28-E2F1 pathway drives advanced prostate cancer, presenting a potential new treatment target.

## Graphical abstract

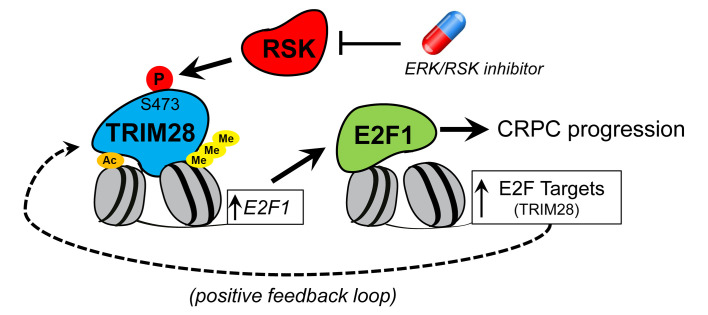

## Introduction

Prostate cancer (PCa) continues to be a major health concern for American men, as it has the highest nonskin cancer incidence (with 299,010 new cases estimated in 2024) and the second-highest cancer mortality (35,250 deaths in 2024). Castration-resistant prostate cancer (CRPC), which develops over a period of months to years, metastasizes to different organs and represents the lethal stage of the disease.

E2Fs are a group of transcription factors (TFs) that control progression through the cell cycle by regulating the transcription of DNA synthesis and cell cycle-related genes (1, 2). The E2F family is characterized by a signature winged-helix DNA binding domain and has been traditionally divided into activator (E2F1–E2F3) and repressor (E2F4–E2F8) subclasses (3). E2F1 is elevated in CRPC (4, 5), which induces PCa cell growth and survival (6, 7). Mechanistically, E2F1 promotes CRPC growth via upregulating androgen receptor (AR) gene expression and AR transcription activity (8). In addition, several studies have demonstrated that E2F1 activates a lineage-specific transcription program to confer drug resistance in CRPC (9–11). Tumor suppressor retinoblastoma protein (Rb) represses the transcription activity of activator E2Fs (E2F1–E2F3) by forming an Rb-E2F repressor complex. In response to mitogenic stimuli, cyclin-dependent kinase (CDK)/cyclin complexes hyper-phosphorylate Rb, which results in Rb disassociation from E2Fs, thus allowing the aforementioned E2F-dependent oncogenic activity (12). CDK4/6 inhibitors, which restore the Rb binding to E2F are currently in widespread clinical use. However, in approximately 10%–15% of CRPC tumors, loss of *RB1* not only unleashes E2F activity but also renders these tumors unamenable to CDK4/6 inhibitors (13–15). As direct pharmacologic inhibition of E2F1 is not currently feasible, it is of great interest to identify the upstream mechanism that promotes E2F1 transcription activation, as this may be exploited as a treatment vulnerability in Rb-deficient CRPC.

Tripartite motif containing 28 (TRIM28), also known as KAP1, was identified as an interacting protein for Krüppel-associated box zinc finger protein more than two decades ago (16–18). Its canonical function is to scaffold epigenetic repressive machineries to promote chromatin condensation and transcription repression of gene expression in viruses such as endogenous retroviruses and HIV (19–21). Despite the longstanding transcription repressor function, multiple studies have implicated TRIM28 site-specific phosphorylation (pS473 and pS824) as a functional switch to convert TRIM28 into a transcription activator in different cellular contexts (20). For instance, TRIM28 has been shown to be phosphorylated by DNA-PK at S824 to regulate the pause release of RNA polymerase II, thereby promoting HIV-1 activation (22, 23). In another study, pS473-TRIM28 was shown to interact with TF MyoD, thus enabling MyoD-dependent transcriptional activation of target gene expression required for ultimate myoblast differentiation (24). We previously reported that *TRIM28* KD attenuates CRPC cell growth (25), which partially resulted from TRIM28 stabilizing oncogenic TF TRIM24 from SPOP-mediated proteasome degradation (25). However, knowledge gaps remain regarding how TRIM28 level is elevated in CRPC and whether TRIM28 site–specific phosphorylation is involved in transcriptional activation of its inadequately defined genomic targets.

The p90 ribosomal S6 kinase (RSK) family of serine/threonine kinases, which comprises four closely related proteins (RSK1–4), constitutes the effector that lies downstream of the Ras/Raf/MEK/ERK signaling pathway (26). RSKs are characterized by a functional N-terminal kinase domain and a C-terminal kinase domain providing a docking site for ERK1/2 phosphorylation at Thr573 and subsequent kinase activation. RSKs preferentially phosphorylate target substrates containing a consensus motif RXRXXpS (26). Aberrant expression of RSKs has been seen in samples from patients with PCa (27, 28). RSK signaling regulates PCa proliferation, cell cycle, cell motility, and therapy resistance via phosphorylation of a wide spectrum of substrates (26). Also, it has been reported that the oncogenic function of RSK1 in PCa is attributed to the activation of an RSK-dependent transcriptional program, which is exemplified by the ability of RSK1/2 to directly phosphorylate and regulate TF CREB and c-Fos, hence initiating a cascade of the downstream signaling pathway (28, 29).

Here, we report that *TRIM28* is an E2F1 target in CRPC. Using an integrative genomic analysis, we revealed that TRIM28 forms a positive feedback loop to promote the transcriptional activation and genomic function of E2F1 regardless of AR and Rb status. Furthermore, this positive feedback loop is driven by RSK1-mediated TRIM28 S473 phosphorylation. As a result, RSK inhibition not only attenuated pS473-TRIM28 but also abolished E2F1-driven CRPC cell proliferation in vitro and Rb-deficient xenograft tumor growth in vivo. Taken together, our data suggest that the RSK1–TRIM28–E2F1 axis may be exploited as a vulnerability in treating Rb-deficient CRPC.

## Results

### TRIM28 is a E2F1 target in advanced prostate cancer.

**Figure 1:**
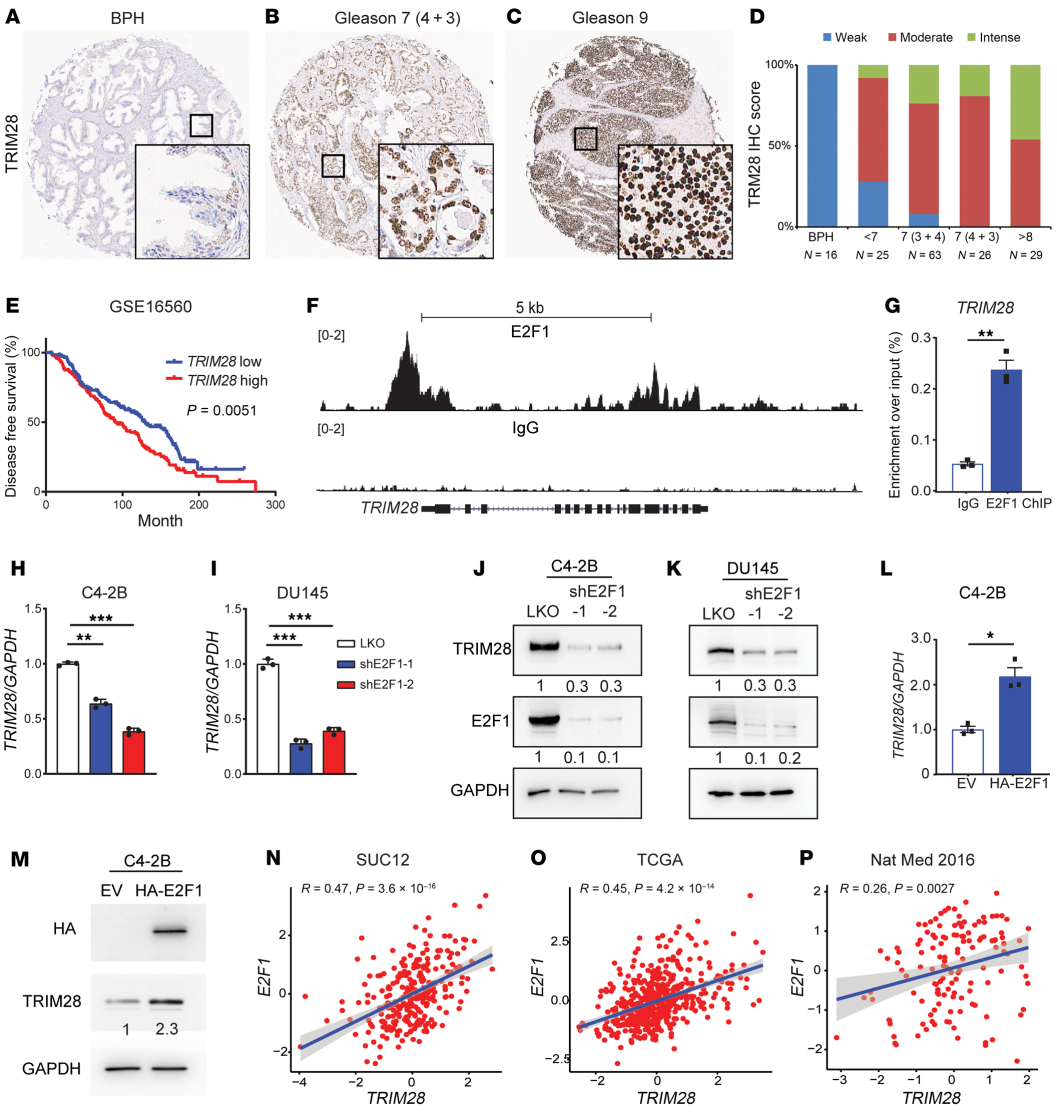
TRIM28 is an *E2F1* target in advanced prostate cancer. (**A**–**C**) Tissue microarray was subjected to IHC staining with anti-TRIM28 antibody. Representative images of patient samples at different disease stages are shown: (**A**) BPH, (**B**) Gleason 7, and (**C**) Gleason 9. Original magnification ×4; insets ×20. (**D**) Quantification of TRIM28 IHC intensity scores in BPH, Gleason score < 6, 3 + 4, 4 + 3, and > 8. The y-axis shows the percentage of tumors with weak (blue), moderate (red), and intense (green) IHC scores for each category. (**E**) Kaplan–Meier plot depicts disease-free survival of PCa patients stratified by *TRIM28*-high (above median value; red) and -low expression (below median value; blue) (*n* = 280). Significant differences between groups was determined by 1-way ANOVA. (**F** and **G**) Genome browser tracks indicate E2F1 CUT&RUN-seq peak at the promoter region of *TRIM28* loci (**F**). IgG and E2F1 ChIP-PCR were performed in LNCaP cells. qPCR data are shown as mean ± SEM, *n* = 3. Two-tailed unpaired Student’s *t* test, **P < 0.01. (**H**–**K**) C4-2B and DU145 cells were infected by 2 shRNAs targeting E2F1. RNA was harvested for qPCR analysis of TRIM28 mRNA levels (**H** and **I**) while protein lysates were subjected to immunoblot analysis (**J** and **K**). qPCR data are shown as mean ± SEM, *n* = 3. Statistical analysis was performed using a 2-tailed unpaired Student’s *t* test, with the Holm-Bonferroni method applied to correct for multiple comparisons. **P < 0.01, ***P < 0.001. (**L** and **M**) C4-2B cells were transiently transfected with empty vector (EV) and HA-E2F1 followed by qPCR (**L**) and IB (**M**) analysis against HA and TRIM28. qPCR data are shown as mean ± SEM, *n* = 3. Two-tailed unpaired Student’s *t* test, *P < 0.05. (**N**–**P**) E2F1 RNA (y-axis) was plotted against TRIM28 RNA (x-axis) using the SUC12 (*n* = 266) (**N**), TCGA (*n* = 492) (**O**), and Nat Med 2016 (*n* = 136) (**P**) datasets. x- and y-axes show normalized expression. Statistical analysis is based on linear regression.

We previously reported that *TRIM28* mRNA is upregulated in advanced PCa compared with benign and/or localized PCa (25). To comprehensively investigate TRIM28 protein expression, we performed TRIM28 IHC staining in a large cohort of clinical PCa samples. We confirmed that TRIM28 IHC staining shows a prominent nuclear localization, consistent with its role in transcriptional regulation (Figure 1, A–C). To evaluate the protein expression of TRIM28 at varying stages of PCa progression, we analyzed TRIM28 IHC staining intensity in benign prostatic hyperplasia (BPH) (*n* = 16), Gleason score < 7 (*n* = 25), 7 [3 + 4] (*n* = 63), 7[4 + 3] (*n* = 26) and > 8 (*n* = 29). We noticed that approximately 50% of high-grade PCa tumors (Gleason score > 8) showed intense TRIM28 staining (Figure 1D). By contrast, the vast majority of low-grade PCa tumors express a moderate-to-low amount of TRIM28 (Figure 1D). From PCa patient database (30), patients with *TRIM28*-high expression experienced significantly worse disease-free survival (Figure 1E and Supplemental Figure 1A; supplemental material available online with this article; https://doi.org/10.1172/JCI185119DS1) and showed a trend of worse overall survival (Supplemental Figure 1B). To elucidate the upstream mechanism leading to aberrant *TRIM28* expression in advanced PCa, we investigated potential TF binding at the *TRIM28* gene locus using ENCODE Transcription Factor Binding tracks, and the E2F1 CUT&RUN-seq peak was found at the promoter region (Supplemental Figure 1C). The E2F TF family is highly expressed in advanced PCa (4, 5). To examine whether E2F1 is the critical TF that drives *TRIM28* gene expression in advanced PCa, we performed E2F1 CUT&RUN-seq in LNCaP cells. Our data demonstrated robust E2F1 enrichment over IgG at *TRIM28* gene loci, which is validated by E2F1 ChIP-PCR analysis (Figure 1, F and G). In addition, KD of *E2F1* expression in C4-2B and 22Rv1 cells (AR-positive CRPC) and DU145 and PC3 (AR-negative cells) by 2 independent shRNA targeting E2F1 revealed a concordant reduction of *TRIM28* mRNA and protein level in qPCR and immunoblot (IB) analysis (Figure 1, H–K and Supplemental Figure 1, D–G). Conversely, TRIM28 gene and protein expression show an increase in C4-2B cells transiently overexpressing E2F1 (Figure 1, L and M). Furthermore, we observed a significant positive correlation of *E2F1* and *TRIM28* in multiple PCa clinical datasets (Figure 1, N–P). Taken together, our results implicate that E2F1 targets *TRIM28* and leads to *TRIM28* aberrant expression in advanced PCa.

### TRIM28 regulates the E2F pathway in advanced prostate cancer.

**Figure 2:**
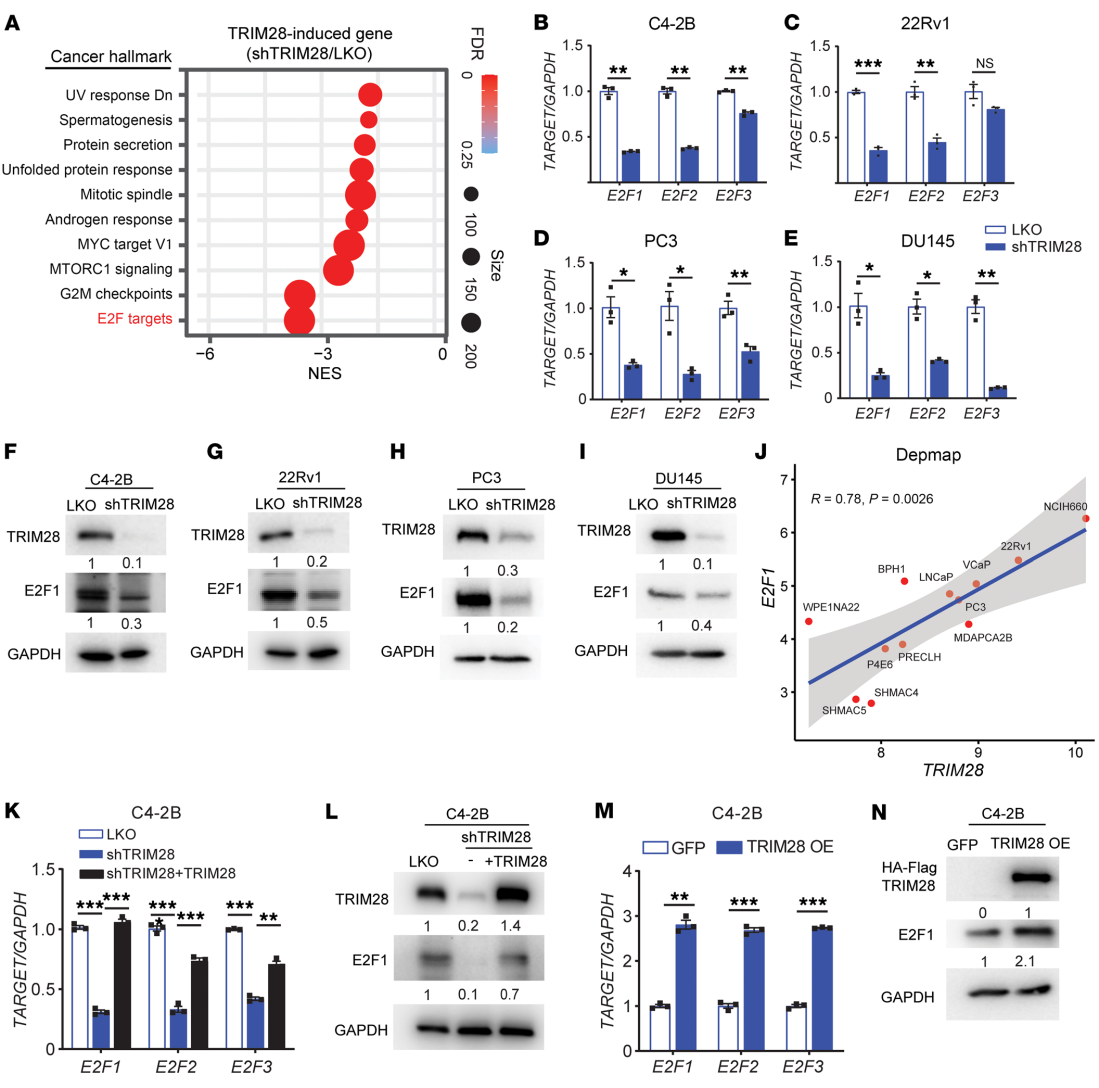
TRIM28 regulates the E2F pathway in CRPC. (**A**–**I**) TRIM28 induces the E2F pathway. RNA-Seq was performed using RNA extracted from C4-2B with LKO and shTRIM28, respectively. GSEA enrichment plots reveal various Cancer Hallmark signatures are induced by TRIM28 (**A**). C4-2B (**B** and **F**), 22Rv1 (**C** and **G**), PC3 (**D** and **H**), and DU145 (**E** and **I**) cells were infected by shRNA targeting *TRIM28*. RNA was harvested for qPCR analysis of *E2F* levels (**B**–**E**) while protein lysates were subjected to immunoblot analysis against TRIM28 and E2F1 (**F**–**I**). qPCR data are shown as mean ± SEM, *n* = 3. Two-tailed unpaired Student’s *t* test, *P < 0.05, **P < 0.01, ***P < 0.001. (**J**) *E2F1* and *TRIM28* gene expression in prostate cell line at different stages was queried from Depmap portal and presented in a scatter plot. Statistical analysis is based on linear regression. (**K** and **L**) shRNA-resistant TRIM28 plasmids were introduced into C4-2B cells with shTRIM28. RNA was harvested for qPCR analysis of *E2F* levels (**K**) while protein lysates were subjected to immunoblot analysis against TRIM28 and E2F1 (**L**). qPCR data are shown as mean ± SEM, *n* = 3. Statistical analysis was performed using a two-tailed unpaired Student’s *t* test, with the Holm-Bonferroni method applied to correct for multiple comparisons. **P < 0.01, ***P < 0.001. (**M** and **N**) Overexpression of TRIM28 upregulates *E2F1* expression. C4-2B cells stably expressing HA-Flag tagged TRIM28 were collected for qPCR (**M**) or immunoblot (**N**). qPCR data are shown as mean ± SEM, *n* = 3. Two-tailed unpaired Student’s *t* test, *P < 0.05, **P < 0.01, ***P < 0.001.

Given its primary role in transcriptional regulation, the downstream pathways elicited by TRIM28 remain unclear in advanced PCa. To elucidate the role, we performed RNA-seq analysis using C4-2B cells with LKO (control) and *TRIM28* KD. By interrogating TRIM28 transcriptome using Cancer Hallmark signature, we nominated E2F Target as the most significant hallmark induced by TRIM28 (Figure 2A). Since E2F1–E2F3 are the critical transcription activators in the E2F pathway and the drivers for cell cycle and DNA replication–related gene expression (31, 32), we tested whether *E2F1*–*E2F3* are the transcriptional targets of TRIM28. Indeed, our qPCR analysis showed that *TRIM28* KD resulted in a significant reduction of *E2F1*–*E2F3* mRNA levels in C4-2B and 22Rv1 cells (AR positive), PC3, and DU145 cells (AR negative) (Figure 2, B–E). Consistently, IB revealed that depletion of TRIM28 leads to concordant reduction of the E2F1 protein in all cell lines (Figure 2, F–I). These data suggest that TRIM28 regulates the E2F pathway independent of AR and Rb status. Using another approach, we knocked out *TRIM28* using the CRISPR/Cas-9 system and IB demonstrated that *TRIM28* KO leads to a concordant reduction of E2F1 (Supplemental Figure 2A). To confirm the robustness of this regulation, we queried *E2F1* and *TRIM28* gene expression using the DepMap portal and observed a significant positive correlation in 12 prostate cells at different disease stages (Figure 2J). To demonstrate the on-target effect of TRIM28 shRNA, we performed a rescue experiment in C4-2B cells. Introduction of shRNA-resistant TRIM28 plasmid in *TRIM28*-KD cells largely rescued the mRNA and protein levels of E2F1 (Figure 2, K and L). In addition, we examined the effect of TRIM28 OE in C4-2B cells. qPCR and IB revealed a concordant increase in *E2F1* mRNA and protein levels (Figure 2, M and N). To sum up, these data suggest that E2F1-targeted TRIM28 forms a positive feedback loop to upregulate *E2F1* expression in advanced PCa.

### TRIM28 regulates E2F1 genomic function through transcriptional activation of E2F1.

**Figure 3:**
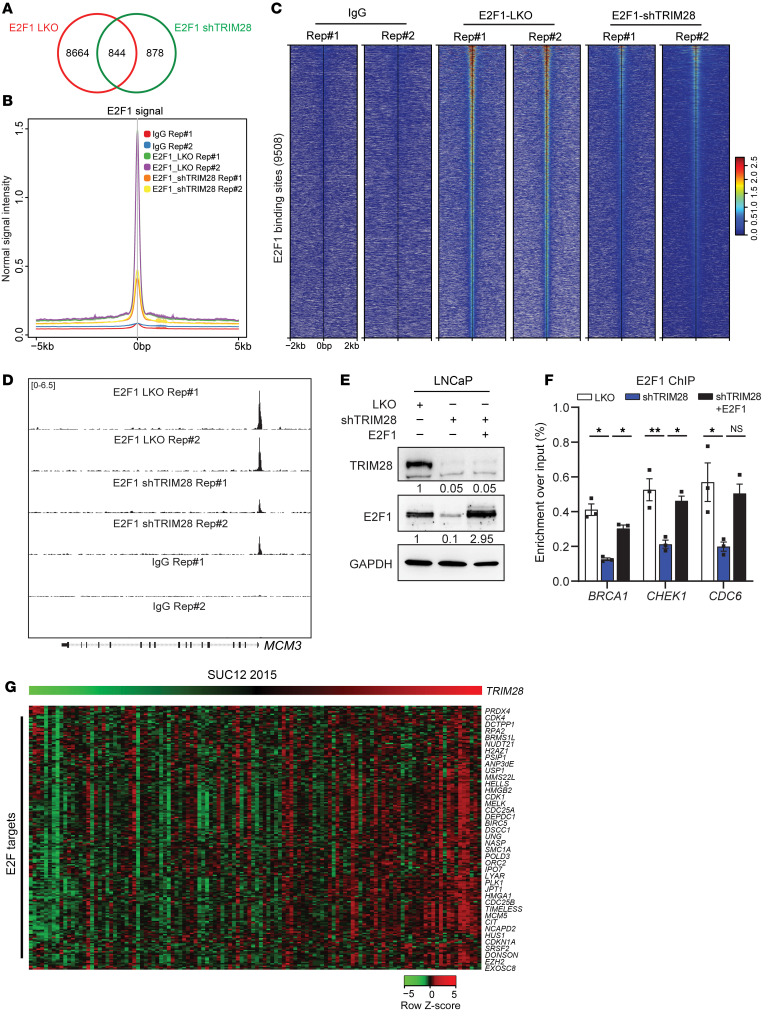
TRIM28 controls E2F1 genomic binding and E2F target expression in CRPC. (**A**–**D**) TRIM28 regulates E2F1 cistrome in CRPC. E2F1 CUT&RUN-seq was performed using LNCaP cells with LKO and shTRIM28. E2F1 peak was called by MACS2. Venn diagram indicates the overlapping of E2F1 binding sites for each treatment (**A**). Intensity plot depicts the CUT&RUN-seq peak intensity around peak center ±5 kb (**B**). Heatmaps indicate E2F1 CUT&RUN-seq signal at E2F1 binding sites ±2 kb (**C**). Genome browser tracks indicate E2F1 enrichment at the *MCM3* genome in the replicates of LKO and shTRIM28. IgG CUT&RUN-seq as negative control (**D**). (**E** and **F**) E2F1-ChIP was performed in LNCaP cells with indicated treatment (**E**), and E2F1 enrichment at representative E2F targets was evaluated by qPCR (**F**). qPCR data are shown as mean ± SEM, *n* = 3. Statistical analysis was performed using a two-tailed unpaired Student’s *t* test, with the Holm-Bonferroni method applied to correct for multiple comparisons. *P < 0.05, **P < 0.01, ns: not significant. (**G**) Heatmap showing the levels of E2F-target genes in the CRPC samples from SUC12 2015 study (*n* = 118) sorted by TRIM28.

To investigate whether *TRIM28* KD affects the E2F1 cistrome, we performed replicated E2F1 CUT&RUN-seq in LKO and *TRIM28*-KD LNCaP cells. Our CUT&RUN-seq analysis indicated that the number of E2F1 binding sites and the signal intensity are dramatically reduced upon *TRIM28* KD (Figure 3, A and B). A replicated heatmap showed that IgG control displays minimal signaling at E2F1 binding sites, pinpointing the specificity of our E2F1 CUT&RUN-seq (Figure 3C). For instance, we observed a remarkable reduction of E2F1 enrichment at the *MCM3* loci upon *TRIM28* KD (Figure 3D). To demonstrate this effect in Rb-deficient condition, we performed E2F1 CUT&RUN-seq experiment in LNCaP cells with sh*Rb* (Supplemental Figure 3A). Similarly, we observed a dramatic reduction of E2F1 binding sites and signal intensity upon *TRIM28* KD (Supplemental Figure 3, B–D). To address the impact of AR signaling on TRIM28’s regulation of *E2F1*, we conducted E2F1 ChIP-PCR in 22Rv1 (AR-positive CRPC) and DU145 (AR negative) cell lines, and our data confirmed that the enrichment of E2F1 at its gene targets is significantly reduced by *TRIM28* KD regardless of AR status (Supplemental Figure 3, E and F). To clarify whether *TRIM28* KD reduces E2F1 binding on target genes by downregulating *E2F1* gene expression or affecting the binding affinity of E2F1 to target genes, we performed E2F1 ChIP-PCR in LKO, *TRIM28* KD and E2F1 rescue of *TRIM28*-KD cells. Our data showed that the reduced genomic occupancy of E2F1 in *TRIM28*-KD condition can be rescued by overexpressing E2F1, suggesting that genomic binding of E2F1 may not require TRIM28 (Figure 3, E and F). Regarding E2F-target expression, qPCR analysis indicated that the expression of representative E2F targets (*BUB1B*, *CDC20*, *PCNA*, and *CENPM*) are downregulated by *TRIM28* KD, which can be reversed by E2F1 overexpression (Supplemental Figure 3, G–I). To corroborate our observation in clinical specimens, our heatmap analysis indicated that E2F-target expression exhibited upregulation in patient samples with *TRIM28*-high expression, compared with *TRIM28*-low expression from patients with CRPC (Figure 3G and Supplemental Figure 3J). In aggregate, these data indicate that TRIM28 positively regulates the E2F pathway through promoting the transcriptional activation of *E2F1*.

### pS473-TRIM28 promotes transcriptional activation of E2F1.

**Figure 4:**
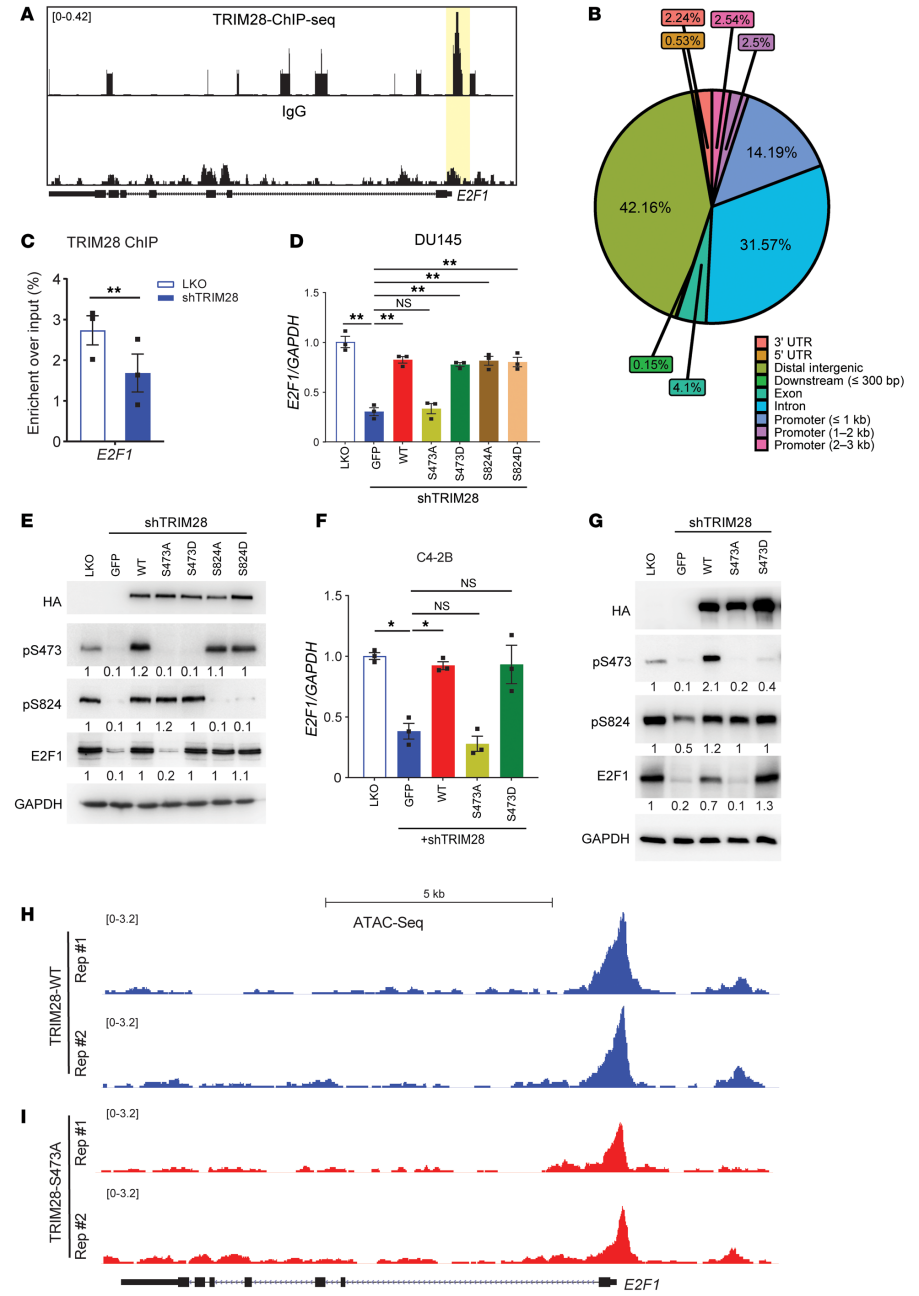
Phosphorylation-dependent TRIM28 transcriptional activation of E2F1. (**A**–**C**) TRIM28 directly occupies the promoter region of E2Fs. TRIM28 CUT&RUN-seq was performed in C4-2B cells. Genome browser tracks indicate TRIM28 CUT&RUN-seq peak at the promoter region of *E2F1* loci (**A**). Pie chart depicting the genome distribution of TRIM28 binding sites (**B**). TRIM28 ChIP was performed in LKO and shTRIM28 KD C4-2B cells. ChIP-PCR was performed using ChIPed-DNA and normalized to input (**C**). qPCR data are shown as mean ± SEM, *n* = 3. Two-tailed unpaired Student’s *t* test, **P < 0.01. (**D**–**G**) pS473-TRIM28 promotes transcriptional activation of its genomic targets. C4-2B and DU145 cells stably expressing HA-Flag-tagged GFP, TRIM28-WT, TRIM28-S473A, TRIM28-S473D, TRIM28-S824A, and TRIM28-S824D were treated by LKO or shTRIM28 as indicated. Four days after infection, RNA was harvested for qPCR analysis targeting *E2F1* (**D** and **F**) while protein lysates were harvested for immunoblot against HA, pS473-TRIM28, pS824-TRIM28, E2F1, and GAPDH (**E** and **G**). qPCR data are shown as mean ± SEM, *n* = 3. Statistical analysis was performed using a 2-tailed unpaired Student’s *t* test, with the Holm-Bonferroni method applied to correct for multiple comparisons. *P < 0.05, **P < 0.01. (**H** and **I**) pS473-TRIM28 facilitate chromatin accessibility at *E2F1* loci. ATAC-seq was performed in C4-2B cells with TRIM28-knockdown reexpressing TRIM28-WT (**H**) and TRIM28-S473A (**I**).

Despite TRIM28’s longstanding transcriptional repressive function, emerging evidence suggests that TRIM28 site-specific phosphorylation (S473 and S824) may serve as a molecular switch to convert it from transcriptional repressor into activator (20). Therefore, we hypothesize that TRIM28 site-specific phosphorylation may be the underlying mechanism that contributes to transcriptional activation of *E2F1*. To address whether *E2F1* is the direct target of TRIM28, we performed TRIM28 CUT&RUN-seq in C4-2B cells. Our results indicated there is robust TRIM28 occupancy over IgG at the promoters of *E2F1* loci (Figure 4A) and promoter occupancy accounts for approximately 20% of TRIM28’s global binding pattern, compared with less than 8% in IgG control. A Fisher’s exact test confirmed that this difference is highly significant (*P* < 2.2 × 10^–16^), with an odds ratio of 2.76. (Figure 4B and Supplemental Figure 4A). To validate this observation, we performed TRIM28 ChIP-PCR in LKO and *TRIM28*-KD cells. Our data demonstrated that enrichment of TRIM28 is reduced in *TRIM28*-KD cells, indicating the direct regulation of *E2F1* by TRIM28 (Figure 4C). Besides, TRIM28 genomic binding sites are enriched with several TF motifs, which may regulate the genomic binding of TRIM28 (Supplemental Figure 4B). Next, we attempted to determine whether pS473/pS824-TRIM28 promotes target gene transcriptional activation. To this aim, we conducted qPCR using RNA extracted from DU145 cells with *TRIM28*-KD rescued with TRIM28 WT, phosphodeficient mutants (S473A and S824A), and phosphomimic mutants (S473D and S824D). Our data demonstrated that TRIM28-S473A mutant fails to rescue the expression of *E2F1* mRNA and protein, whereas TRIM28-S473D fully recovers the *E2F1* gene and protein level to that of LKO cells (Figure 4, D and E). Noticeably, TRIM28-S824A and -S824D retaining pS473 also rescued *E2F1* gene and protein expression. A similar result was observed in C4-2B cells (Figure 4, F and G). To ensure scientific rigor, IB analysis showed that HA–Flag-tagged TRIM28 mutants express at similar levels, ruling out the possibility that the mutants affect TRIM28 function due to their difference in protein stability (Figure 4, E and G). To explore the mechanisms by which pS473-TRIM28 enhances transcriptional activation, we conducted ATAC-seq in C4-2B cells with *TRIM28* KD that were reexpressing TRIM28-WT and S473A mutants. Our data revealed that ATAC-seq signaling at the promoter region of *E2F1* and its target genes, such as *BRCA1*, *CDC6* and *CHEK1* loci, are remarkably reduced in S473A mutant compared with WT cells (Figure 4, H and I and Supplemental Figure 4, A–C). In addition, H3K4me3 (active promoter) and H3K27Ac (active promoter + enhancer) ChIP-PCR were performed using TRIM28-WT/S473A reconstituted cells. Indeed, *E2F1* loci bound by TRIM28-WT exhibited a significantly higher H3K4me3 and H3K27Ac enrichment than that of S473A (Supplemental Figure 4, D and E). To test whether S473 phosphorylation could potentially affect TRIM28 chromatin binding, we performed TRIM28 CUT&RUN-seq in C4-2B cells with *TRIM28*-KD that were rexpressing TRIM28-WT and S473A mutants. Our data indicated that the S473A mutant, in comparison with the WT cells, exhibited reduced genomic binding sites (Supplemental Figure 4F). In addition, we observed that there was genomic reprogramming of the S473A mutant (Supplemental Figure 4, G–I), suggesting that TRIM28-S473 phosphorylation may direct TRIM28 to distinct genomic loci. It remains to be investigated whether this is mediated by an association with other TFs. Collectively, these results imply that TRIM28 S473 phosphorylation may facilitate chromatin remodeling and its target gene transcriptional activation.

### RSK1 kinase directly phosphorylates TRIM28 at S473.

**Figure 5:**
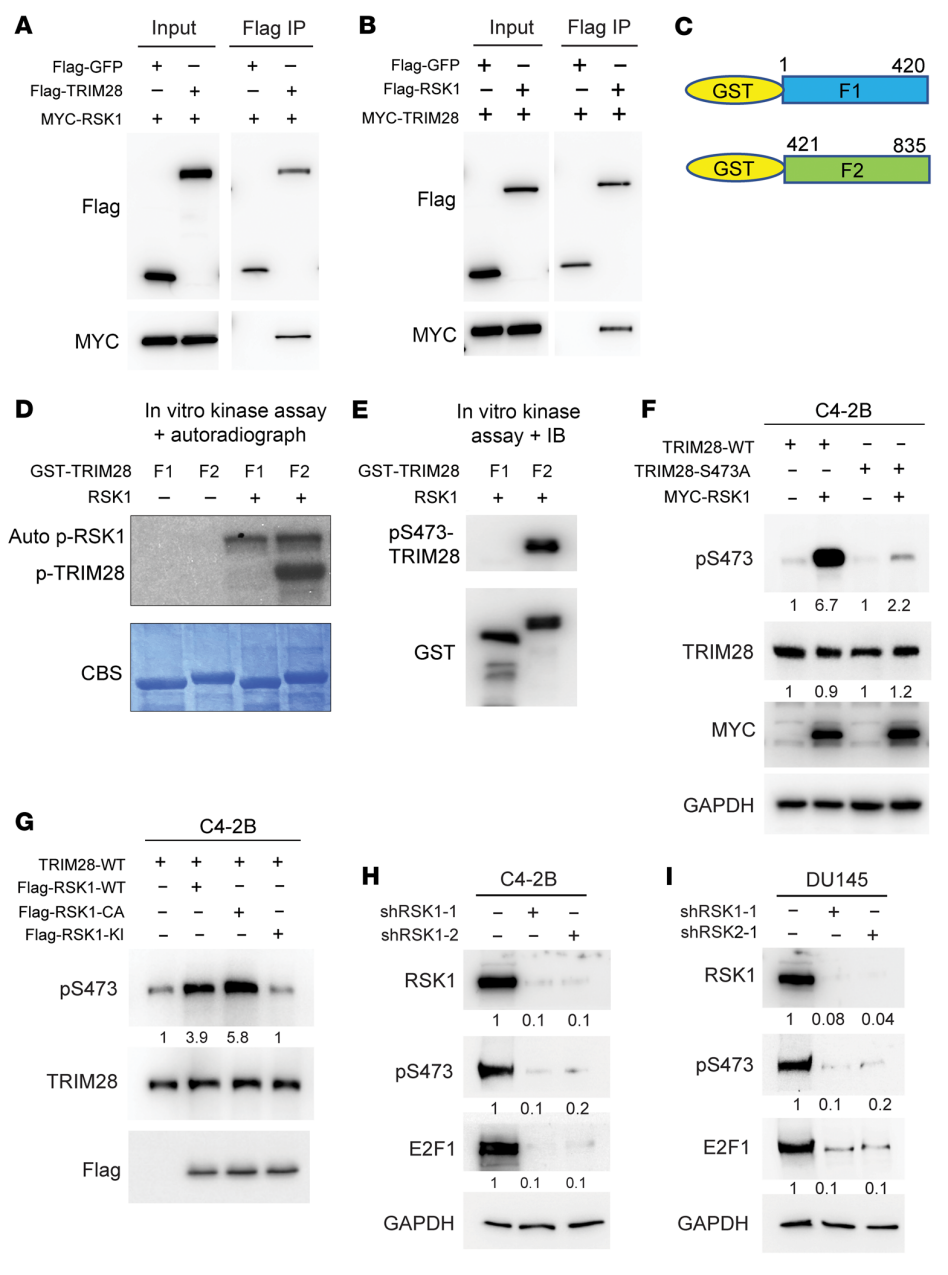
RSK1 directly phosphorylates TRIM28 at S473 in CRPC. (**A** and **B**) RSK1 interacts with TRIM28 in cells. Co-IP was performed in 293T cell lysates expressing MYC-RSK1 along with HA-Flag GFP fusion (as negative control) or HA-Flag TRIM28 (**A**), MYC-TRIM28 along with HA-Flag GFP fusion or HA-Flag RSK1 (**B**) using Flag antibody. The eluted protein was analyzed by immunoblot. (**C**–**E**) RSK1 phosphorylates TRIM28 at S473 in vitro. Using *E.coli*-expressed GST-TRIM28 fragments (F1-F2) (**C**), in vitro kinase assay was performed in presence of γ-^32^P–labelled ATP and with the use of an autoradiograph to detect protein phosphorylation (**D**) or in the presence of unlabeled ATP and with the use of immunoblotting to detect pS473-TRIM28 (**E**). (**F** and **G**) RSK1 phosphorylates TRIM28 at S473 in PCa cells. C4-2B cells transiently expressing MYC-RSK1 along with TRIM28-WT or TRIM28-S473A (**F**); MYC-TRIM28 along with Flag-RSK1-WT, -CA (constitutively active) and -KI (kinase-inactive) were harvested for immunoblot (**G**). (**H** and **I**) Protein lysates of C42B and DU145 cells with LKO and 2-independent shRSK1 were collected for immunoblot.

By performing phosphorylation motif prediction using KinasePhos 2.0, we identified RSK1 as a potential kinase that phosphorylates TRIM28 at S473. To test whether there is interaction between RSK1 and TRIM28 in cells, we performed co-IP assay using Flag-antibody in 293T cells transiently expressing MYC-RSK1 along with GFP and HA-Flag TRIM28, and IB showed that RSK1 is readily co-IPed by TRIM28 and vice versa (Figure 5, A and B). In addition, we performed GST pull–down assay using recombinant RSK1 and TRIM28 fusion proteins. IB showed that both TRIM28-fragments (F1/F2) directly interact with RSK1 (Figure 5C and Supplemental Figure 5A). Next, we conducted an in vitro kinase assay and an autoradiograph. A robust phosphorylation was detected exclusively in TRIM28-F2, where S473 is located (Figure 5D). To substantiate this observation, IB confirmed that RSK1 phosphorylated TRIM28 at S473 in vitro (Figure 5E). To demonstrate whether RSK1 phosphorylated TRIM28 in CRPC cells, we transiently expressed MYC-RSK1 along with TRIM28-WT or -S473A mutant in C4-2B cells. IB illustrated that pS473 level was elevated in TRIM28-WT but not TRIM28-S473A upon RSK1 OE (Figure 5F). To address whether RSK1 kinase activity was required to phosphorylate TRIM28, we transiently expressed MYC-TRIM28 along with Flag-RSK1-WT, -CA (constitutively-active), and -KI (kinase-inactive) mutants in C4-2B cells. IB revealed that pS473 level is dramatically elevated in CA mutant–expressing cells while it is almost abolished in KD mutant–expressing cells (Figure 5G). Next, we sought to examine whether RSK1 is required for phosphorylating TRIM28-S473 at the endogenous level. IB revealed that pS473 is readily detected in DU145 and C4-2B cells, while it is largely reduced upon *RSK1*-KD (Figure 5, H and I). To examine whether other RSK family members are involved in regulating TRIM28 S473 phosphorylation, we performed *RSK2*-*RSK4* KD in C4-2B and DU145 cells (Supplemental Figure 5, B–G), and our result indicated that RSK1 is the only RSK kinase family member to regulate TRIM28 S473 phosphorylation. To summarize, these results demonstrate that RSK1 phosphorylated TRIM28 at S473 in CRPC.

### pS473-TRIM28 promotes CRPC progression.

**Figure 6:**
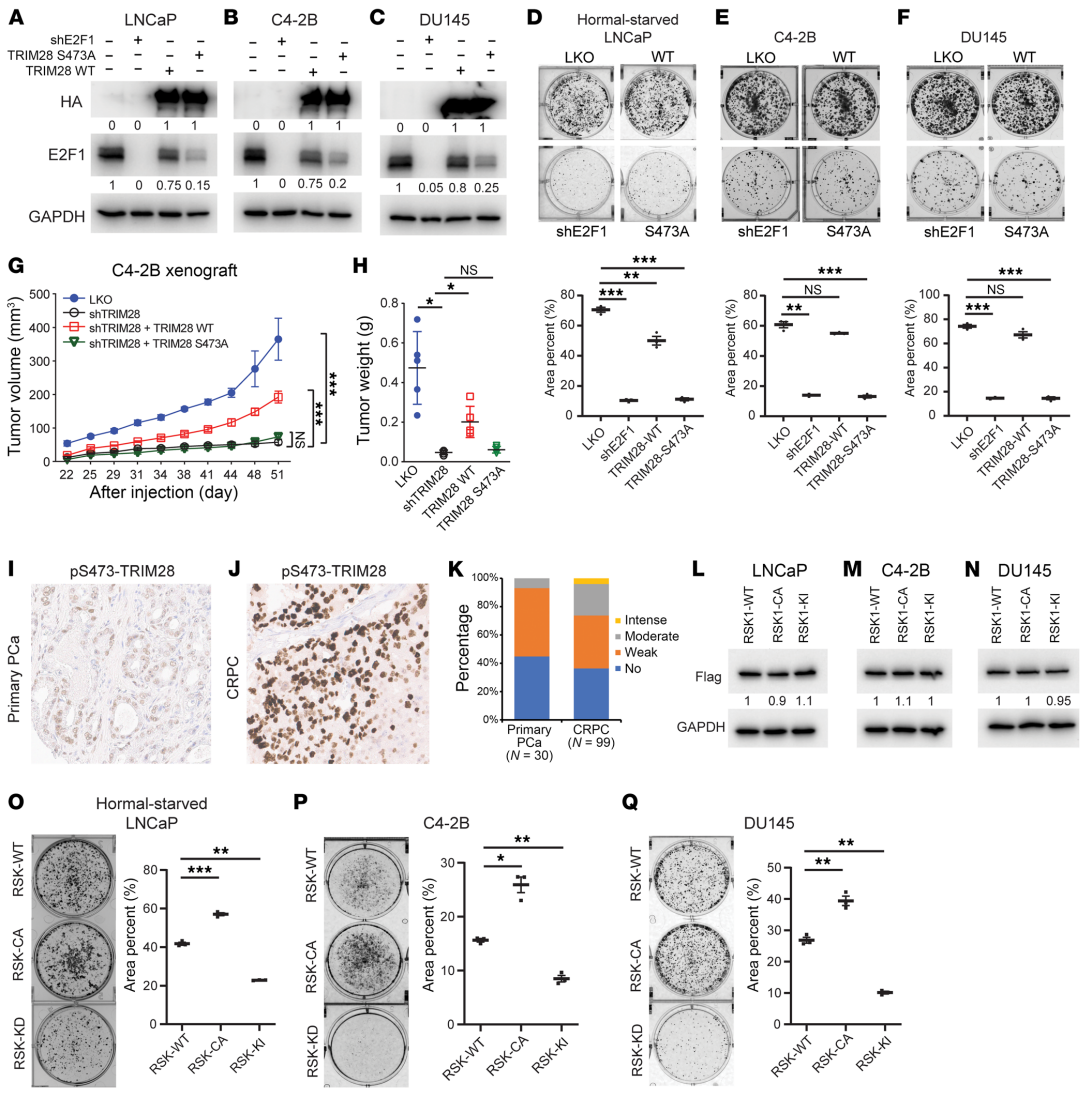
pS473-TRIM28 is required for CRPC progression. (**A**–**F**) pS473-TRIM28 promotes CRPC growth. LNCaP grown in hormone-depleted medium, C4-2B, and DU145 cells with the indicated treatment were harvested for immunoblot (**A**–**C**) and analyzed with the colony formation assay for 7–14 days, followed by fixation and crystal violet staining (**D**–**F**). Quantification was conducted by image J (colony area plugin) and presented as mean ± SEM, *n* = 3. Statistical analysis was performed using a 2-tailed unpaired Student’s *t* test, with the Holm-Bonferroni method applied to correct for multiple comparisons. **P < 0.01, ***P < 0.001. (**G** and **H**) Xenograft assay was performed by inoculating NSG mice with C4-2B LKO, *TRIM28*-KD, and *TRIM28*-KD cells rescued by TRIM28-WT and S473A mutant. (**G**) The tumor volume data were presented as mean ± SEM, *n* = 5. A 1-way ANOVA followed by post hoc multiple comparisons was used for analysis. P value adjustment was performed using Tukey’s Honest Significant Difference (HSD) test. ***P < 0.001. (**H**) Tumor weight data were presented as mean ± SEM, *n* = 5. Statistical tests performed were 2-tailed unpaired Student’s t-test, with the Holm-Bonferroni method applied to correct for multiple comparisons. *P < 0.05. (**I**–**K**) Tissue microarray constructed from primary PCa and metastatic CRPC were subjected to IHC staining using anti-pS473–TRIM28 antibody. Representative images and IHC quantification of patient samples at each disease stage were shown. (**L**–**Q**) RSK1 kinase activity is required for CRPC growth. Hormone-starved LNCaP, C4-2B, and DU145 cells with the indicated treatment were harvested for immunoblot (**L**–**N**) and subjected to the colony formation assay for 7–14 days (**O**–**Q**). Quantification was conducted by image J (colony area plugin) and presented as mean ± SEM, *n* = 3. Statistical analysis was performed using a 2-tailed unpaired Student’s *t* test, with the Holm-Bonferroni method applied to correct for multiple comparisons. *P < 0.05, **P < 0.01, ***P < 0.001.

To determine the impact of the RSK1–TRIM28–E2F1 axis on the progression of castration resistance, we performed colony formation and cell proliferation assays by using androgen-dependent PCa line LNCaP cells grown in hormone-depleted (charcoal stripped-FBS) medium, which mimics castration. Our data suggested that the TRIM28-S473A mutant and *E2F1* KD (Figure 6, A and D and Supplemental Figure 6A) attenuate the cell proliferation and colony formation of LNCaPs in a hormone-deprived environment, compared with the control and TRIM28-WT rescue conditions. This highlights the importance of the RSK1–TRIM28–E2F1 axis on the progression of castration resistance in PCa. To determine the physiological relevance of pS473-TRIM28 in CRPC, we used DU145 and C4-2B cells to examine the functional effect of E2F1 and TRIM28 S473 phosphorylation (Figure 6, B and C). Our results indicated that both *E2F1*-KD (shE2F1) and rescuing shTRIM28 cells with phospho-deficient TRIM28-S473A significantly decreased colony formation (Figure 6, E and F) and cell proliferation (Supplemental Figure 6, B and C), as opposed to the control and TRIM28-WT rescue populations. To further evaluate the effect of TRIM28 S473 phosphorylation in vivo, we performed a xenograft assay by inoculating C4-2B cells expressing LKO, shTRIM28, TRIM28-WT and -S473A mutant in *TRIM28*-KD background into castrated NSG mice. Our data revealed that *TRIM28*-KD significantly reduced xenograft tumor size, which can be rescued by TRIM28-WT but not TRIM28-S473A (Figure 6, G and H and Supplemental Figure 6, D and E). To demonstrate that the mechanism of RSK1–TRIM28–E2F1 regulation is *RB1* independent, we used WT 22Rv1 cells and CRISPR-mediated *RB1* KO 22Rv1 cells. Our data showed that *E2F1*-KD and TRIM28-S473A also attenuate cell colony formation in either condition (Supplemental Figure 6, F–I), suggesting that RSK1–TRIM28–E2F1 regulation is *RB1*-independent. To examine the gain of function of TRIM28 in CRPC, our data show that TRIM28 OE in hormone-starved LNCaP, C4-2B, and DU145 cells promotes CRPC growth (Supplemental Figure 6, J–R). To demonstrate the clinical relevance of pS473-TRIM28, we performed IHC staining using primary PCa and metastatic CRPC specimens. Our result revealed that greater than 25% of CRPC exhibit moderate/intense pS473-TRIM28 nuclear staining, compared with 6.7% seen in primary PCa (Figure 6, I–K). This implicates that the pS473-TRIM28 protein level is elevated during PCa progression. Collectively, these results indicate that pS473-TRIM28 accelerates CRPC progression.

### RSK1 kinase activity is required for CRPC cell proliferation.

To investigate the clinical relevance of RSK1, we reanalyzed a patient cohort and found *RSK1* gene expression to be significantly elevated from PCa to CPRC (Supplemental Figure 7A). In 3 published patient datasets, *TRIM28* and *RSK1* gene expression displayed a significantly positive correlation (Supplemental Figure 7, B–D). To verify if RSK1 kinase activity is required for CRPC cell proliferation, we performed colony formation in hormone-starved LNCaP, C4-2B, and DU145 cells, expressing RSK1 WT, CA, or KI mutants. Our data revealed that RSK1-WT– and CA-expressing cells have more colony formation than the *RSK1*-KI mutant (Figure 6, L–Q), implicating the requirement of RSK1-kinase activity in CRPC cell proliferation. Given that RSK1 kinase has numerous substrates, it is important to determine if TRIM28 and E2F1 mediate these effects. To this end, we performed a colony formation assay in hormone-deprived LNCaP, C4-2B, and DU145 cells overexpressing empty vector, RSK1 alone, and RSK1 along with TRIM28-S473A and shE2F1. Our data showed that RSK1 OE promotes cell colony formation relative to vector control. This phenomenon is substantially mitigated by TRIM28-S473A replacement or *E2F1*-KD (Supplemental Figure 7, E–J), indicating that TRIM28 and E2F1 partially mediate the oncogenic function of RSK1.

### RSK inhibition abolishes pS473-TRIM28.

**Figure 7:**
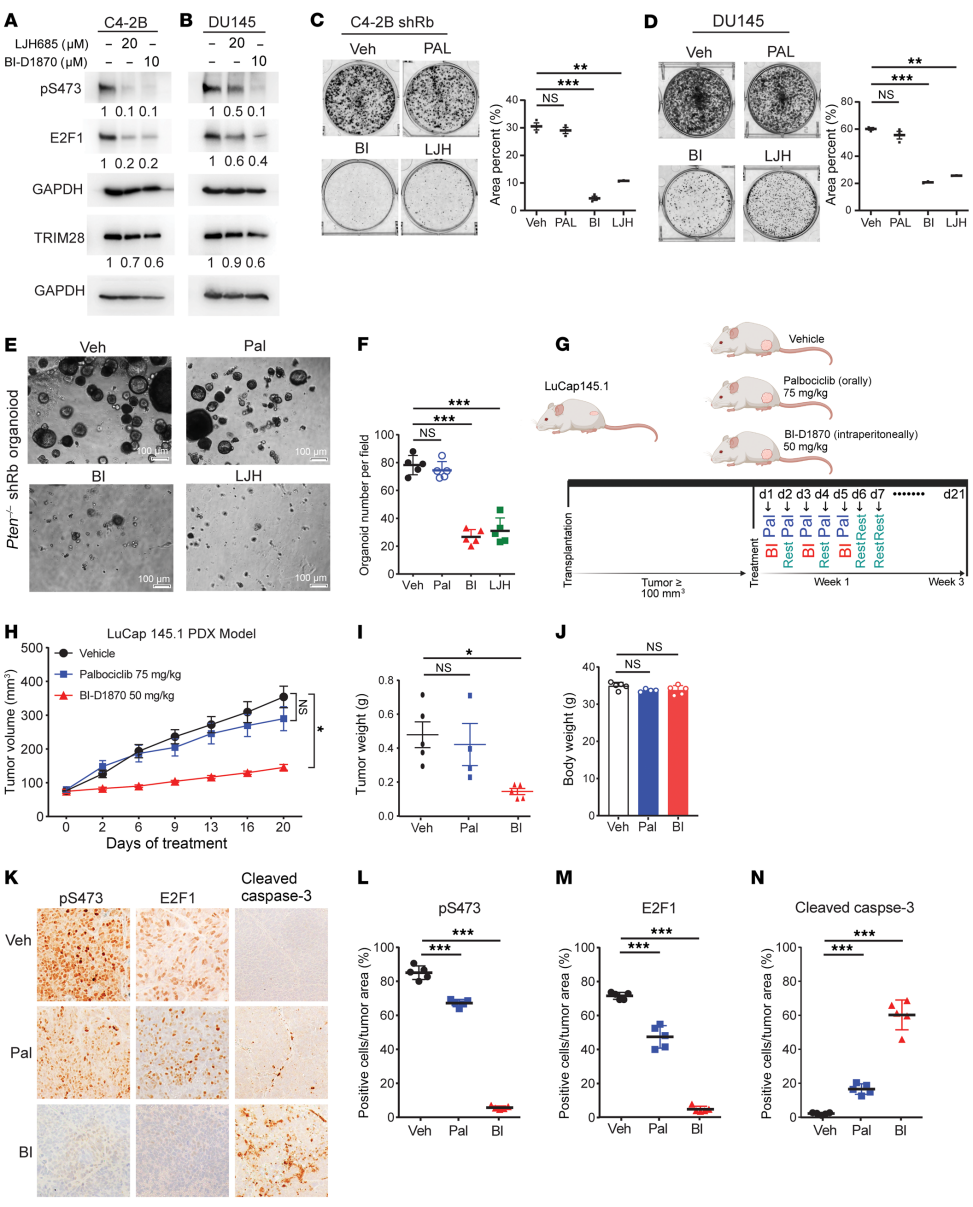
Exploitation of the RSK1-TRIM28–E2F1 axis as a vulnerability in Rb1-deficient prostate cancer. (**A** and **B**) C4-2B and DU145 cells treated with RSK inhibitors were analyzed by immunoblot. (**C** and **D**) C4-2B with *Rb* knockdown and DU145 cells treated by vehicle (Veh), 500 nM palbociclib (Pal), 1 μM BI-D1870 (BI), and 20 μM LJH685 (LJH) were subjected to the colony formation assay. Quantification was conducted by image J and presented as mean ± SEM, *n* = 3. Two-tailed unpaired Student’s *t* test, with the Holm-Bonferroni method applied to correct for multiple comparisons. **P < 0.01, ***P < 0.001. (**E** and **F**) PCa organoids were generated from prostate tumors in Pb-Cre:*Pten*^–/–^ mice. (**E**) Representative images were shown. (**F**) Quantification was presented as mean ± SEM, *n* = 3. Two-tailed unpaired Student’s *t* test, with the Holm-Bonferroni method applied to correct for multiple comparisons. ***P < 0.001. (**G**) The experimental design of LuCaP145.1 PDX assay and treatment strategy. (**H**–**J**) NGS mice were implanted subcutaneously with LuCaP 145.1 tumors. Mice were randomized and treated with vehicle, palbociclib (75 mg/kg) and BI-D1870 (50 mg/kg) for 3 weeks. Tumor sizes were plotted against days of treatment (**H**). The tumor volume data were presented as mean ± SEM, *n* = 5. The statistical analysis was performed using a 1-way ANOVA with Tukey’s HSD test for multiple comparisons *P* value adjustment. ***P < 0.001. Tumor weight was presented as boxplot (**I**), and the toxicity was evaluated by mouse body weight (**J**). The data was presented as mean ± SEM, *n* = 5. Two-tailed unpaired Student’s *t* test, with the Holm-Bonferroni method applied to correct for multiple comparisons. *P < 0.05. (**K**–**N**) Tumor tissues were subjected to IHC assay. Magnification ×20. (**K**), followed by quantification (**L**–**N**). The data was presented as mean ± SEM, *n* = 5. Two-tailed unpaired Student’s *t* test, with the Holm-Bonferroni method applied to correct for multiple comparisons. ***P < 0.001.

To substantiate the observation that RSK kinase activity regulates pS473-TRIM28, IB revealed that RSK-specific inhibitors (BI-D1870 and LJH685 (26, 33, 34)) reduce pS473-TRIM28 as well as E2F1 protein expression in C4-2B and DU145 cells (Figure 7, A and B). To evaluate the on-target effect of RSK inhibitors on E2F1 expression, we expressed TRIM28-S473D in cells treated with BI-D1870. IB illustrated that TRIM28-S473D, but not TRIM28-S473A, is capable of rescuing E2F1 expression from BI-D1870 inhibition (Supplemental Figure 7A). As RSK1 has been known to be phosphorylated at T573 by ERK for its full kinase activity, we hypothesized that ERK inhibitor treatment would abolish RSK1-driven pS473-TRIM28. IB revealed that pT573-RSK1 and pS473-TRIM28 levels were downregulated by 24 hour treatment of ERK/RSK inhibitor (ulixertinib) (Supplemental Figure 7, B and C). Similarly, immunofluorescence (IF) showed that either RSK or ERK inhibition dramatically reduced nuclear staining of pS473-TRIM28 in C4-2B cells (Supplemental Figure 7D). These data suggest that pS473-TRIM28 could be targeted via inhibition of RSK1 activity using ERK/RSK inhibitor.

### Exploitation of the RSK1–TRIM28–E2F1 axis as a vulnerability in Rb1-deficient PCa.

CDK4/6 inhibitors control E2F activity by abolishing CDK-mediated Rb phosphorylation and are thus effective in the treatment of cancer with intact canonical Rb-E2F pathway. However, the loss of *RB1* in CRPC (10%–15%) not only deregulates E2F activity but also renders these tumors unamenable to CDK4/6 inhibitors. Our previous data demonstrated that RSK-driven pS473-TRIM28 promotes the transcriptional activation of *E2F1* regardless of Rb status (Figure 2), which prompts us to exploit the RSK1–TRIM28–E2F1 axis as a vulnerability in treating Rb-deficient PCa. To test this, we treated Rb-proficient C4-2B and 22Rv1 cells with a CDK inhibitor (palbociclib) and performed cell proliferation and colony formation assays. Our results showed that both cell lines are sensitive to palbociclib (Supplemental Figure 7, E and F), reinforcing the fact that CDK inhibition is effective in treating cancers with the canonical Rb-E2F1 pathway. Next, we treated DU145 (Rb-deficient) and C4-2B *Rb*-KD cells with vehicle, palbociclib, BI-D1870, and LJH685. Our results showed that Rb-deficient cells are insensitive to palbociclib (Figure 7, C and D and Supplemental Figure 7G), highlighting the need for identifying vulnerability in treating Rb-deficient PCa. Importantly, our results revealed that Rb-deficient cells are readily sensitive to a single treatment of BI-D1870 or LJH685 (Figure 7, C and D). Using an organoid model from PCa tumor tissue of Pb-Cre:*Pten*^–/–^ mice, in which *Rb* is further knocked down by shRNA (Supplemental Figure 7H), we demonstrated that this organoid is not sensitive to palbociclib treatment, while RSK inhibition remarkably reduces the number and size of organoids (Figure 7, E and F). To evaluate the efficacy of drug treatment in vivo, we employed patient-derived xenograft (PDX) model LuCaP 145.1, which is Rb-deficient (35). After implanted tumors grew approximately 100 mm^3^, mice were randomized to receive vehicle, palbociclib (75 mg/kg), or BI-D1870 (50 mg/kg) for 3 weeks (Figure 7G). Our data revealed that RSK but not CDK inhibition remarkably suppressed Rb-deficient tumor growth and weight in the PDX model (Figure 7, G–J). IHC was performed using the tumor tissue, and our data showed that pS473-TRIM28 was reduced upon RSK inhibitor treatment, which points to an on-target pharmacological effect. In addition, BI-D1870 induced cytotoxicity via apoptosis, as indicated by upregulated cleaved caspase 3 staining (Figure 7, K–N). Furthermore, we have analyzed RNA-seq data from Veh and BI-D1870–treated tumors, and GSEA analysis demonstrated that the RSK1 inhibitor treatment inhibited the E2F target and TRIM28-induced gene signature (Supplemental Figure 8, I and J). Additionally, we have performed E2F1 ChIP-qPCR on its target genes in 22Rv1 cells treated with Veh and BI-D1870. Our result indicated that the enrichment of E2F1 at primary targets was significantly reduced upon BI-D1870 treatment (Supplemental Figure 8K), suggesting that RSK1 inhibitors primarily target E2F1 signaling. As LuCaP 145.1 is regarded as a Rb-deficient neuroendocrine model, which is AR-negative, we sought to validate the RSK1-TRIM28-E2F1 signaling pathway by using Rb-deficient AR-positive models. To this aim, we performed a xenograft assay using AR-positive CRPC cell line 22Rv1 with *RB1* KO, followed by Veh, Palbociclib, and BI-1870 treatment. Similar to the LuCaP145.1 model, our results showed that the *RB1*-loss CRPC tumor is only sensitive to BI-1870 (Supplemental Figure 8, L–R). Altogether, our preliminary data implicate that the RSK1–TRIM28–E2F1 axis may be exploited as a vulnerability in treating Rb-deficient PCa.

## Discussion

TRIM28 is a member of the TRIM family, also known as KRAB-associated (Krüppel-associated box–associated) protein 1 (KAP1) or transcriptional mediator 1b (TIF1b), which plays a primary role in epigenetic and gene regulation. Over the years, TRIM28 was found to be amplified in most human cancers, and increased TRIM28 expression correlates with poor patient prognosis in multiple tumor types, including ovarian, lung, glioma, and prostate (25, 36–38). However, the mechanisms underlying TRIM28 upregulation in PCa are largely unknown, except for a few reports showing that *TRIM28* is a target of *miR-140-3p* and *miR491* in breast and glioma cancer, respectively (39, 40). Here, we show that *TRIM28* is a E2F1 target, which contributes to aberrant *TRIM28* expression in advanced PCa. Functionally, TRIM28 has been associated with CRPC cell growth and proliferation, which is partially attributed to TRIM24 protein stabilization (25). In the present study, we performed genome-wide expression profiling of PCa cells with *TRIM28* knockdown and reported that E2F is the key pathway induced by TRIM28. We further identified activator E2F1, which is also highly expressed in advanced PCa, as a key downstream mediator of the TRIM28 function. Therefore, we determined that TRIM28/E2F1 forms a positive feedback loop to control a wide spectrum for gene expression during CRPC progression.

Despite its longstanding function as a transcriptional repressor, site-specific phosphorylation (S473 and S824) has been shown to trigger the TRIM28 transcription repressor–activator switch (20). Our study data suggest that TRIM28 S473, rather than S824, phosphorylation promotes transcriptional activation of *E2F1* via increasing chromatin accessibility at the promoter region of *E2F1*. This implies that TRIM28 site-specific phosphorylation may drive a distinct gene transcription program. Until now, a handful of studies have reported that TRIM28 S473 phosphorylation is mediated by different kinases, including p38MAPK, MSK1, RIPK3, ATM, MTORC1, and PKC-δ. The S473 phosphorylation event can be divided into two scenarios: context-dependent and normal condition. Regarding context-dependent phosphorylation, TRIM28-S473 was reported to be phosphorylated by ATM/CHK2 in DNA damage response, which attenuates its binding to the HP1 and inhibits its transcriptional repression of target genes (41). During muscle differentiation, MSK1-mediated phosphorylation of TRIM28 releases the corepressors from the scaffold, unleashing transcriptional activation by MyoD/Mef2 and their positive cofactors (24). Upon oxidative stress, p38MAPK was shown to mediate TRIM28-S473 phosphorylation and subsequently protect colorectal cancer cells from DNA damage (42). During necroptosis, active RIPK3-driven TRIM28-S473 phosphorylation triggers a remarkable reduction in TRIM28 chromatin binding at cytokine genes, which leads to increased *SOX9* transactivation at the same gene loci and cytokine hyper-production (43). Under the normal condition, PKC-δ phosphorylates TRIM28 at Ser473 to induce cell cycle genes at the S-phase using Hela cells (44). In bladder cancer cells, phosphorylation of TRIM28 through mTORC1 induces hTERT gene transcription to promote cancer cell growth (45). However, neither PKC-δ nor mTORC1 were shown to directly bind and phosphorylate TRIM28 in these studies, implying that other kinases may directly phosphorylate TRIM28 at S473 under the normal condition, though we cannot rule out the possibility that different kinases were employed to phosphorylate TRIM28 in a cell-type specific fashion. In our study, we first demonstrate that RSK1 directly binds to TRIM28 using GST pull–down assay, followed by in vitro kinase assay to support RSK1 directly phosphorylating TRIM28. Furthermore, knockdown of RSK1 diminishes pS473-TRIM28 under the normal condition (Figure 6). Taken together, multiple kinases act in concert to phosphorylate TRIM28 at S473 to orchestrate gene expression in response to different cellular contexts.

Through mediating Rb hypophosphorylation, CDK4/6 inhibitors show in vivo activity in both hormone-sensitive and castration-resistant prostate cancer (46, 47). Clinical development of CDK4/6 inhibitors in PCa is now emerging, and phase II studies (NCT02905318) are currently underway. However, approximately 15% of CRPC harbor biallelic deletion or loss-of-function alterations of *RB1* (13–15), which become insensitive to CDK4/6 inhibition. Loss of *RB1* function liberates “transactivating” E2Fs (E2F1, E2F2, and E2F3) which primarily transactivate target genes enrolled in cell cycle progression, DNA synthesis and replication, and DNA damage repair and apoptosis (48). Among those, many mitotic kinases are upregulated following E2F dysregulation, and inhibition of some of them, such as Aurora A and PLK1, has been indicated to be synthetically lethal with *RB1* inactivation (49–52). While much effort has been made to identify E2F target genes upregulated following *RB1* inactivation, to date, no small molecules specifically targeting any of the transactivating E2Fs have been described or tested in clinical trials. Our study reveals that RSK1-driven pS473-TRIM28 regulated *E2F1* expression independent of Rb status, which appears to be a plausible target to control E2F1 expression and the aforementioned activity in Rb-deficient CRPC. Despite TRIM28 not being druggable, pS473-TRIM28 can be targeted by RSK inhibition to block TRIM28 downstream of the E2F pathway (Figure 7). In addition, our data indicated that RSK but not CDK inhibition remains effective in suppressing Rb-deficient cancer growth (Figure 7), highlighting that RSK inhibition may be a targeted therapy to treat Rb-deficient CRPC.

BI-D1870 belongs to an ATP-competitive pan-RSK N-terminal kinase domain inhibitor with IC50s in the nanomolar range (53). However, a high dose of BI-D18710 results in off-target inhibition of Aurora B, MELK, and MST2 (54). LJH685 was developed in a screening effort to identify BI-D1870 derivatives with improved affinity and specificity for RSK, but the pharmacokinetic issues remained (34). The compound PMD-026 is the first RSK inhibitor undergoing clinical trial in the US in patients with metastatic and triple-negative breast cancer, and the trial is currently ongoing (NCT04115306). PMD-026 decreased growth of 22Rv1 tumors in vivo and sensitized the tumors to the androgen antagonist enzalutamide (55). The on-target action of PMD-026 was not reported in these xenograft studies. Overall, our study provides a much-needed scientific rationale to apply specific RSK inhibitors in the treatment of CRPC.

## Methods

### Sex as a biological variable.

Our study exclusively examined male mice because the disease modeled is only relevant in males.

### Cell lines and chemical reagents.

Human embryonic kidney cell line 293T and prostate cancer cell lines LNCaP, C4-2B, and 22Rv-1 were obtained from American Type Culture Collection (ATCC). The 293T cells were cultured in DMEM with 10% FBS and 1× penicillin streptomycin, and prostate cancer cells were cultured in RPMI1640 with 10% FBS and 1× penicillin streptomycin solution. All cell lines were authenticated (Genetica DNA Laboratories) and free of mycoplasma (Mycoalert detection kit, NC9719283, Thermo Fisher Scientific). Puromycin (P8833) and doxycycline (D9891) were from Sigma-Aldrich, and G418 (T6512), ulixertinib (T7005), BI-D1870 (T6171), palbociclib (T6239), and LJH685 (T6877) were from Targetmol.

### Antibodies.

Rabbit polyclonal to KAP1 (ab10483) and anti-GAPDH antibody [6C5] (ab8245) were from Abcam; P90RSK Rabbit pAb (RSK1) #A15718, phospho-RPS6KA1-T573 Rabbit pAb (p-T573 RSK) #AP1103, KAP1/TRIM28 #A2245 Rabbit pAb were from ABclonal; purified anti-TIF1-β (KAP-1, TRIM28) phospho (Ser473) #654102 was from Biolegend; Phospho-KAP-1 (Ser824) Antibody #4127, E2F-1 Antibody #3742, HA-Tag (C29F4) Rabbit mAb #3724, Myc-Tag (71D10) Rabbit mAb #2278, Cleaved Caspase-3 (Asp175) Antibody #9661, and Rb (4H1) Mouse mAb #9309 were from Cell Signaling Technology. ANTI-FLAG antibody produced in rabbit and Monoclonal ANTI-FLAG M2 antibody produced in mouse were from Sigma-Aldrich, and E2F1 Antibody (KH95), GST antibody (B-14) and α-tubulin antibody (B-7) were from Santa Cruz.

### DNA constructs, transfection, and lentiviral infection.

pCR8-TRIM28, HA-Flag TRIM28, and MYC-TRIM28 were used in a previous study. psPAX (12260), pMD2.G (12259), pCMVHA-E2F1 (24225), pGEX-5X-TRIM28 (45570), pDONR223-RSK1 (23860), and lentiCRISPRv2 puro (98290) were obtained from Addgene. pDONR223-RSK1 were transferred into gateway-compatible HA-Flag and pDEST-MYC vector by LR clonase (Invitrogen). TRIM28 1–410aa, 421–835aa, TRIM28-S473A, -S473D, -S824A, and -S824D mutants, and RSK1-CA, RSK1-KD mutants were generated by Q5 Site-Directed Mutagenesis Kit (NEB). pLKO.1 negative control and pLKO.1 shTRIM28 were used in a previous study. pLKO.1 shE2F1 (TRCN0000039659, TRCN0000000249, TRCN0000010327, and TRCN0000010328), shRb (TRCN0000288710, TRCN0000010419, and TRCN0000295892), shRSK1 (TRCN0000001385, TRCN0000001386, and TRCN0000001388), shRSK2 (TRCN0000196642, TRCN0000194851, and TRCN0000006354), shRSK3 (TRCN0000006354 and TRCN0000006355) and shRSK4 (TRCN0000314887 and TRCN0000196549) were purchased from Sigma-Aldrich. RB1 and TRIM28-gRNA was subcloned into lentiCRISPRv2 puro vector. To transiently overexpress genes, PCa cells were transfected with plasmid DNA mixed with X-tremeGENE HP DNA Transfection Reagent (Sigma-Aldrich). To generate lentiviral supernatant, lentiviral overexpression, shRNA, and lentiCRISPRv2 constructs were transfected along with psPAX and pMD2.G by PEI (23966, Polyscience) into 293T cells for 48 hours before the lentiviral supernatant was collected and filtered by 0.45 μM nitrocellulose syringe filter. Lentivirus transduction in prostate cancer cell lines was conducted under 2 μg/mL polybrene (H9268, Sigma-Aldrich) treatment. PCR primers are listed in Supplemental Table 1.

### RNA isolation, quantitative RT-PCR, and RNA sequencing.

Total RNA was isolated from cells with NucleoSpin RNA isolation kit (Takara). ReverTra Ace qPCR RT Master Mix kit (Toyobo) was used for RNA reverse transcription. qRT-qPCRs were performed using 2xBullseye EvaGreen qPCR MasterMix (MIDSCI) on Quantum 3 Real-Time PCR System (Applied Biosystems) and relative expression of mRNA was determined using GAPDH as the loading control. qRT-qPCRs data were obtained in triplicated experiments. PCR primers are listed in Supplemental Table 1. For RNA-seq, total RNA was isolated as described above. RNA-seq libraries were prepared from 0.5 μg high-quality DNA-free RNA using NEBNext Ultra RNA Library Prep Kit, according to the manufacturer’s instructions. The libraries passing quality control (equal size distribution between 250 and 400 bp, no adapter contamination peaks, no degradation peaks) were quantified using the Library Quantification Kit from Illumina (Kapa Biosystems, KK4603). Libraries were pooled to a final concentration of 10 nM and sequenced paired-end using the Illumina Nova-seq.

### Coimmunoprecipitation.

To probe the reciprocal interaction between ectopically-expressed TRIM28 and RSK1, 293T cells transfected with MYC-RSK1/TRIM28 along with empty vector or HA-Flag TRIM28/RSK1 were lysed in NETN buffer (100 mM NaCl, 20 mM Tris-Cl, pH 8.0, 1 mM EDTA, and 0.5% NP-40) supplemented with a complete protease inhibitor cocktail (Roche) at 4°C for 20 minutes. Lysates were incubated with 1 μg anti-Flag (M2) overnight at 4°C. Surebeads Protein G magnetic beads (Biorad), 10 μL per IP, were added the next day and incubated for 2 hours at 4°C. Bound proteins were eluted from the beads with 1.5× sample buffer for 15 minutes at 37°C, and the supernatant was transferred to a new tube to boil at 95°C for another 2 minutes before SDS-PAGE analysis.

### In vitro kinase assay.

GST-TRIM28 (2 μg fragment 1 and 2) were incubated with 100 ng recombinant active RSK1 (81398, active motif) in 1× kinase buffer (25 mM Tris-HCl pH 7.5, 5 mM β-glycerophosphate, 2 mM dithiothreitol [DTT], 0.1 mM Na_3_VO_4_, 10 mM MgCl_2_), plus 5 μCi (γ-32P) ATP (#NEG002A250UC, PerkinElmer) at 30°C for 30 minutes. The samples were resolved in SDS-PAGE gel and stained by Coomassie blue, followed by autoradiography. For Western blot, cold kinase assay was performed in which [γ -32P] ATP was replaced with normal ATP (#9804, Cell Signaling Technology).

### In vitro functional assays.

Cell proliferation assays were carried out using the WST-1 kit (ClontechA) according to the manufacturer’s instructions. For colony formation assay, 2,000–10,000 cells were seeded into a 6-well plate. After 10–14 days of incubation, cells were fixed with 4% paraformaldehyde for 15 minutes at room temperature, followed by staining with 0.025% crystal violet for 2 hours.

### CUT&RUN-seq.

For TRIM28 and E2F1 CUT&RUN, 10 μL Concanavalin A–coated beads were added to 0.5 × 10^6^ LNCaP/C4-2B cell suspension in wash buffer (20 mM HEPES pH 7.5, 150 mM NaCl, 0.5 mM spermidine) for 10 minutes at room temperature (RT). Cells were incubated with 1 μg rabbit anti-TRIM28 (Abcam) and rabbit anti-E2F1 (Cell Signaling Technology) in Dig-wash buffer (20 mM HEPES pH 7.5, 150 mM NaCl, 0.5 mM spermidine, and 0.1% digitonin) supplement with 2 mM EDTA and 0.1% BSA. After secondary antibody (1:100) and pA-MNase (1:400) incubation for 1 hour at 4°C, beads are washed twice with Dig-wash buffer followed by low-salt rinse buffer (20 mM M HEPES pH 7.5, 0.5 mM spermidine, and 0.1% digitonin), placed in an ice-water bath before the addition of 10 mM CaCl_2_ for 10 minutes. Digestion was stopped by addition of Stop buffer (170 mM NaCl, 20 mM EGTA, 4 mM EGTA, 0.1% digitonin, 50 μg/mL RNase A, and 25 μg/mL glycogen). Digested chromatin was released by incubating the samples in 37°C water for 30 minutes and separating from the beads by a magnetic stand. The supernatant was subjected to Proteinase K digestion at 50°C for 1 hour and PCI extraction. DNA libraries were prepared using NEBNext Ultra II DNA Library Prep Kit according to manufacturer instructions.

### ATAC-Seq.

ATAC-seq was performed as previously reported. First, 50,000 cells were collected, then washed once by 1×PBS and tagmented in 1×TD buffer with 2.5 μL Tn5 (diagenode), 0.01% digitonin (Sigma-Aldrich), and 0.3×PBS. The reactions were incubated at 37 °C for 30 minutes in a thermomixer with 300 rpm mixing. The tagmented DNA was purified using the DNA Clean & Concentrator-5 kit (ZYMO Research). Libraries were amplified and adapter dimers and primer dimers were cleaned up.

### Immunofluorescence.

Cells were grown on poly-D-lysine–coated coverslips and fixed with 4% paraformaldehyde for 15 minutes at room temperature, followed by permeabilization with 0.5% Triton X-100 for 15 minutes at room temperature. After 3 washes with PBS, cells were incubated with a blocking buffer (3% BSA in PBS) for 30 minutes. Then, cells were incubated with mouse anti-pS473-TRIM28 (1:500; Biolegend), and rabbit anti-TRIM28 (1:500; ABclonal) diluted in blocking buffer overnight at 4°C in a humidity chamber. This was followed by 3 washes and incubation with FITC (catalog 715-095-151) and TRITC (catalog 711-025-152)-conjugated secondary antibodies (1:500; Jackson ImmunoResearch) for 1 hour. DAPI was used to label nuclear DNA. After 3 washes with PBS, coverslips were mounted on glass slides in Prolong Gold antifade reagent (Invitrogen). The cells were imaged by Nikon Eclipse Ti2 fluorescent microscope system, and images were edited using Adobe PhotoShop (version 25.7.0).

### IHC.

IHC was performed on paraffin-embedded tissue sections. After deparaffinization, rehydration, and antigen retrieval with citrate buffer (Invitrogen), slides were permeabilized with 0.5% Triton X-100. Slides were then subjected to blocking and IHC staining using ImmPRESS HRP Universal PLUS Polymer Kit (Vector Laboratories) as described by the manufacturer. The following primary antibodies were used and incubated overnight at 4°C in a humidity chamber: anti-TRIM28 (1:200; ABclonal), anti-pS473-TRIM28 (1:100; Biolegend), anti-E2F1 (1:50; Santa Cruz), anti-cleaved caspase3 (1:200; Cell Signaling Technology). After incubating with DAB until reaching the desired stain intensity, slides were counterstained with H&E Stain Kit (Vector Laboratories), washed with running tap water, dehydrated in ethanol, cleared with xylene, and mounted with VectaMount AQ Aqueous Mounting Medium. Slides were visualized and imaged with a Nikon Upright 80I microscope system FL with color camera.

### Tissue microarray.

To evaluate the expression of TRIM28 in PCa, tissue microarrays (TMAs) of PCa specimens containing 154 prostate cancer cases, 30 normal prostate specimens, and 15 BPH specimens were obtained through the Markey Cancer Center Biospecimen Procurement and Translational Pathology Shared Resource Facility (Lexington, Kentucky, USA). The University of Kentucky Institutional Review Board approved the use of human prostate tissue. To evaluate the protein level of pS473-TRIM28 in primary PCa and CRPC, TMAs containing 30 cases of prostate adenocarcinoma were obtained through the Cooperative Human Tissue Network (CHTN), and TMAs containing human CRPC specimens were obtained from the University of Washington Medical Center Prostate Cancer Donor Program, which is approved by the University of Washington Institutional Review Board. UWTMA100 Array A-C were investigated, consisting of 1-mm–diameter core triplicates of visceral metastases and bone metastases (number of sites, 99), were collected from 30 patients. The intensity was scored as negative (score = 0), weak (score = 1), moderate (score = 2), or strong (score = 3), which was multiplied by the staining percentage to produce the product score for each core.

### Cell-line derived xenograft and Patient-derived xenograft Models.

22Rv1-*RB1*-KO cells (1 × 10^6^) were inoculated into castrated NSG mice and LuCaP 145.1 tumor were subcutaneously grafted into noncastrated 6-8–week old male NSG mice (Charles River Laboratory) respectively. To evaluate the effect of CDK or RSK inhibitor in Rb-deficient tumor progression, when tumor size reached an average of approximately 100 mm^3^, mice were randomly divided into 3 groups and treated with vehicle control, palbociclib (75 mg/kg daily; oral gavage), and BI-D1870 (50 mg/kg 3 times a week; intraperitoneal). Tumor size was measured twice a week, and tumor volumes were estimated using the formula (π/6) × L × W^2^), where L = the length of the tumor and W = the width. After the endpoint was reached, mice were euthanized, and tumors were excised and weighed. Tumor sections were formalin-fixed, paraffin-embedded, and then IHC stained with pS473-TRIM28, E2F1 and cleaved caspase-3.

### Organoid generation.

Minced *Pten^–/–^* PCa tissue were digested in 5 mg/mL collagenase type II (Life technologies, Cat.No.17101-015) in Advanced DMEM-F12 (Life technologies, Cat. No. 12634-34) supplemented with Y-27632 (Abmole Bioscience, Cat. No. M1817) for 1 hour at 37°C on a shaking incubator. Then, digested tissues were centrifuged at 200*g* for 5 minutes at 4°C and the resulting pellets were further digested by TrypLE Express (Life technologies, Cat. No. 12605-010), followed by resuspension in Advanced DMEM-F12. The cell suspensions were filtered through a 40-μm cell strainer to remove tissue debris and obtain single-cell suspensions. Cells were resuspended in growth factor-reduced Matrigel (BD, Cat.No.356231) at a ratio of 1,000 cells / 10 μL 50% Matrigel. Droplets were plated in the center of a 12-well culture plate with three droplets per well. The plate was then placed upside down in a 37°C incubator for 60 minutes to allow the Matrigel to solidify. Prewarmed Advanced DMEM-F12 supplemented with Y-27632 was added into each well. To knockdown Rb in organoids, 1 × 10^5^ organoids were resuspended in transduction medium (1× B27, 1× Glutamax, 10 mM HEPES, 100 μg/ml Primocin, and 10 μM Y27632 in AdDMEM/F12) and incubated with shRb lentivirus and 8 μg/mL Polybrene for 20 minutes at room temperature. After lentivirus infection, infected organoids were collected via centrifugation at 600*g* for 1 hour at room temperature. The supernatant was then removed and resulting pellets were resuspended in AdDMEM/F12 medium and placed at 37°C, in a 5% CO2 incubator for 3–3.5 hours to recover. Infected organoids were seeded into a 12-well culture plate at three droplets per well. Twenty-four hours after seeding, puromycin (1 μg/mL) was added to select shRb-infected organoids for 2 days. After 5–7 days, organoids were harvested to test the knockdown efficiency of Rb.

### Bioinformatics analysis.

For RNA-seq, sequencing reads were trimmed and filtered using Trimmomatic (V0.39) to remove adapters and low-quality bases. Trimmed reads were mapped to the human reference genome assembly GRCh37 (hg19) transcript annotation using RSEM. RSEM results for normalization and differential expression analysis were performed using the R package EdgeR. Significantly up/downregulated genes between groups were determined as fold change greater than or equal to 2 and q-value less than 0.05. The gene set enrichment analysis (GSEA) was performed using GSEA software and the gene sets in the Molecular Signature Database (MSigDB). For Cut&Run-seq, paired-end sequencing reads were trimmed using Trimmomatic (v0.39) to remove adapters and low-quality bases. Trimmed reads were mapped to the human reference genome assembly GRCh37 (hg19) using Bowtie2 (v2.3.5.1), allowing fragments up to 2 kb to be aligned. The alignment result was further filtered by SAMtools to remove reads unmapped, not primary alignment, reads failing platform, reads mapped to mitochondria, reads mapped to ENCODE blacklisted genomic regions, and multi-mapped reads. PCR duplicates were removed using Picard (v2.26.8). Peaks were called by MACS2 (v2.2.7.1). The coverage track bigwig files were generated with bin size 10 and were normalized by reads per genome coverage using deeptools (v3.5.1). Significantly differential accessible regions between groups were identified using the R package DiffBind (v3.15) (q-values less than 0.05 and fold changes greater than 2) based on consensus peaks.

### Statistics.

For q-PCR analysis, WST1 cell proliferation, colony assay, and IHC quantification, Student’s *t*-test (2-tailed, unpaired) was used to determine statistical significance. Error bars are presented as the mean ± SEM from triplicate samples. To compare tumor growth in xenograft assays, a one-way or two-way ANOVA test was performed to evaluate the statistical significance.

### Study approval.

All procedures involving mice were approved by the Institutional Animal Care and Use Committee at the University of Kentucky and complied with all relevant ethical regulations. The University of Kentucky Institutional Review Board and the University of Washington Institutional Review Board approved the use of human prostate tissue.

### Data availability.

High-throughput sequencing data are deposited in GEO dataset (GSE287229). Supporting data values can be found in Supporting data values file. All data are available in the main text or the supplemental materials, and material requests should be addressed to Ka-wing Fong (willfongkw@uky.edu).

## Author contributions

MK and KF conceived the project and designed the experiments. MK, YZ, RW, JG, DBA, and KF performed experiments. JL, RG, and CW conducted bioinformatics and statistical analysis. TG and XL provided critical discussions and conceptualization of the project. MK and KF generated the Figures and wrote the manuscript.

## Supplementary Material

Supplemental data

Unedited blot and gel images

Supporting data values

## Version 1 06/16/2025

Electronic publication
