# Supplementary material for: RSK1-driven TRIM28/E2F1 feedback loop promotes castration-resistant prostate cancer progression
Source: J Clin Invest. 2025 Jun 16;135(12):e185119. doi: 10.1172/JCI185119 (PMC12165809; doi:10.1172/JCI185119)
Supplement: Supplemental data [file jci-135-185119-s150.pdf]

RSK1-driven TRIM28/E2F1 feedback loop promotes castration-resistant prostate cancer progression

Miyeong Kim[1], Jinpeng Liu[2], Yanquan Zhang[1], Ruixin Wang[1], Ryan Goettl[2], Jennifer Grasso[1], Derek B. Allison[3], Chi Wang[2], Tianyan Gao[4], Xiaoqi Liu[1], Ka-Wing Fong[1,5*]

Content:

Supplementary Figure S1: TRIM28 is a novel E2F1-target in advanced prostate cancer

Supplementary Figure S2: TRIM28 regulates the E2F pathway in advanced prostate cancer

Supplementary Figure S3: TRIM28 regulates E2F1 genomic function

Supplementary Figure S4: pS473-TRIM28 promotes transcriptional activation of E2F1

Supplementary Figure S5: RSK1 kinase directly phosphorylates TRIM28 at S473

Supplementary Figure S6: pS473-TRIM28 promotes CRPC progression

Supplementary Figure S7: The oncogenic function of RSK1 is partially mediated by phosphorylated TRIM28 and E2F1.

Supplementary Figure S8: Exploitation of the RSK1-TRIM28-E2F1 axis as a vulnerability in Rb-deficient PCa

Supplementary Table S1: Primers used in this study.

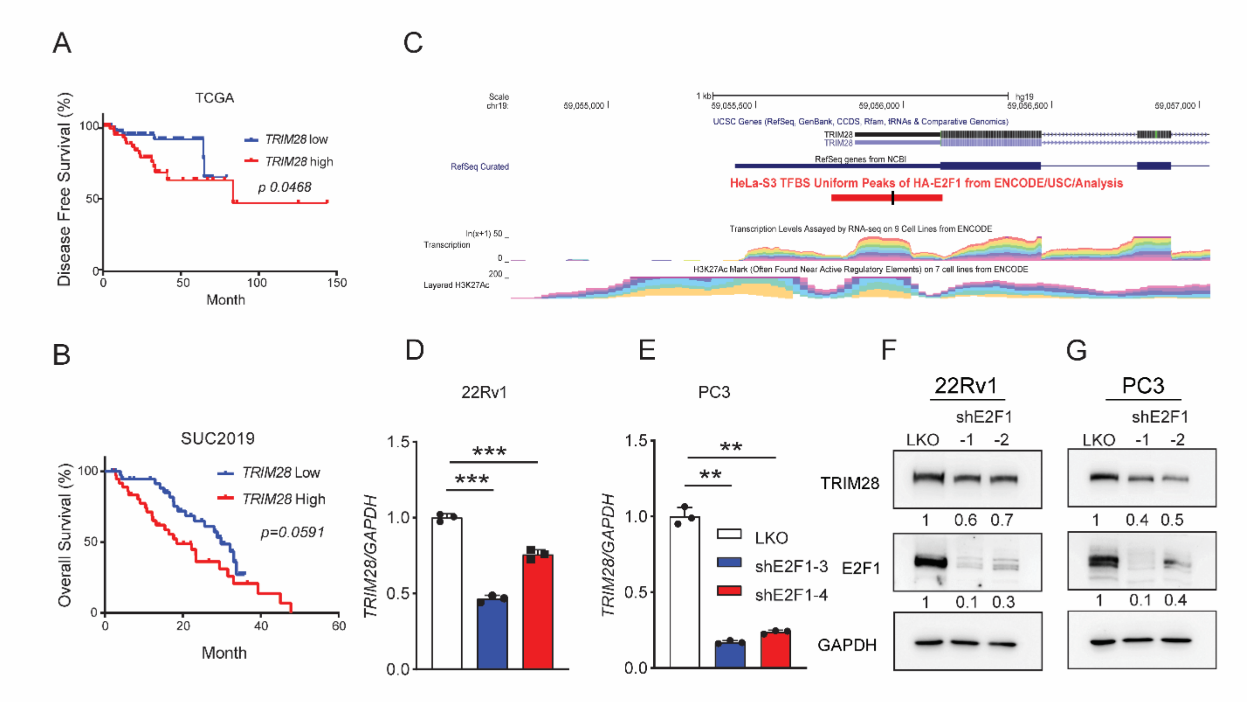

Supplementary Figure S1. (A-B) Kaplan-Meier plot depicts disease free survival of PCa patients stratified by *TRIM28*-high (1-SD above mean) and -low expression (1-SD below mean) (n=484) and overall survival of advanced PCa patients stratified by *TRIM28*-high (above median value) and -low expression (below median value) (n=76). Significant differences between groups was determined by one-way ANOVA. **(C)** Genome browser track depicts E2F1 binding-site (Red region) at the promoter of *TRIM28* loci. **(D-G)** 22Rv1 and PC3 cells were infected by 2 shRNAs targeting *E2F1*. RNA was harvested for qPCR analysis of *TRIM28* mRNA levels (D,E), while protein lysates were subjected to IB analysis against E2F1 and TRIM28 (F,G). The qPCR data are shown as mean ± SEM, n=3. Statistical analysis was performed using a two-tailed unpaired Student's t-test, with the Holm-Bonferroni method applied to correct for multiple comparisons. $^{**}p < 0.01$, $^{***}p < 0.001$. **(Related to Fig.1)**

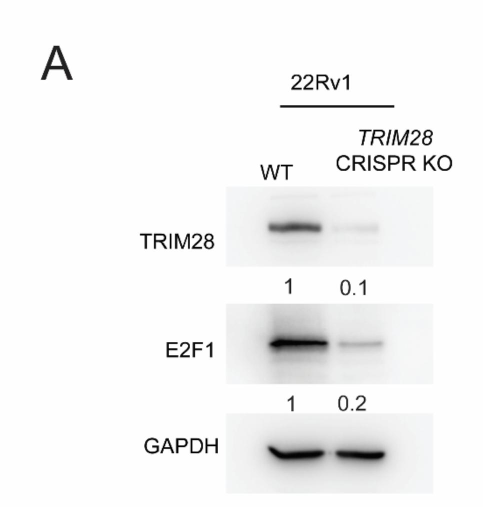

Supplementary Figure S2. (A) Protein lysate of 22Rv1 WT and *TRIM28* CRISPR KO cells were subjected to IB against TRIM28 and E2F1. **(Related to Fig.2)**

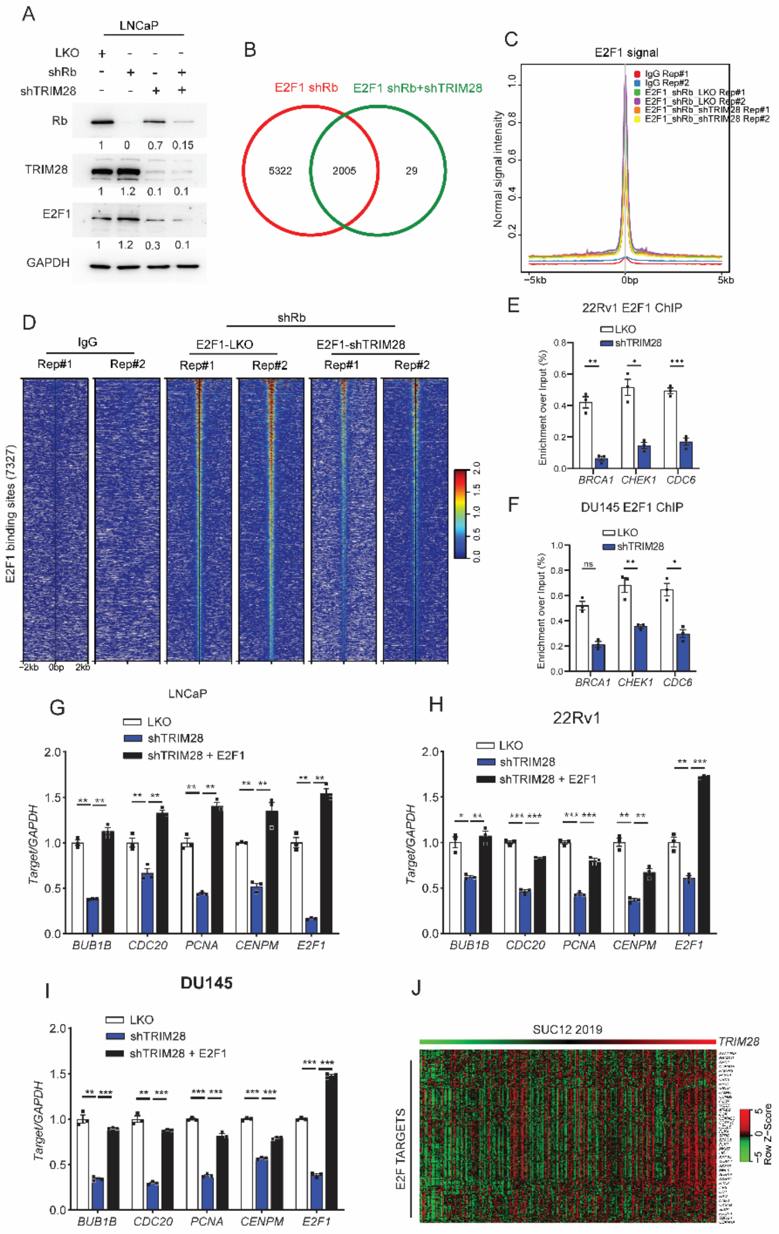

Supplementary Figure S3. (A-D) E2F1 CUT&RUN-seq was performed using LNCaP cells with shRb along with LKO and shTRIM28 (A). The E2F1 peak was called by MACS2, and the venn diagram indicates the overlapping of E2F1 binding sites for each treatment (B). The intensity plot depicts the ChIP-Seq peak intensity around peak center ±5 kb (C). The heatmaps indicate the E2F1 CUT&RUN-seq signal at E2F1 binding sites ±2 kb (D). **(E,F)** A subset of E2F targets were subjected to qPCR analysis of 22Rv1 and DU145 cells. The qPCR data are shown as mean ± SEM, n=3. **(G-I)** The qPCR analysis was performed in LNCaP (G), 22Rv1 (H) and DU145 (I) cells with the indicated treatment. The qPCR data are shown as mean ± SEM, n=3. Statistical analysis was performed using a two-tailed unpaired Student's t-test, with the Holm-Bonferroni method applied to correct for multiple comparisons. $^{**}p < 0.01$, $^{***}p < 0.001$. **(J)** Heatmap showing the levels of E2F-target genes in the CRPC samples from SUC12 2019 study (N=266) sorted by *TRIM28*. **(Related to Fig.3)**

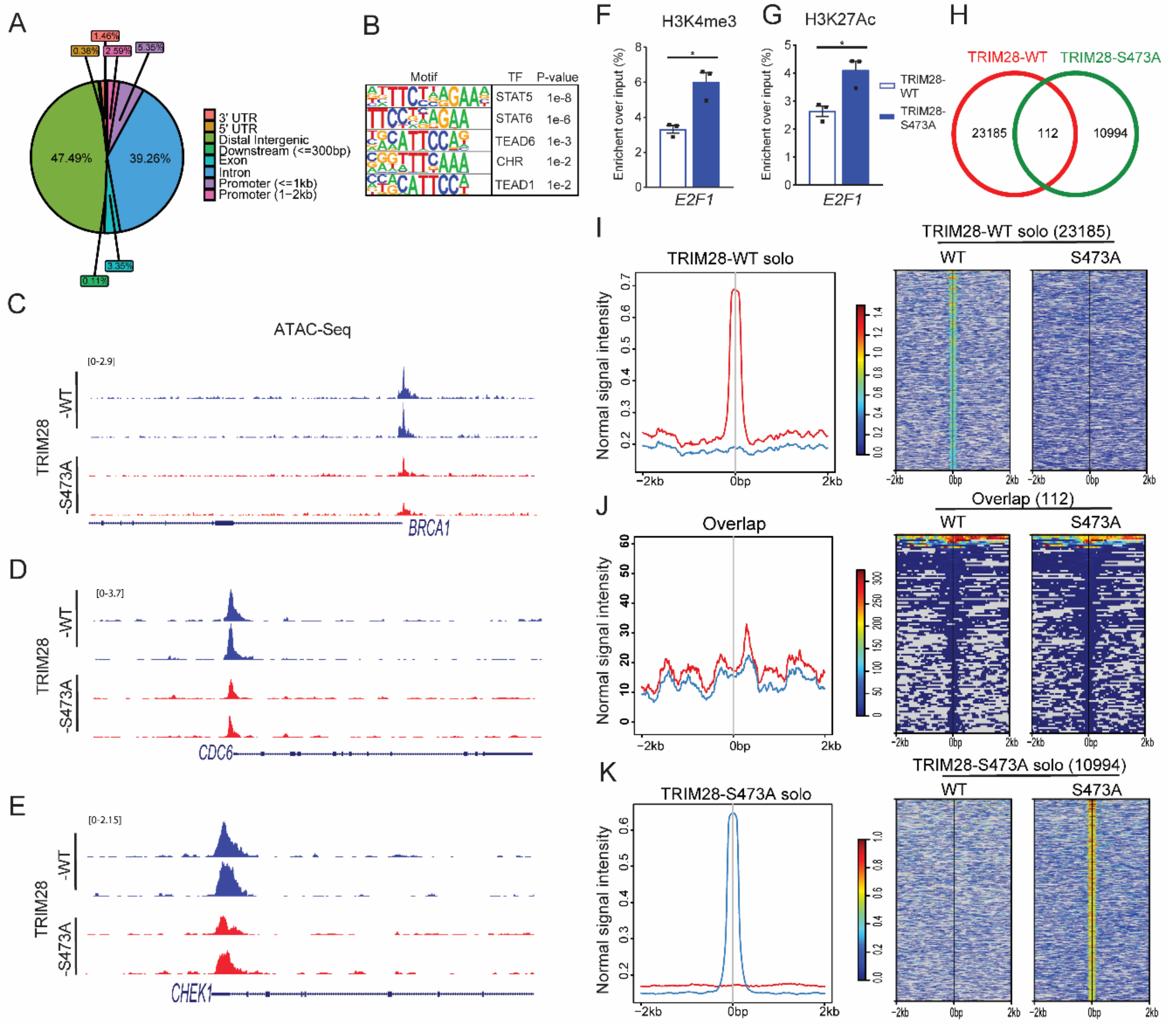

Supplementary Figure S4. (A) Pie chart depicting the genome distribution of IgG binding sites. (B) Transcription factor motif analysis of TRIM28 binding sites was performed by using HOMER. **(C-E)** The ATAC-seq genome browser tracks show representative E2F gene targets in C4-2B cells receiving indicated treatment. **(F,G)** pS473-TRIM28 imposes chromatin remodeling. H3K4me3 **(F)**, and H3K27Ac **(G)** ChIP were conducted using shTRIM28-treated C4-2B cells rescued by TRIM28-S473A, and TRIM28-WT. qPCR analysis targeting the *E2F1* gene was performed and normalized to input. The qPCR data are shown as mean ± SEM, n=3. Two-tailed unpaired Student's *t*-test, $*p < 0.05$, **(H-K)** TRIM28 CUT&RUN-seq was performed using C4-2B with *TRIM28*-KD rescued by lenti-HAF TRIM28-WT and the S473A mutant. The TRIM28 peak was called by MACS2, and the venn diagram indicates the overlapping of TRIM28 binding sites for each treatment (H). The intensity plot and heatmap depict the CUT&RUN-seq peak intensity at TRIM28-WT solo, S473A-solo and overlapping binding sites around peak center ±2 kb (I-K). **(Related to Fig.4)**

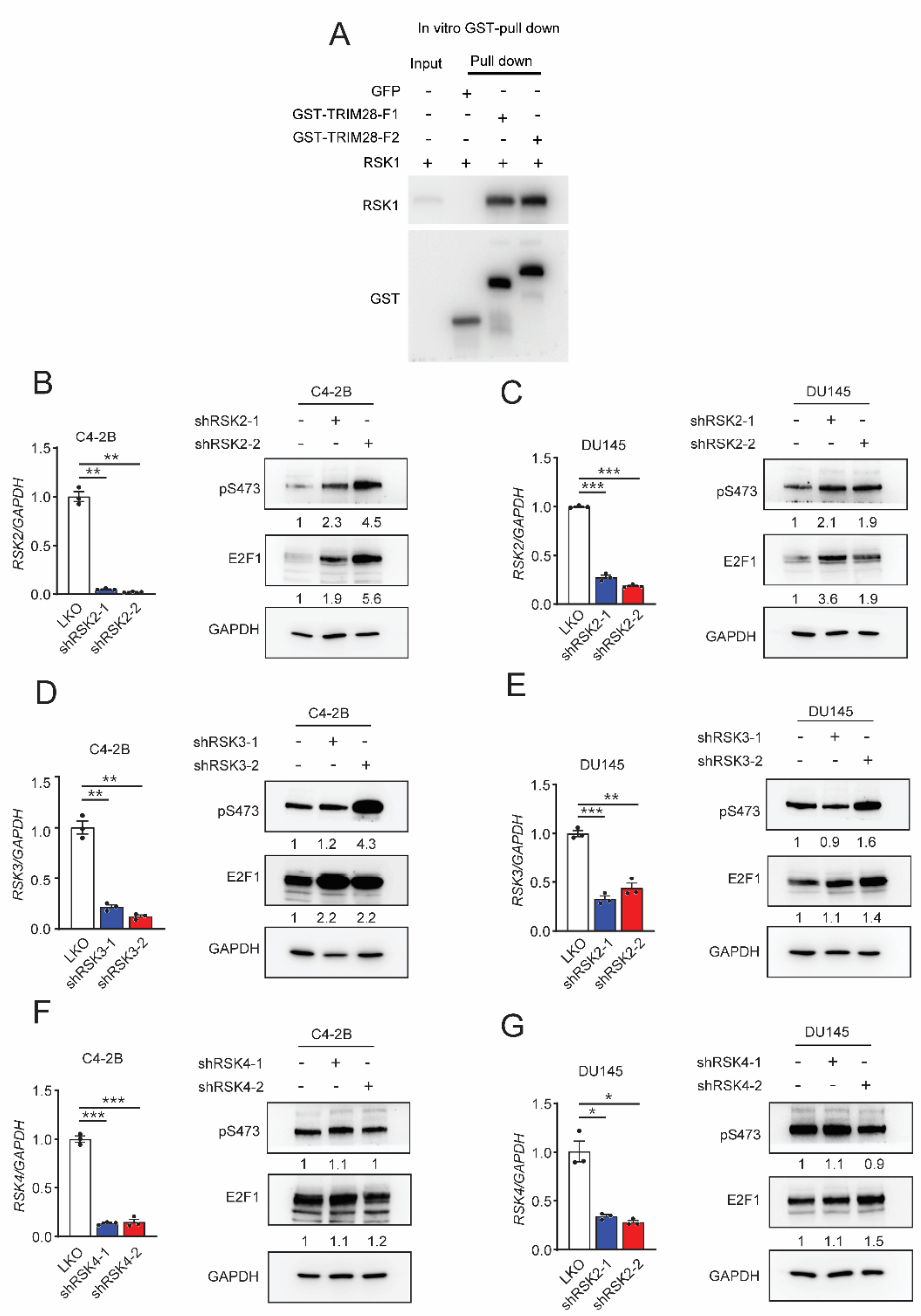

Supplementary Figure S5. (A) GST-pulldown assay was performed using *E.coli*-expressed GST-TRIM28 fragments (F1-F2) along with RSK1 protein. **(B-G)** RNA and protein lysates were harvested from C42B and DU145 cells infected by lentiviral supernatant containing LKO and 2-independent shRNA targeting *RSK2*-*RSK4* for 4 days, followed by qPCR analysis for *RSK2*-*RSK4* and IB against pS473, E2F1 and GAPDH. The qPCR data are shown as mean ± SEM, n=3. Statistical analysis was performed using a two-tailed unpaired Student's t-test, with the Holm-Bonferroni method applied to correct for multiple comparisons. $*p < 0.05$, $**p < 0.01$, $***p < 0.001$. **(Related to Fig.5)**

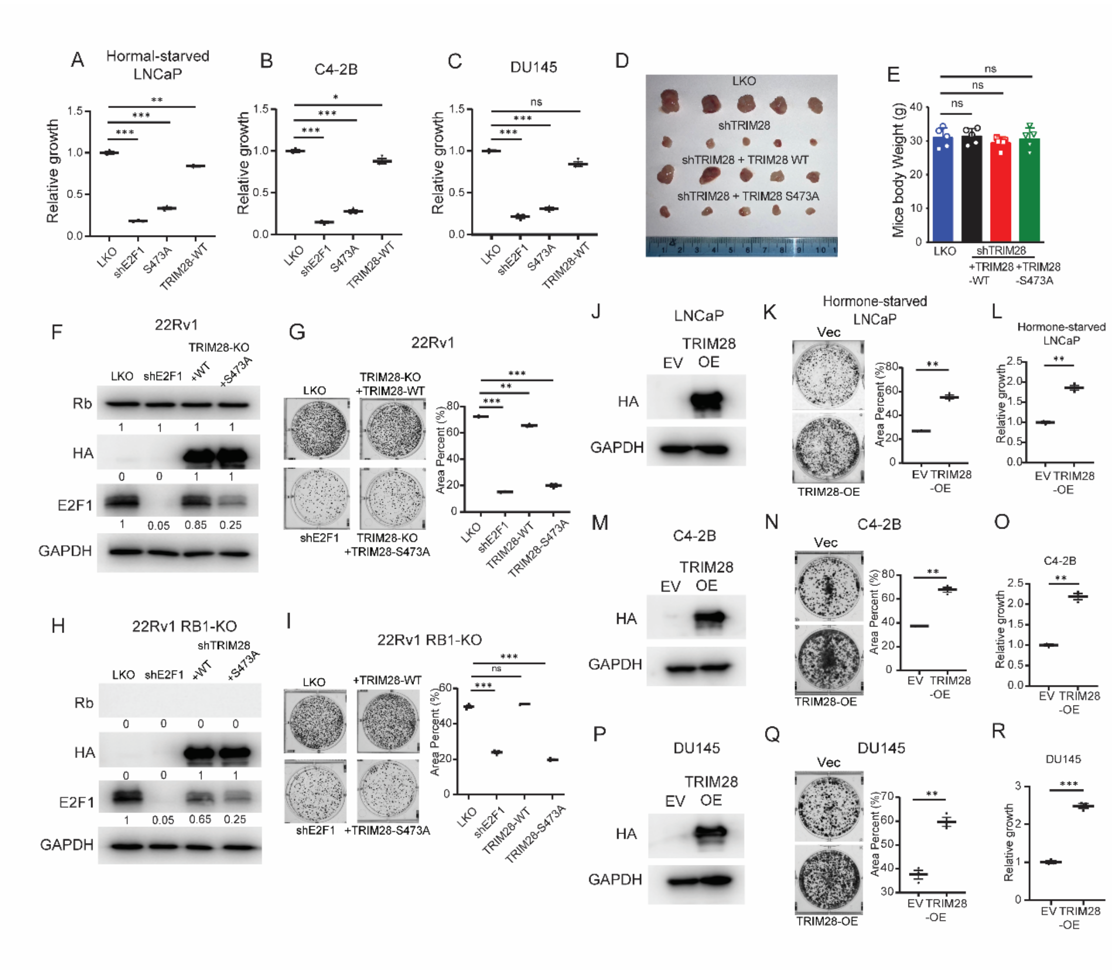

Supplementary Figure S6. (A-C) 2.5-8x10^3 cells with the indicated treatment were passed into a 96-well plate for 5 days and then analyzed using the WST-1 proliferation assay (A-C). Y-axis: Relative growth to control group. Data are shown as mean ± SEM, n=3. Statistical analysis was performed using a two-tailed unpaired Student's t-test, with the Holm-Bonferroni method applied to correct for multiple comparisons. *p < 0.05, **p < 0.01, ***p < 0.001. ns: not significant. **(D,E)** Images and mice body weight of xenograft experiment from Figure 6G. **(F-I)** 22Rv1-WT **(F,G)** and *RB1*-KO **(H,I)** cells receiving the indicated treatment were analyzed by colony formation assay. Quantification was conducted by image J (colony area plugin) and presented as mean ± SEM, n=3. Statistical analysis was performed using a two-tailed unpaired Student's t-test, with the Holm-Bonferroni method applied to correct for multiple comparisons. **p < 0.01, ***p < 0.001. ns: not significant. **(J-R)** Hormone-starved LNCaP, C4-2B and DU145 cells expressing empty vector or HA-Flag-tagged TRIM28 were subjected to IB (J, M, P), the colony formation assay (K, N, Q) and WST-1 proliferation assay (L, O, R), respectively. Data are shown as mean ± SEM, n=3. Two-tailed unpaired Student's *t*-test, **p < 0.01, ***p < 0.001. ns: not significant. **(Related to Fig.6)**

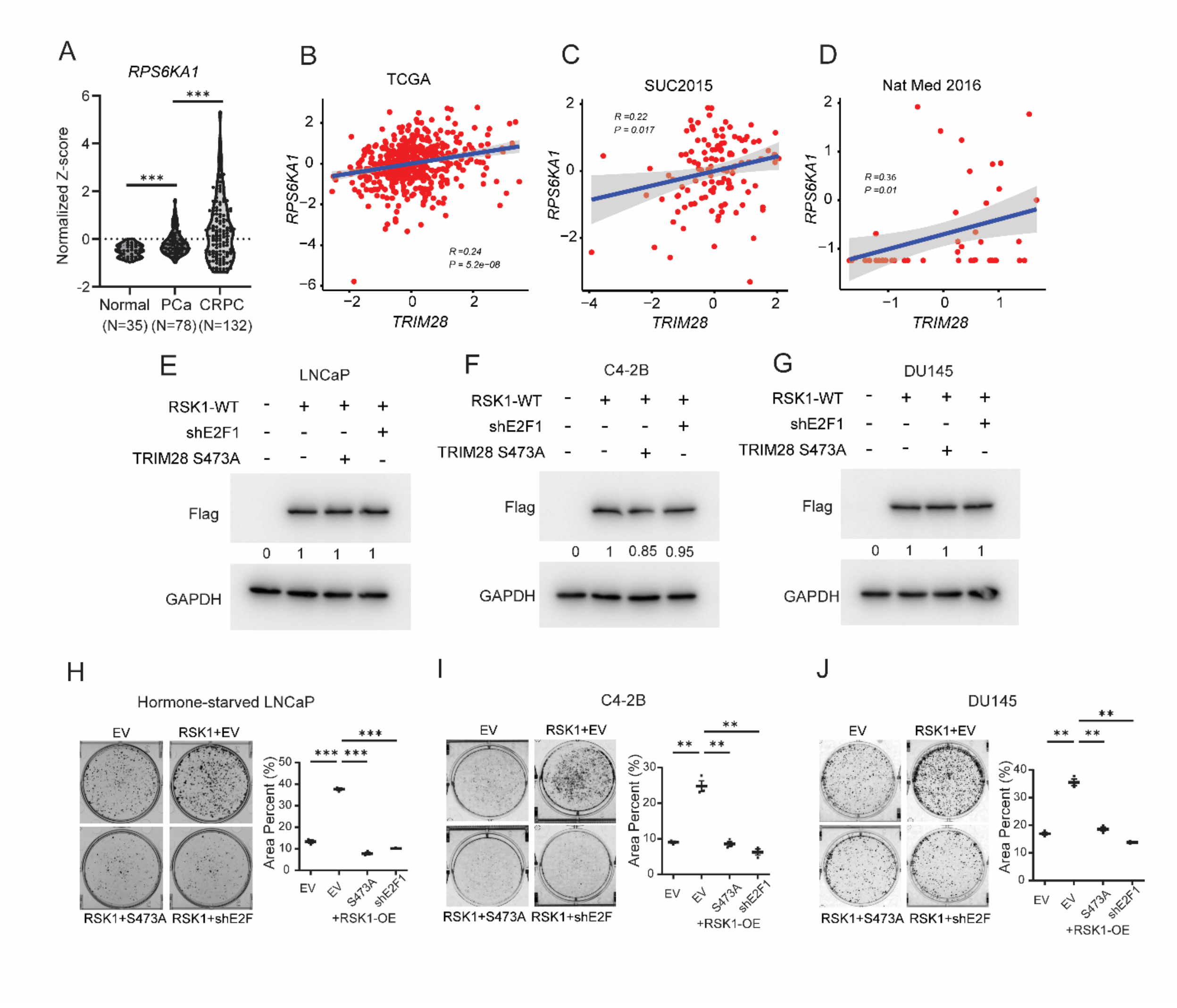

Supplementary Figure S7. (A) *RPS6KA1* (*RSK1*) RNA expression in PCa patient cohort (DbGAP). **(B-D)** The correlation of *TRIM28* and *RPS6KA1* (*RSK1*) expression in 3 patient cohorts. TCGA (n=491). SUC2015 (n=118). Nat Med 2016 (n=49). Statistical analysis is based on linear regression. **(E-J)** Hormone-starved LNCaP, C4-2B and DU145 cells with the indicated treatment were harvested for IB (E-G) and subjected to the colony formation assay for 7-14 days (H-J). Quantification was conducted by image J (colony area plugin) and presented as mean ± SEM, n=3. Statistical analysis was performed using a two-tailed unpaired Student's t-test, with the Holm-Bonferroni method applied to correct for multiple comparisons. $^{**}p < 0.01$, $^{***}p < 0.001$. **(Related to Fig.6)**

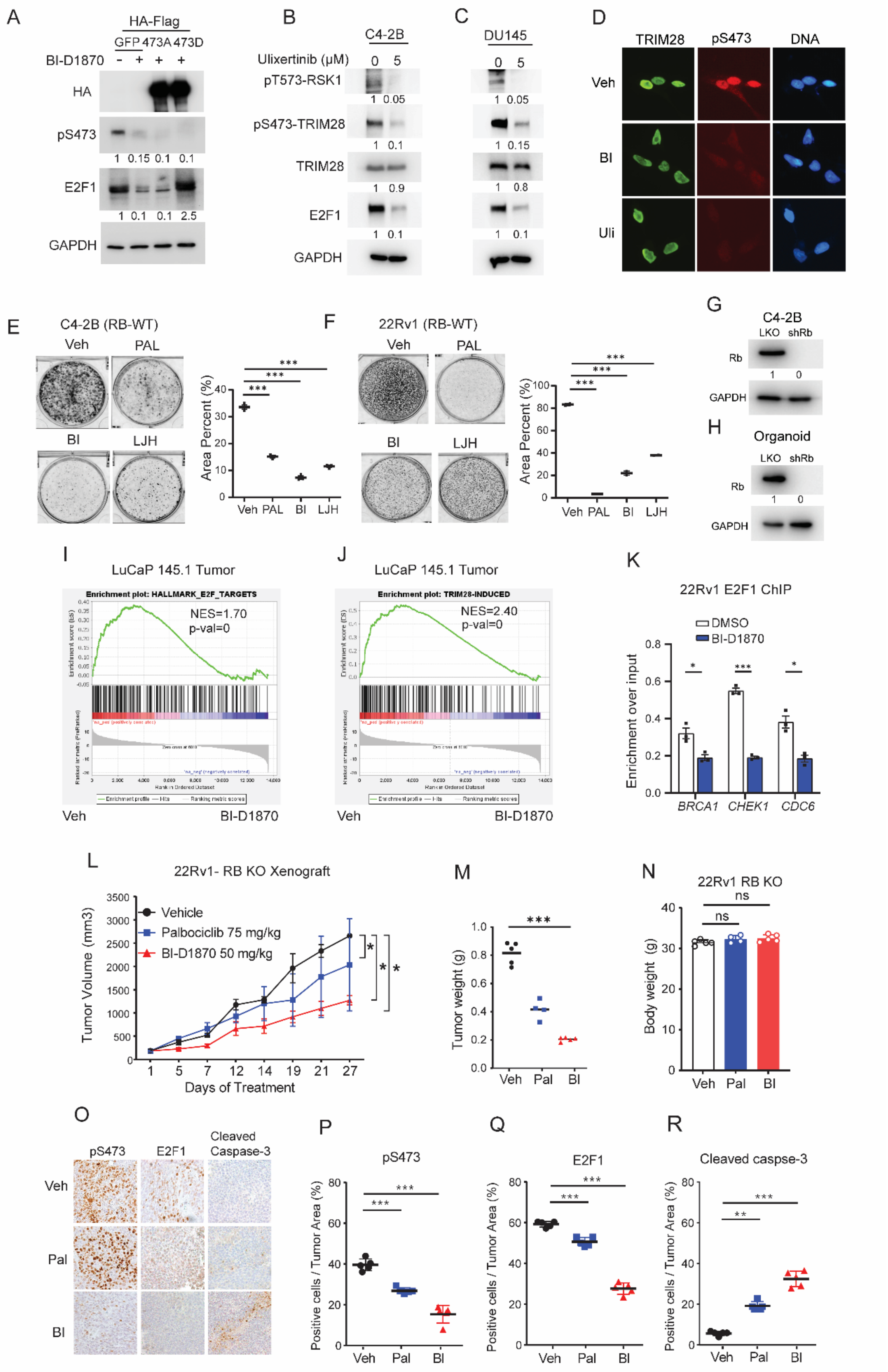
A
HA-Flag
GFP 473A 473D
BI-D1870
HA
pS473
1 0.15 0.1 0.1
E2F1
1 0.1 0.1 2.5
GAPDH
B
C4-2B
Ulixertinib (μM) 0 5
pT573-RSK1
1 0.05
pS473-TRIM28
1 0.1
TRIM28
1 0.9
E2F1
1 0.1
GAPDH
C
DU145
0 5
1 0.05
1 0.15
1 0.8
1 0.1
D
TRIM28 pS473 DNA
Veh
BI
Uli
E
C4-2B (RB-WT)
Veh PAL
BI LJH
Area Percent (%)
Veh PAL BI LJH
F
22Rv1 (RB-WT)
Veh PAL
BI LJH
Area Percent (%)
Veh PAL BI LJH
G
C4-2B
LKO shRb
Rb
1 0
GAPDH
H
Organoid
LKO shRb
Rb
1 0
GAPDH
I
LuCaP 145.1 Tumor
Enrichment plot: HALLMARK_E2F_TARGETS
NES=1.70
p-val=0
Veh BI-D1870
J
LuCaP 145.1 Tumor
Enrichment plot: TRIM28-INDUCED
NES=2.40
p-val=0
Veh BI-D1870
K
22Rv1 E2F1 ChIP
DMSO
BI-D1870
Enrichment over input
BRCA1 CHEK1 CDC6
L
22Rv1- RB KO Xenograft
Vehicle
Palbociclib 75 mg/kg
BI-D1870 50 mg/kg
Tumor Volume (mm3)
Days of Treatment
M
Tumor weight (g)
Veh Pal BI
N
22Rv1 RB KO
ns
ns
Body weight (g)
Veh Pal BI
O
pS473 E2F1 Cleaved Caspase-3
Veh
Pal
BI
P
pS473
Positive cells / Tumor Area (%)
Veh Pal BI
Q
E2F1
Positive cells / Tumor Area (%)
Veh Pal BI
R
Cleaved caspse-3
Positive cells / Tumor Area (%)
Veh Pal BI

Supplementary Figure S8. (A) C4-2B stably expressing indicated proteins were treated with Veh or BI-D1870 before being harvested for IB. **(B-C)** C4-2B **(B)** and DU145 **(C)** cells treated with Veh and ERK inhibitor (Ulixertinib) were harvested for IB against p-RSK, pS473-TRIM28, and E2F1. **(D)** Either BI-D1870 and Ulixertinib treatment diminishes pS473-TRIM28 immunostaining signal in C4-2B. **(E-F)** RB1-WT C4-2B or 22Rv1 cells treated by Veh, Palbociclib (500nM), BI-D1870 (1μM), LJH685 (5μM) were subjected to a colony formation assay. Quantification was conducted by image J (colony area plugin) and presented as mean ± SEM, n=3. Statistical analysis was performed using a two-tailed unpaired Student's t-test, with the Holm-Bonferroni method applied to correct for multiple comparisons. ***$p < 0.001$. **(G,H)** IB were performed to validate the Rb-KD in C4-2B cells and organoids. **(I,J)** RNA-seq analyses of LuCaP145.1 tumor. GSEA analysis showing that E2F pathway (I) and TRIM28-induced geneset (J) were significantly inhibited by BI-D1870 treatment. **(K)** E2F1 ChIP-PCR was performed using 22Rv1 cells treated with DMSO and 10μM BI-D1870 for 2 days. **(L-R)** NGS mice were implanted subcutaneously with 22Rv1-*RB1*-KO tumors, followed by treatment with vehicle, palbociclib (75 mg/kg) and BI-D1870 (50 mg/kg) for 3 weeks. Tumor sizes were plotted against days of treatment (L) and the data were analyzed by two-way ANOVA followed by the Tukey-corrected multiple-comparison test (mean ± SEM, n=5, *$p < 0.05$). Tumor weight was presented as a boxplot (M) and the toxicity was evaluated by mice body weight (N). At the endpoint, paraffin-embedded tumor tissues were subjected to IHC assay (O), followed by quantification (P-R). Data are shown as mean ± SEM, n=3. Statistical analysis was performed using a two-tailed unpaired Student's t-test, with the Holm-Bonferroni method applied to correct for multiple comparisons. **$p < 0.01$, ***$p < 0.001$. ns: not significant. **(Related to Fig.7)**

Supplementary Table S1: Primers used in this study.

qPCR primers	Sequence
E2F1 F	ACGCTATGAGACCTCACTGAA
E2F1 R	TCCTGGGTCAACCCCTCAAG
E2F2 F	CGTCCCTGAGTTCCCAACC
E2F2 R	GCGAAGTGTCATACCGAGTCTT
E2F3 F	TCCCTAAACCCGCTTCCAAAG
E2F3 R	AGTCTGCTGTAAGAGGTTGGC
GAPDH F	TGCACCACCAACTGCTTAGC
GAPDH R	GGCATGGACTGTGGTCATGAG
RSK2 F	AGGGCAGGGATCATTTGGAAA
RSK2 R	GGCCTTCTTCAATACCTTCATGG
RSK3 F	GAAGAAGGCGTCGTGAAGGAG
RSK3 R	CCGAACTTTTAGGGTGGCTTT
RSK4 F	GCCCTGGGACCGAGAAATG
RSK4 R	TGAGCAACTCAAACTGTGCAG
TRIM28 F	TGAGACCTGTGTAGAGGCG
TRIM28 R	CGTTCACCATCCCGAGACTT
TRIM28 F	CGGGATGGTGAACGTACTGTC
TRIM28 R	GTCTCGGCAGGTGAGAGTATC
BUB1B F	AAATGACCCTCTGGATGTTTGG
BUB1B R	GCATAAACGCCCTAATTTAAGCC
CDC20 F	GCACAGTTCGCGTTCGAGA
CDC20 R	CTGGATTTGCCAGGAGTTCGG
PCNA F	CCTGCTGGGATATTAGCTCCA
PCNA R	CAGCGGTAGGTGTCGAAGC
CENPM F	GCGGACTCGATGCTCAAAGA
CENPM R	TTCTGGAGACTGTATTTGCTGTG
**ChIP-PCR primers**	**Sequence**
E2F1 pF	GGCTGTGGAACTGATGATCC
E2F1 pR	CACGTGACCCTCAACCTGTA
TRIM28 pF	TTCTGTGTGGTCTGGAGGTG
TRIM28 pR	GTGGGGTATGCGAACCTTTA
BRCA1 pF	AAGCCGCAACTGGAAGAGTA
BRCA1 pR	CCGGATGACGTAAAAGGAAA
CHEK1 pF	TGGAGGAATGGTACCAGGAG
CHEK1 pR	GGACTCTTCCTTGGGGTTCT
CDC6 pF	CAGTTTGTTCAGGGGCTTGT
CDC6 pR	TCTTTCCACCTCCTCAGTGC

**Cloning primers**	**Sequence**
CA-RSK1 (Y702A) cF	GGCTGCCACGgccTCCGCACTCA
CA-RSK1 (Y702A) cR	ATGGCTCCCTTCACAAGC
KD-RSK1 (K447R) cF	GTATGCTGTCgcgGTCATTGATAAGAGCAAG
KD-RSK1 (K447R) cR	TCCATGTTGGTGGCCTTG
KD-RSK1 (K94R) cF	GTATGCTATGgcgGTGCTGAAGAAGGCAACG
KD-RSK1 (K94R) cR	AGGTGCCCACTGTCAGGC
pGEX TRIM28 1-420aa cF	TGAGAATTCCCGGGTCGA
pGEX TRIM28 1-420aa cR	AGGGCCTGTTGAGTTAGTG
pGEX TRIM28 421-835aa cF	GCACCCATGGCCCCTCCA
pGEX TRIM28 421-835aa cR	CATAGATCCACGACCTTCGATCAGATCC
TRIM28-luc cF 70	GGTACCGAGCTCTTACGCGT CGTTGGCCAGGCTGCTCTCGA
TRIM28-luc cR 70	CGAGCCCGGGCTAGCACGCGT CTGTCCGGGCCCCCATCCTC
RB1-gRNA cF	CACCGATAGGCTAGCCGATACACTG
RB1-gRNA cR	AAACCAGTGTATCGGCTAGCCTATC
TRIM28 gRNA cF1	CACCG ACGTTCACCATCCCGAGACT
TRIM28 gRNA cR1	AAAC AGTCTCGGGATGGTGAACGT C
TRIM28 gRNA cF2	CACCG TCTGTGTGAGACCTGTGTAG
TRIM28 gRNA cR2	AAAC CTACACAGGTCTCACACAGA C
