# Supplementary material for: RSK1-driven TRIM28/E2F1 feedback loop promotes castration-resistant prostate cancer progression
Source: J Clin Invest. 2025 Jun 16;135(12):e185119. doi: 10.1172/JCI185119 (PMC12165809; doi:10.1172/JCI185119)

Full unedited blot for

Figure 1J

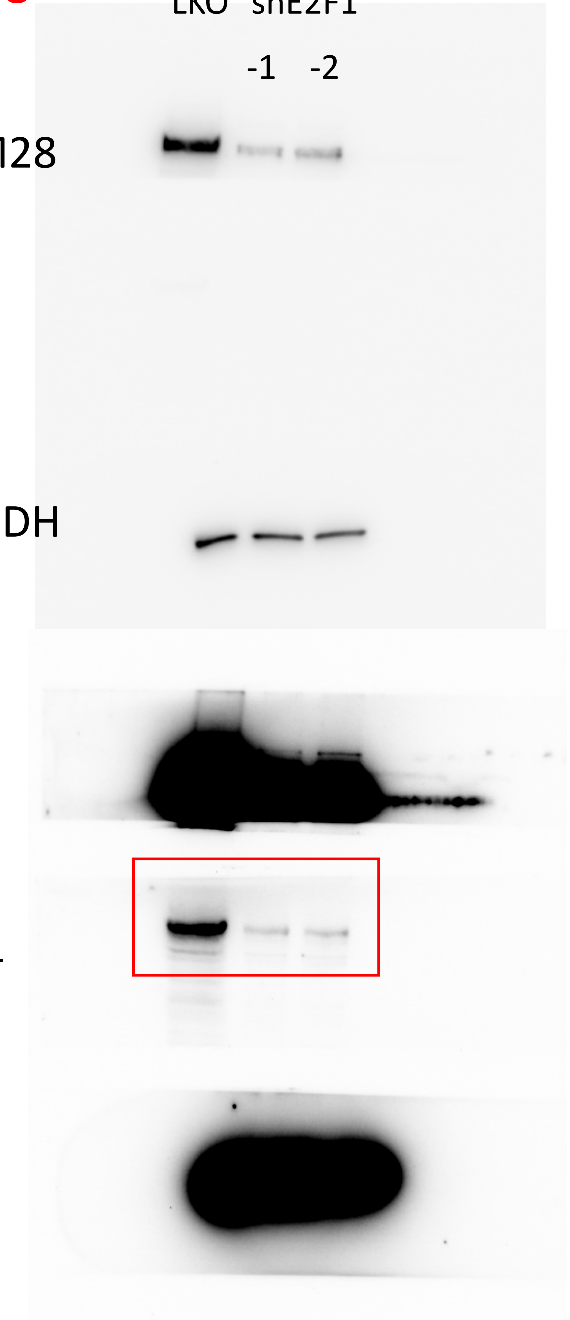

Figure 1K

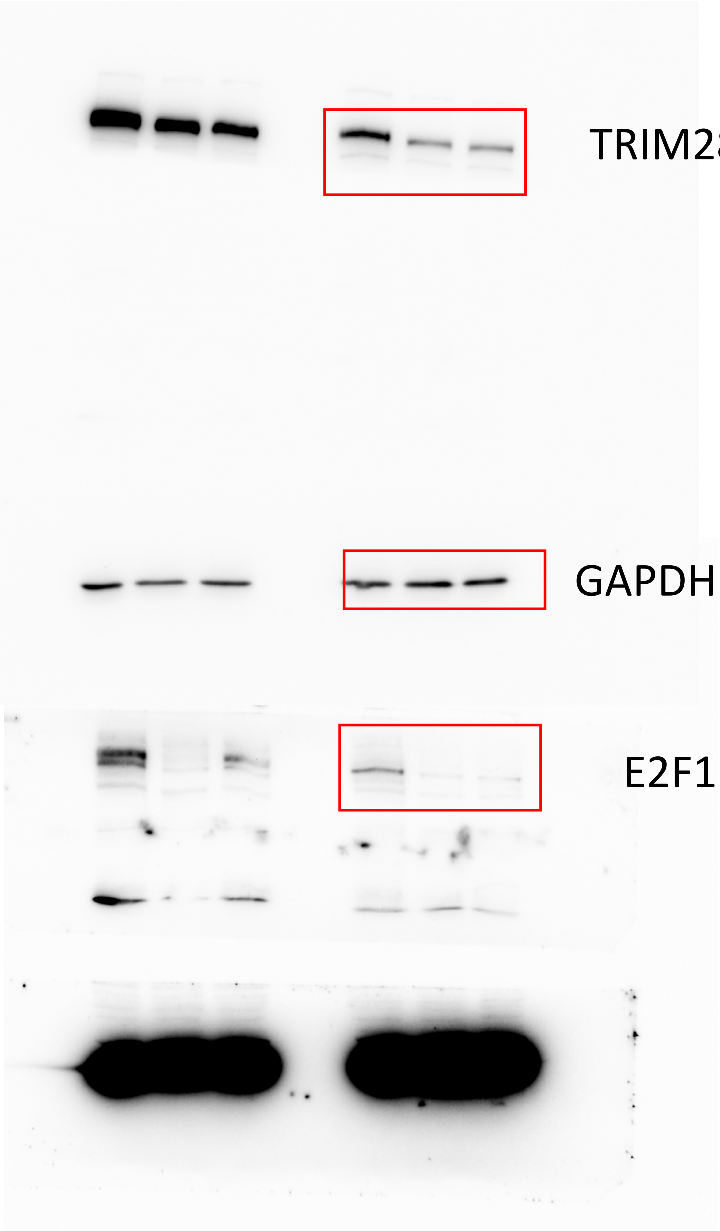

Figure 1M

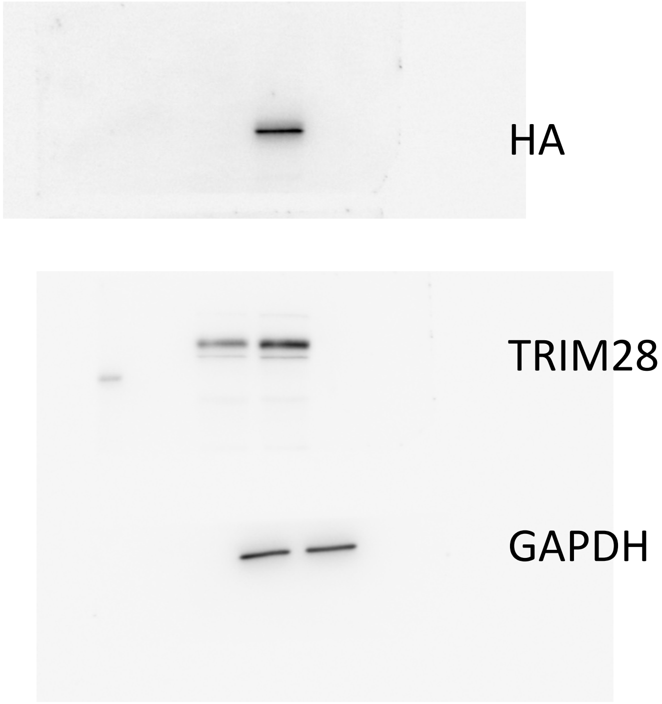

Full unedited blot for

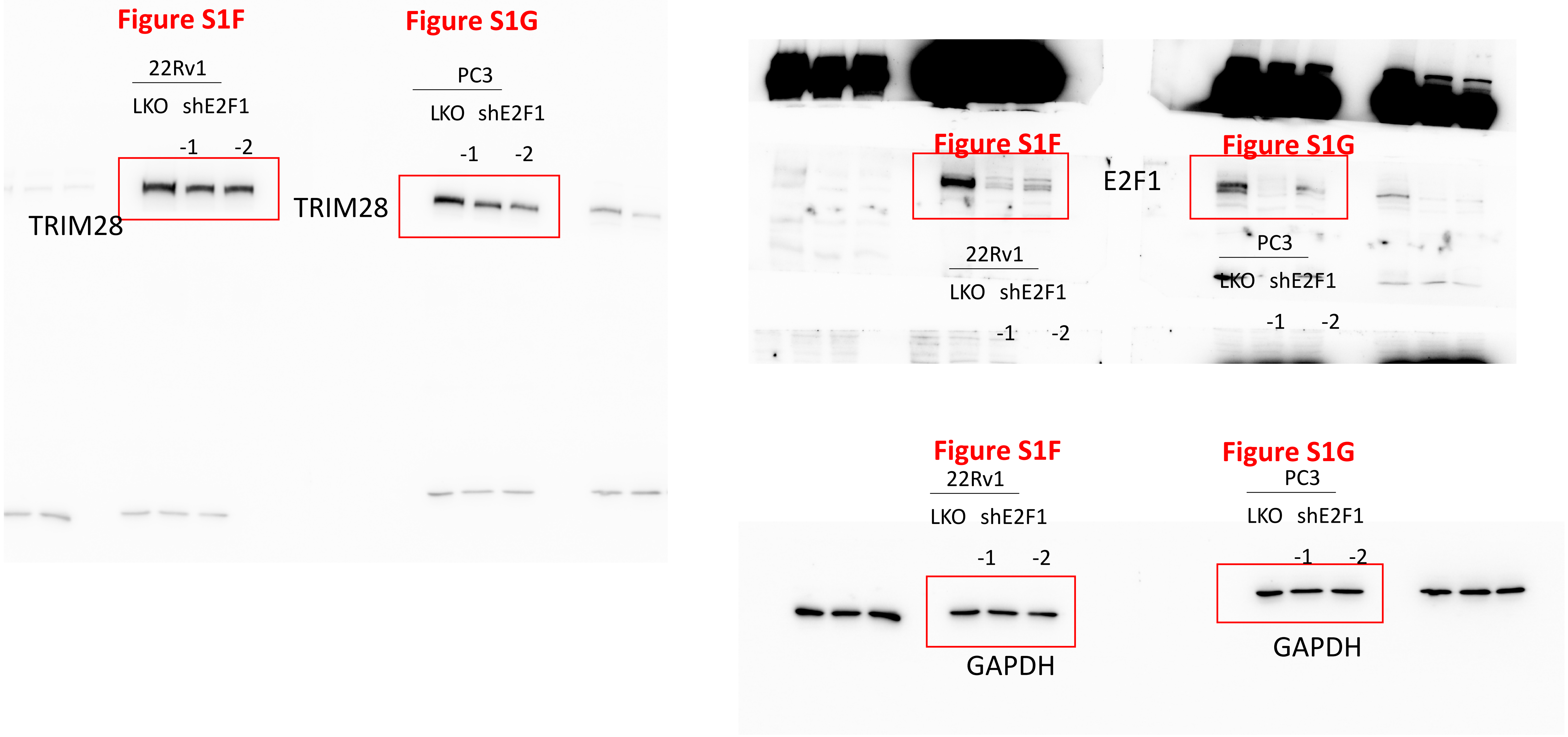

Full unedited blot for

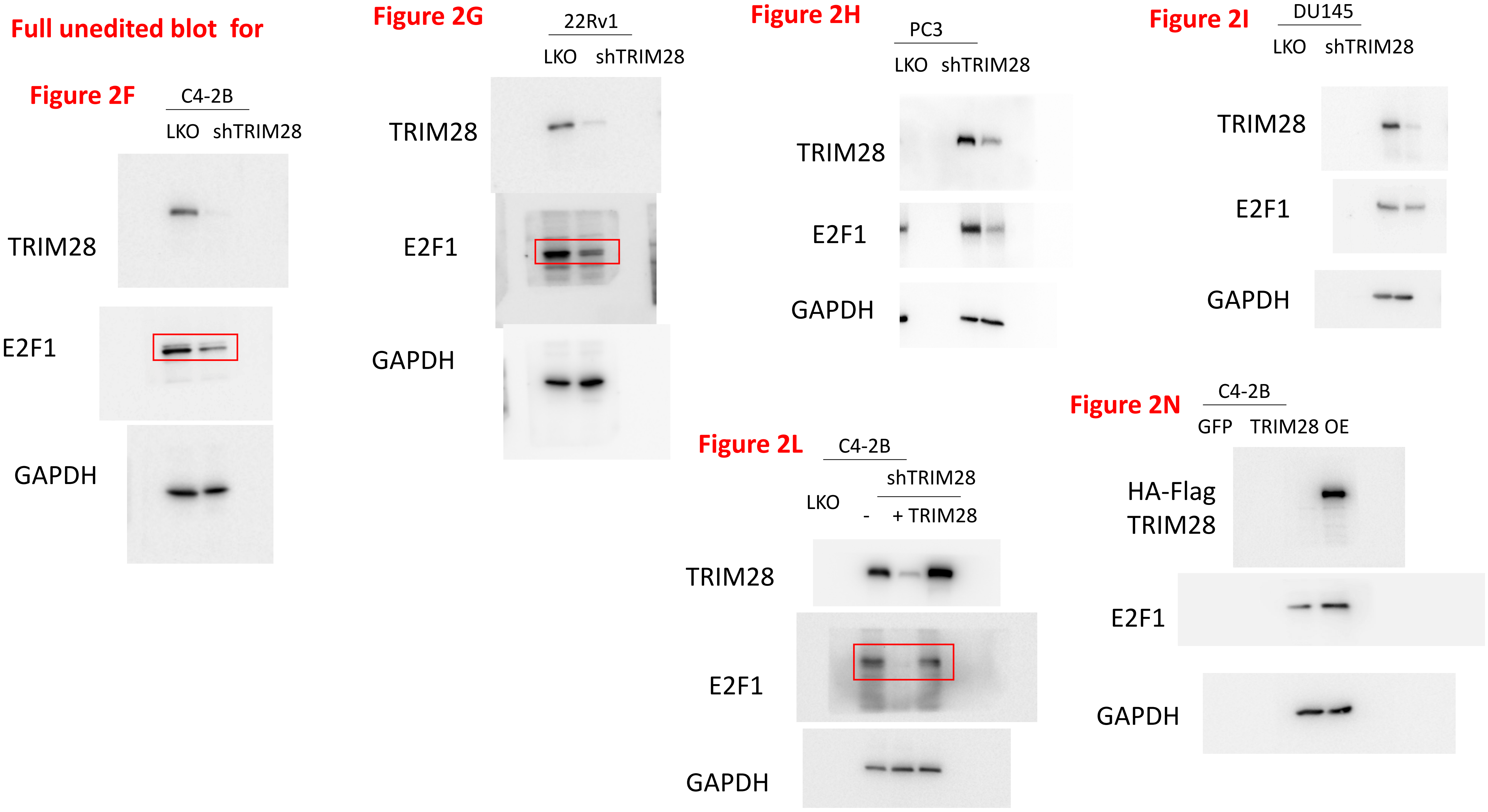

Full unedited blot for

Figure S2

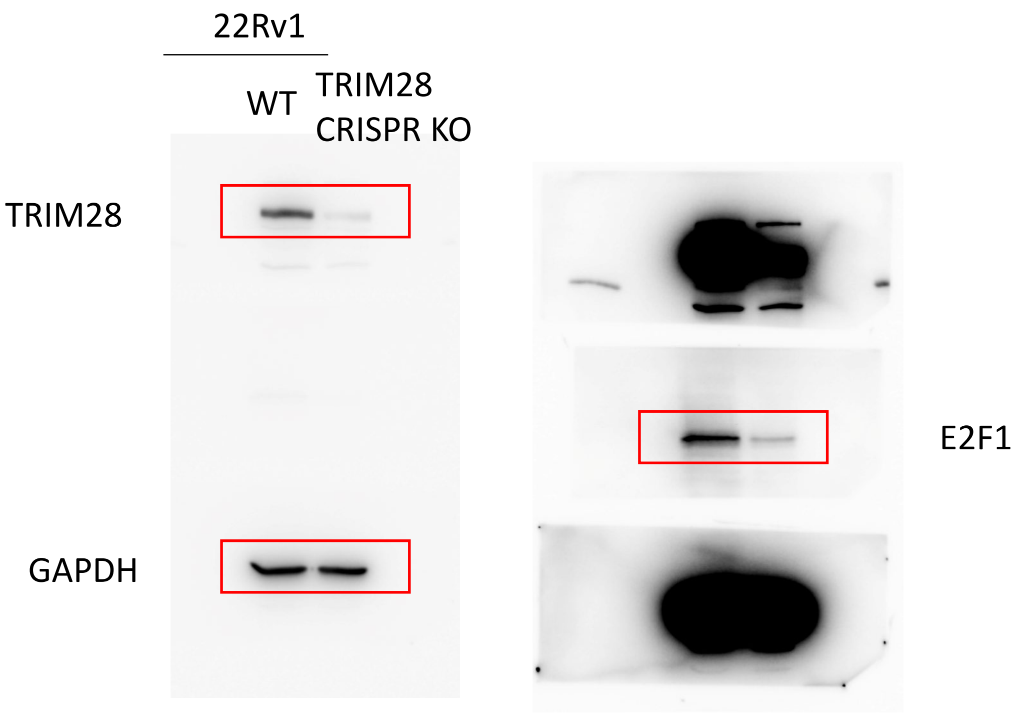

Figure 3E

Full unedited blot for

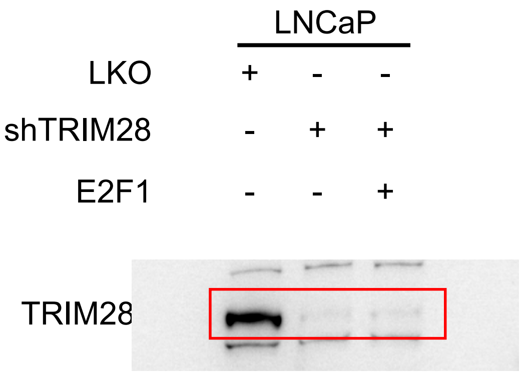

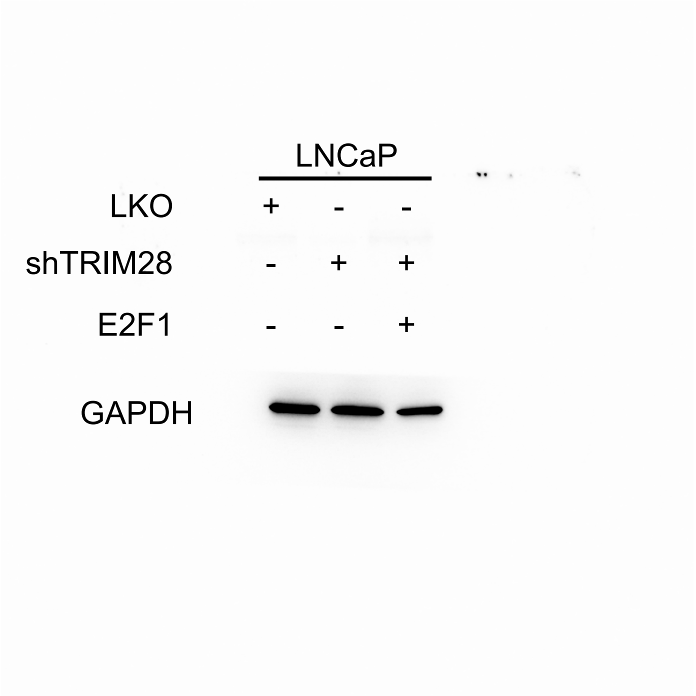

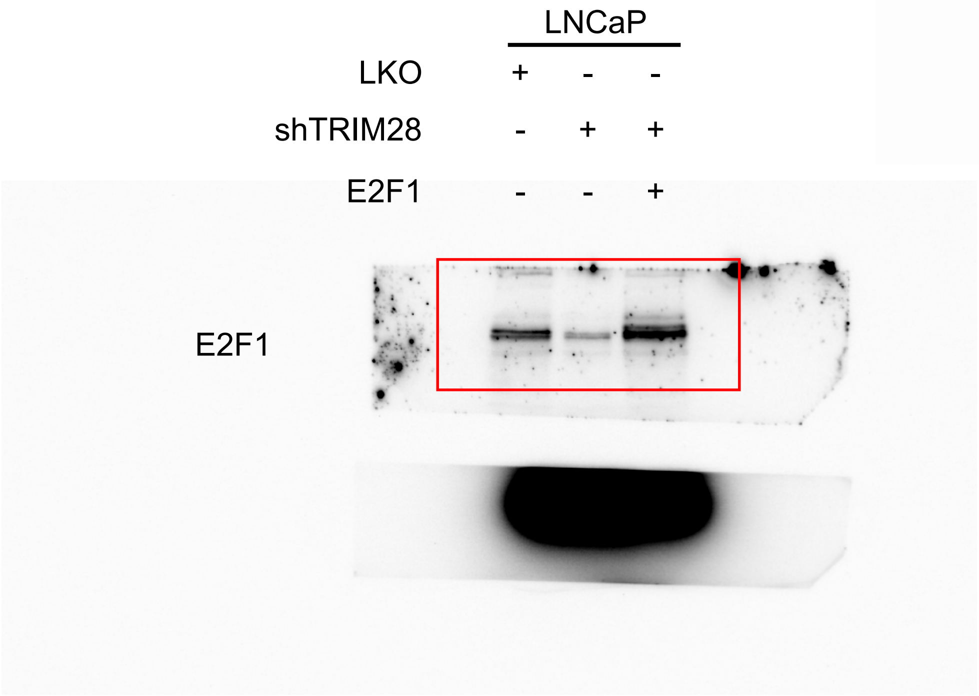

Figure S3A

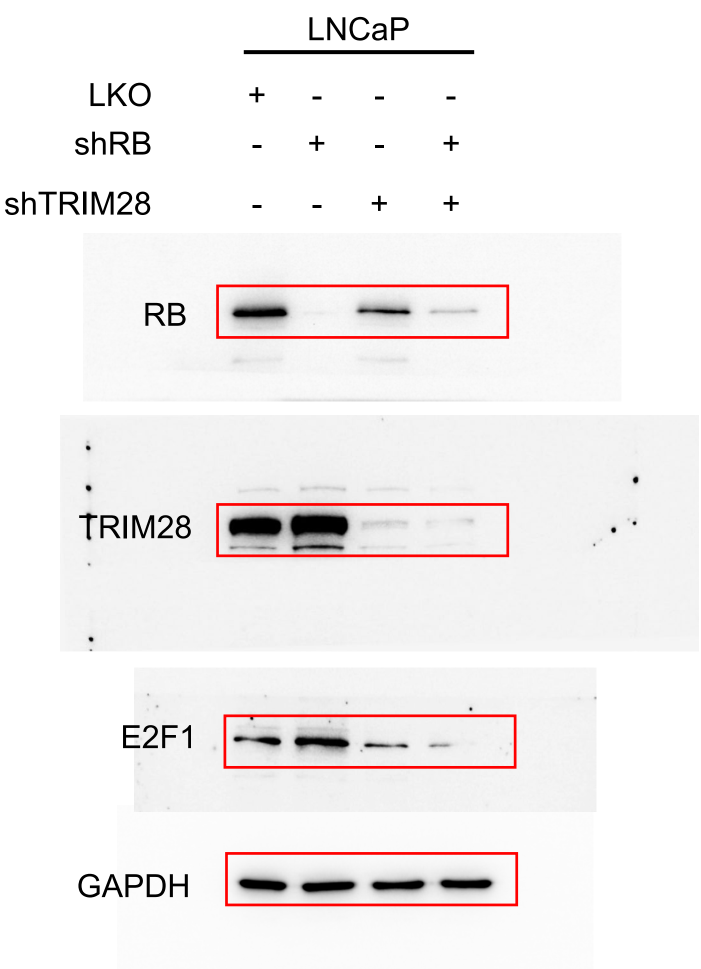

Full unedited blot for Figure 4E

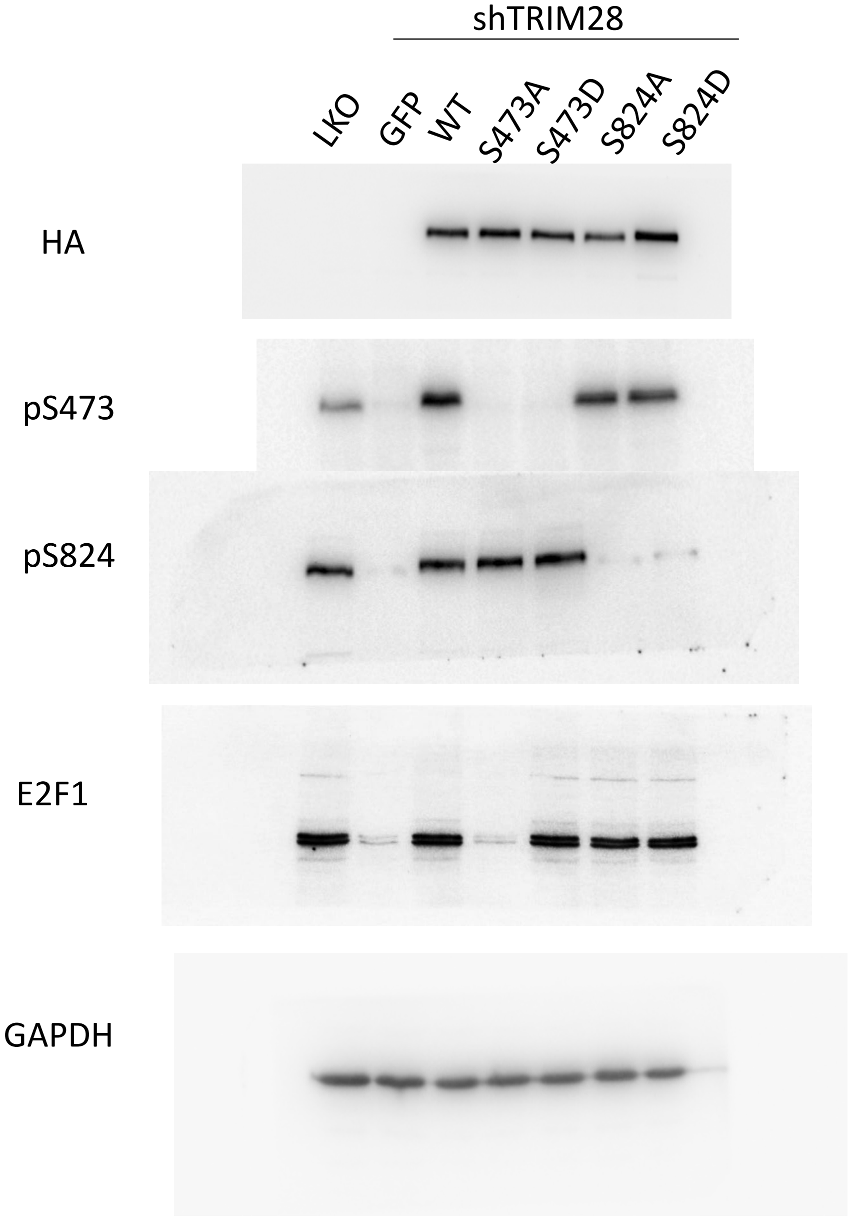

Figure 4G

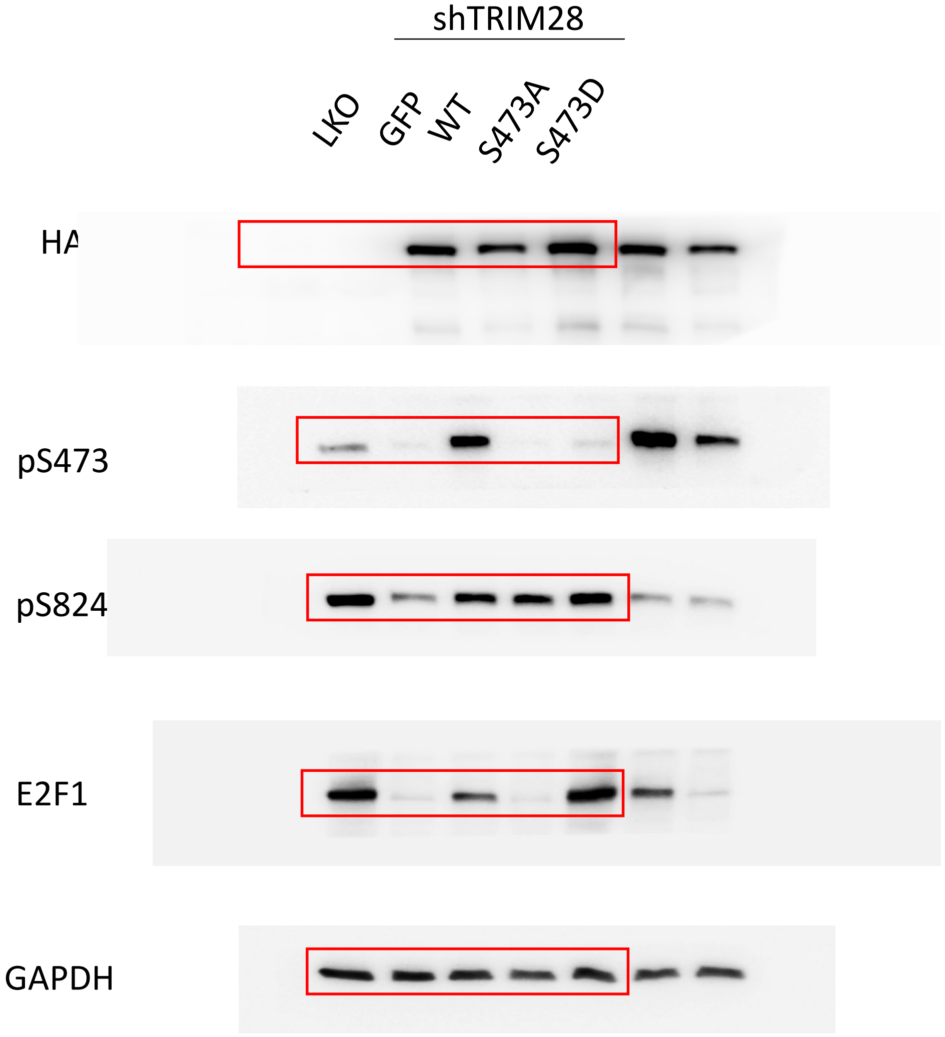

Full unedited blot for

Figure 5A

	Input		Flag IP	
Flag-GFP	+	-	+	-
Flag-TRIM28	-	+	-	+
MYC-RSK1	+	+	+	+

Flag

MYC

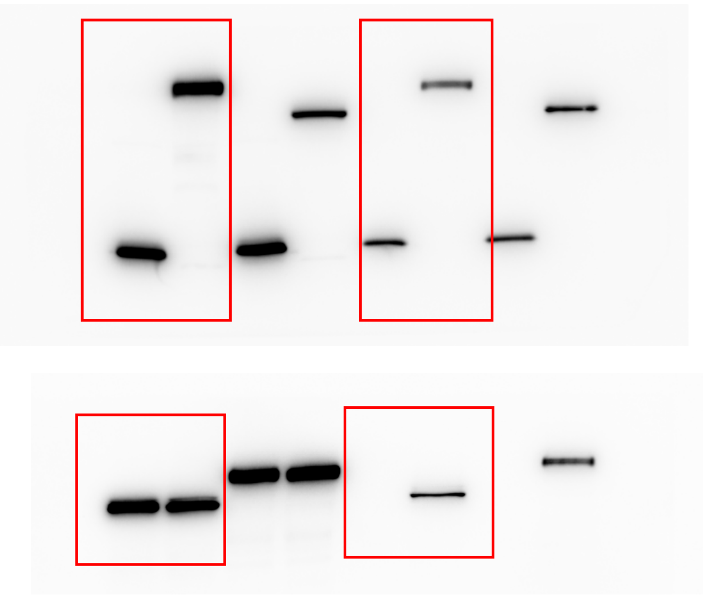

Figure 5B

	Input		Flag IP	
Flag-GFP	+	-	+	-
Flag-RSK1	-	+	-	+
MYC-TRIM28	+	+	+	+

Flag

MYC

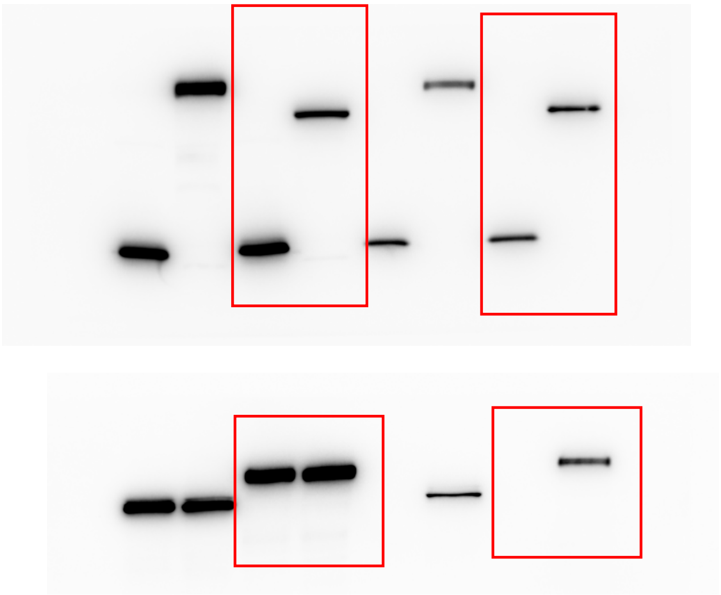

Figure 5D

GST-TRIM28	F1	F2	F1	F2
RSK1	-	-	+	+

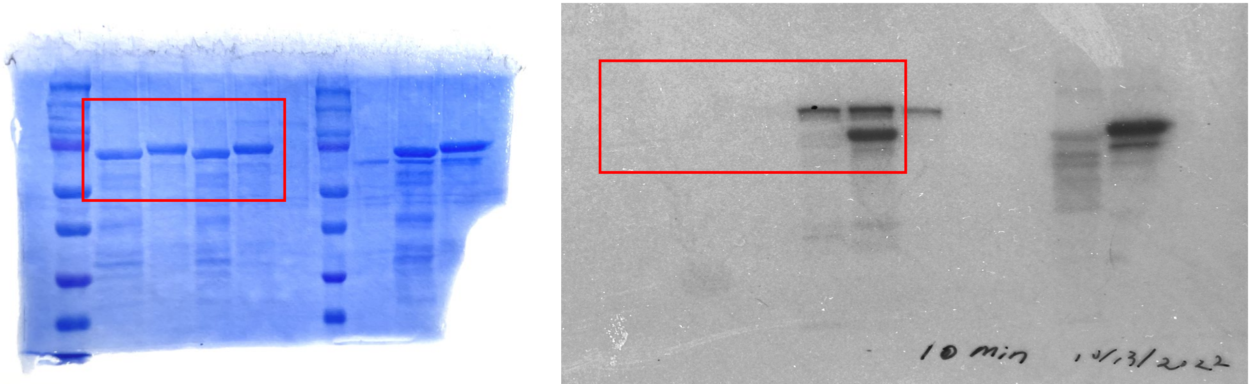

Figure 5E

GST-TRIM28	F1	F2	F1	F2
RSK1	+	+	+	+

pS473-TRIM28

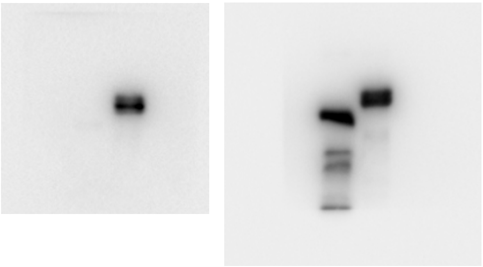

GST

Full unedited blot for

Figure 5F

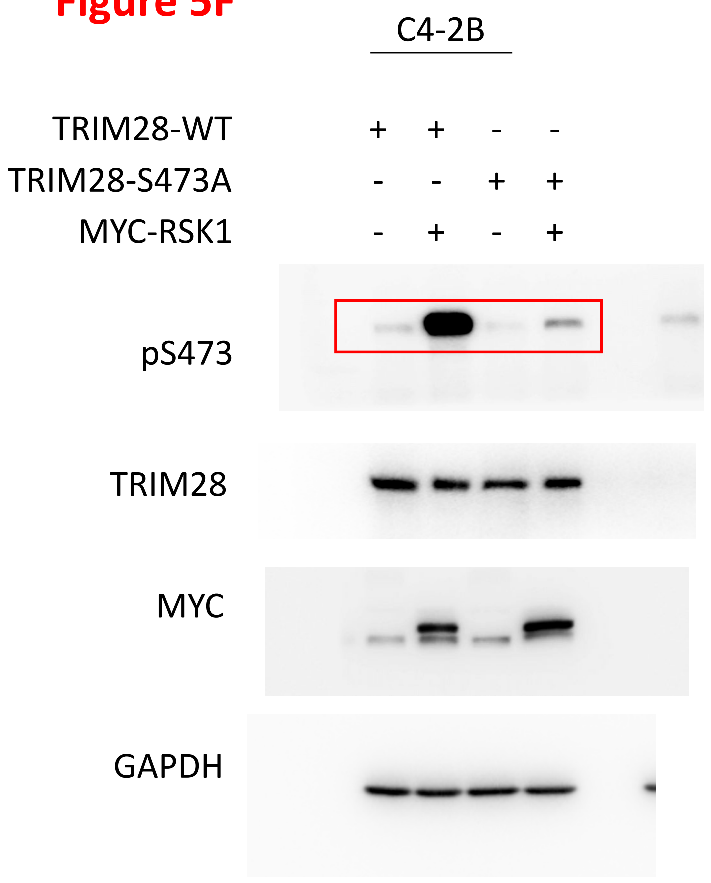

Figure 5G

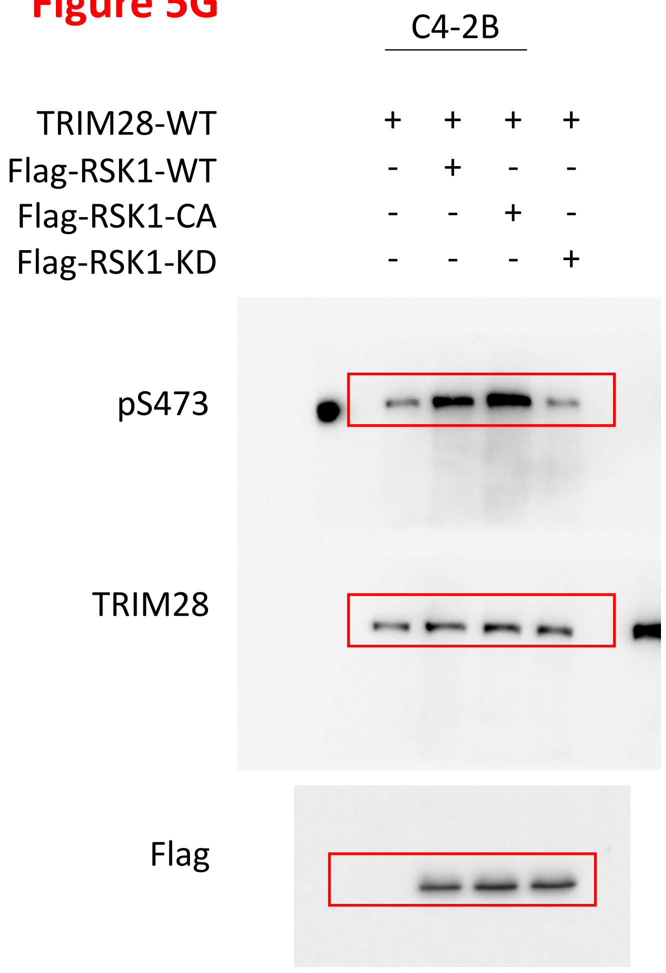

Full unedited blot for

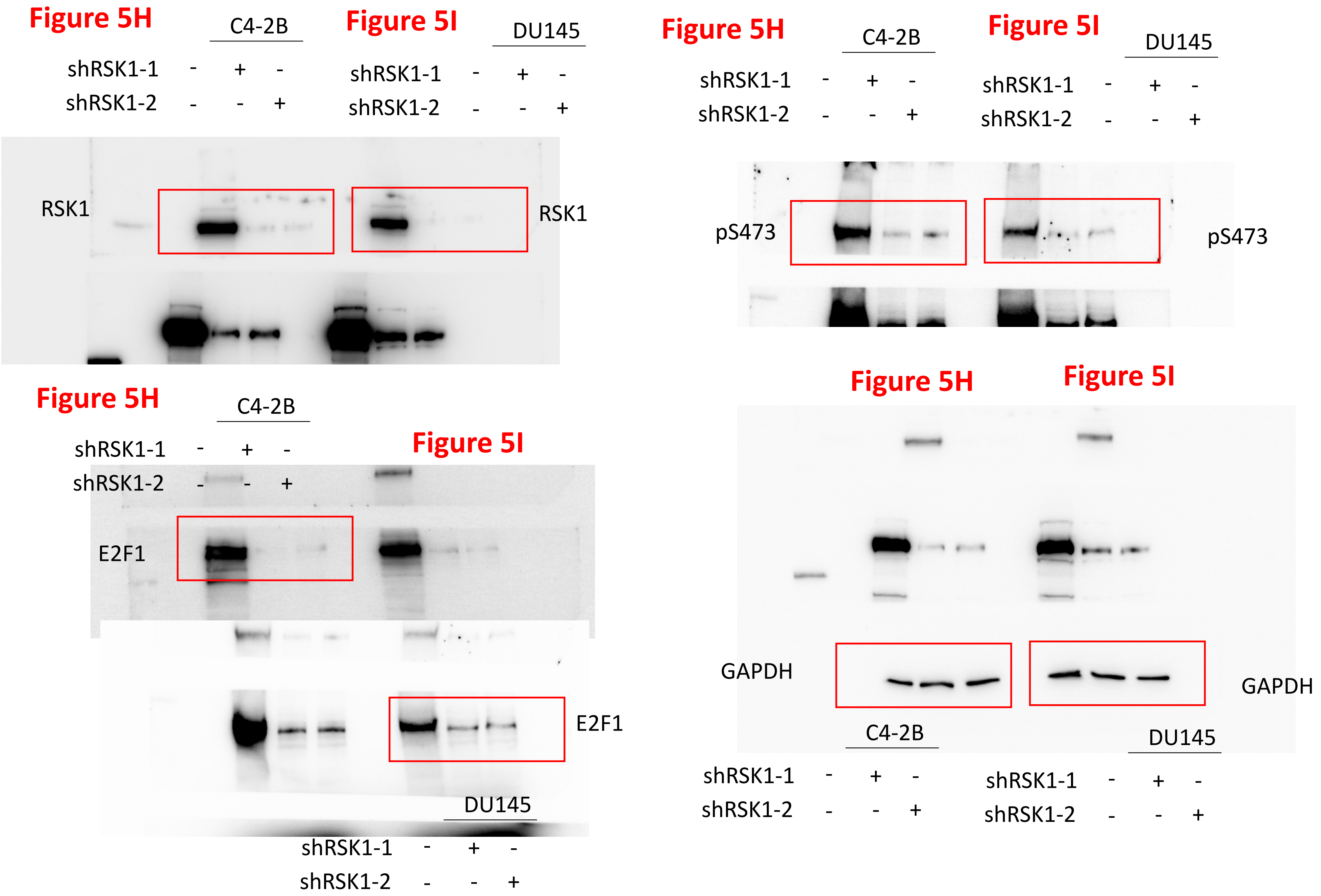

Full unedited blot for

Figure S5A

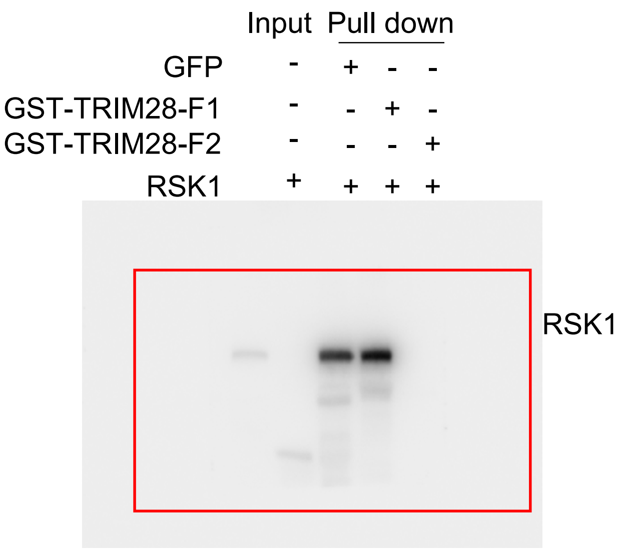

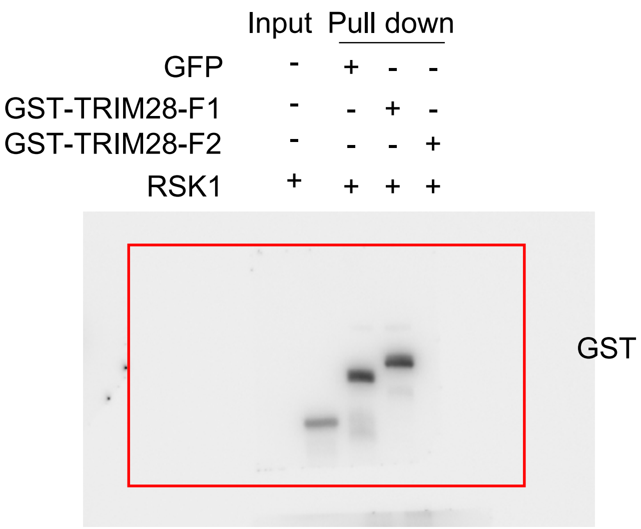

Full unedited blot for

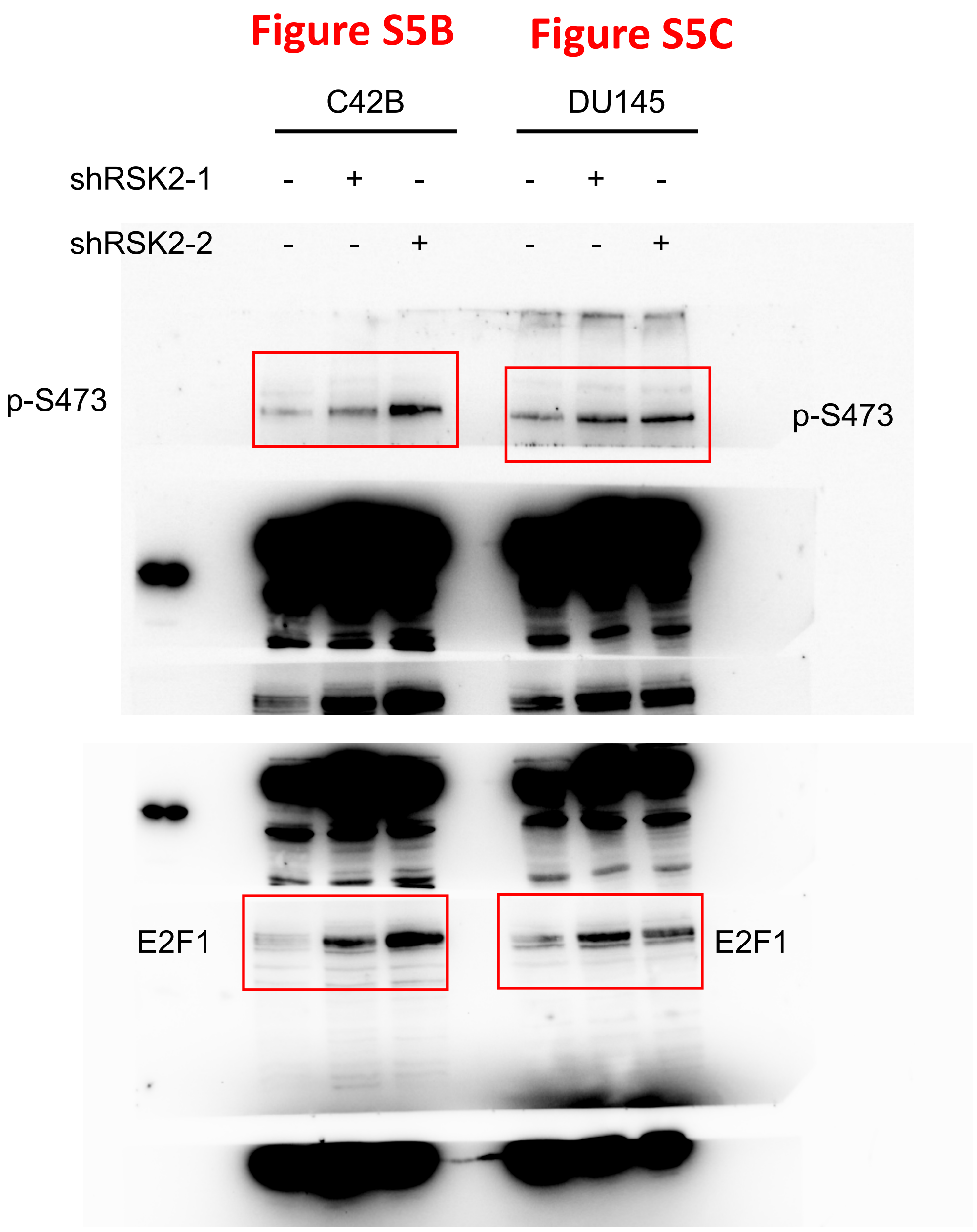

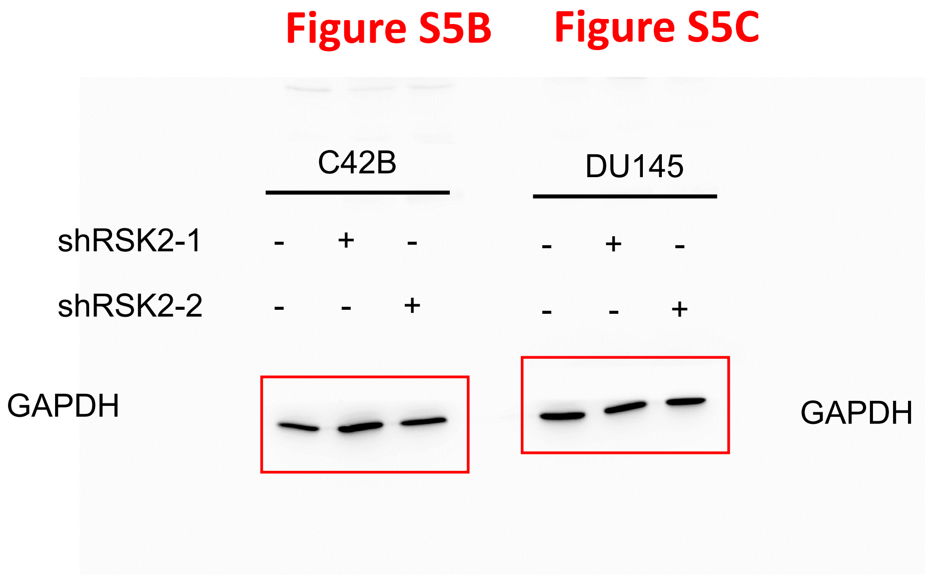

Full unedited blot for

Figure S5D C42B | Figure S5E DU145

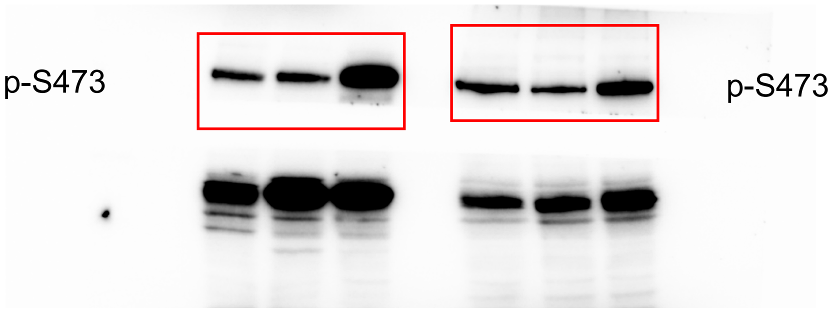

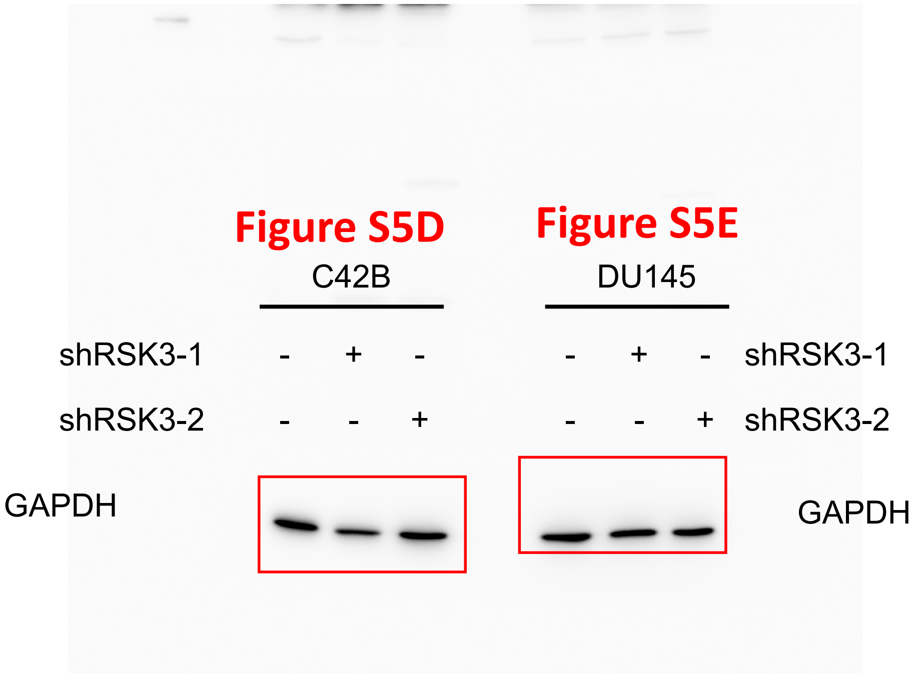

Figure S5D

C42B

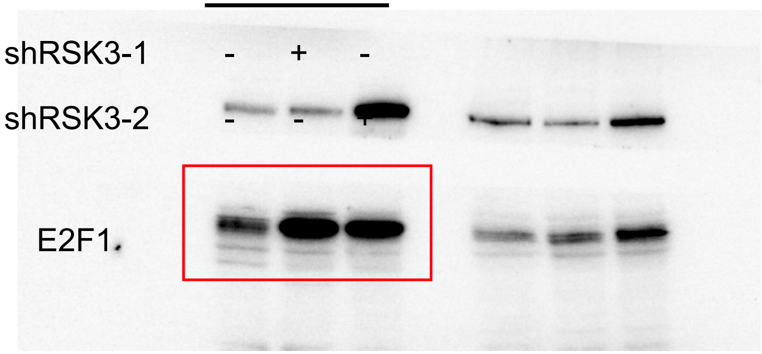

Figure S5E

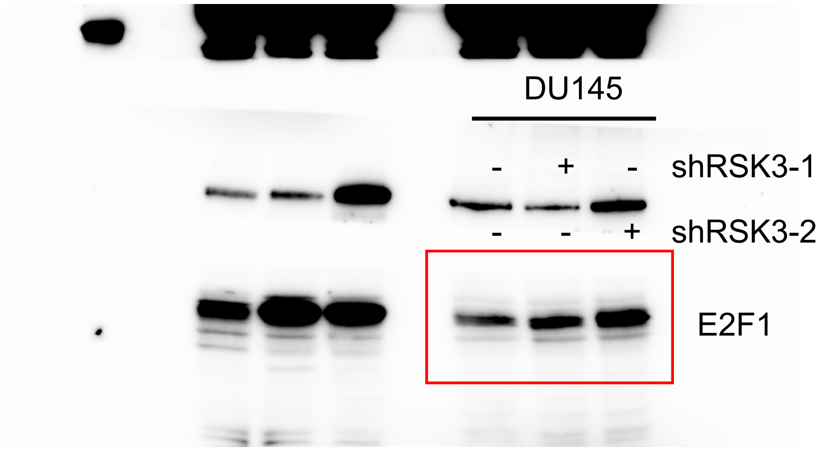

Full unedited blot for

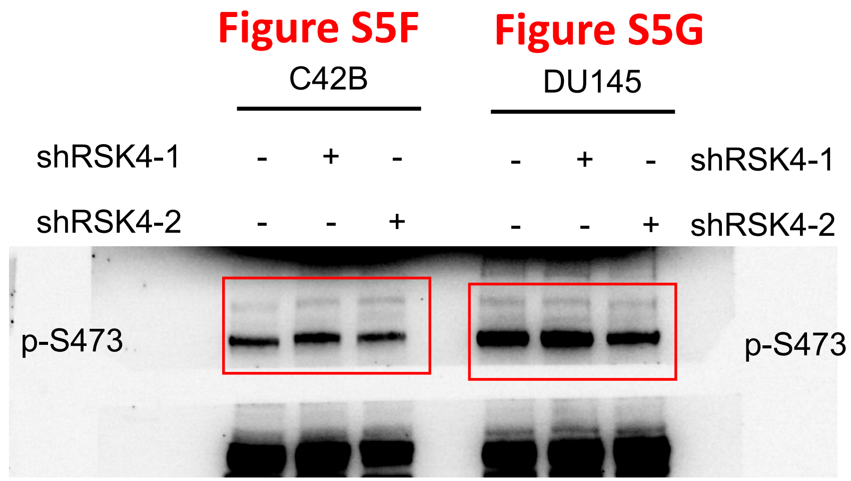

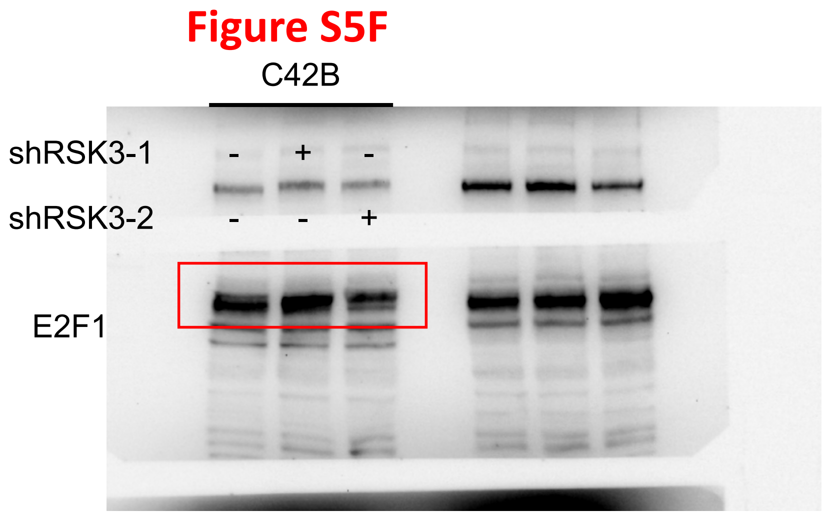

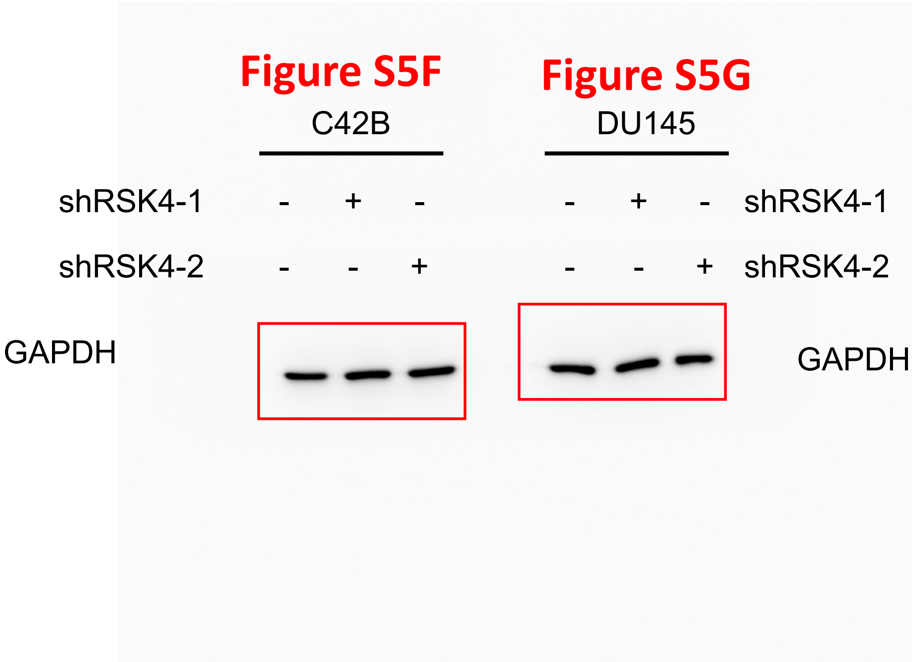

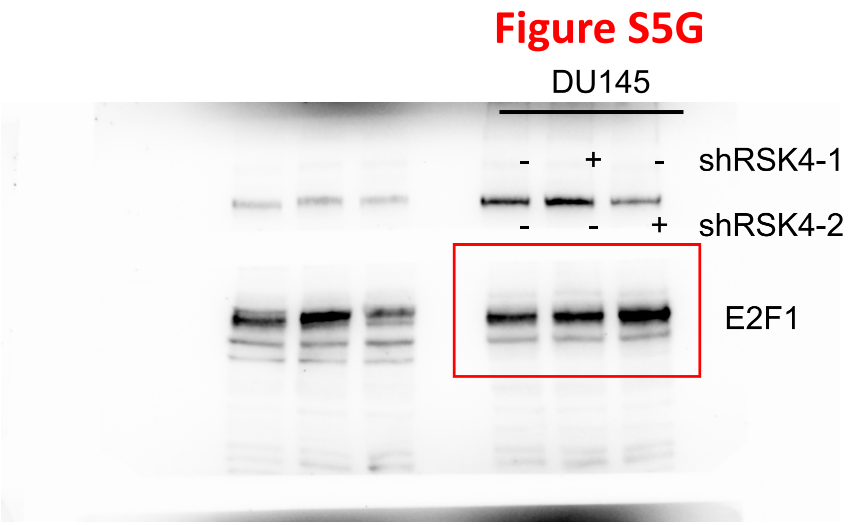

Full unedited blot for

Figure 6A Figure 6B Figure 6C

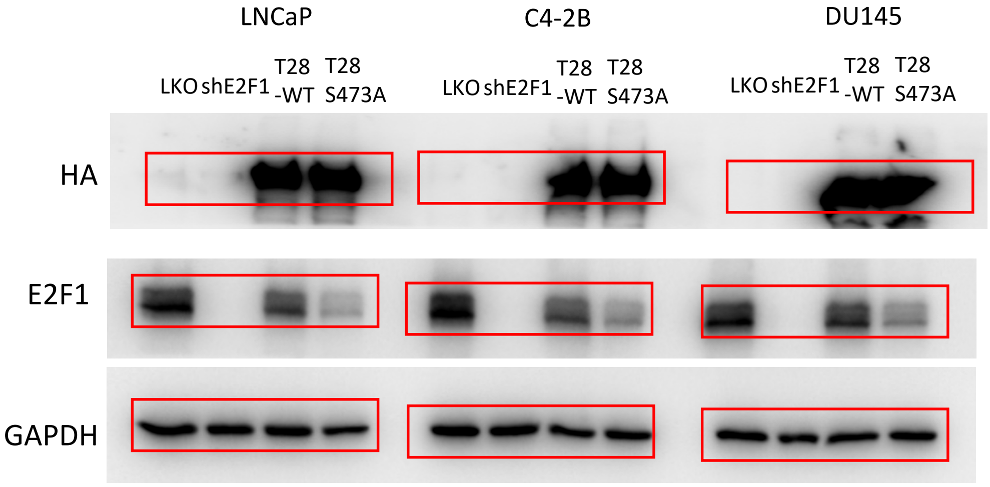

Figure 6L Figure 6M Figure 6N

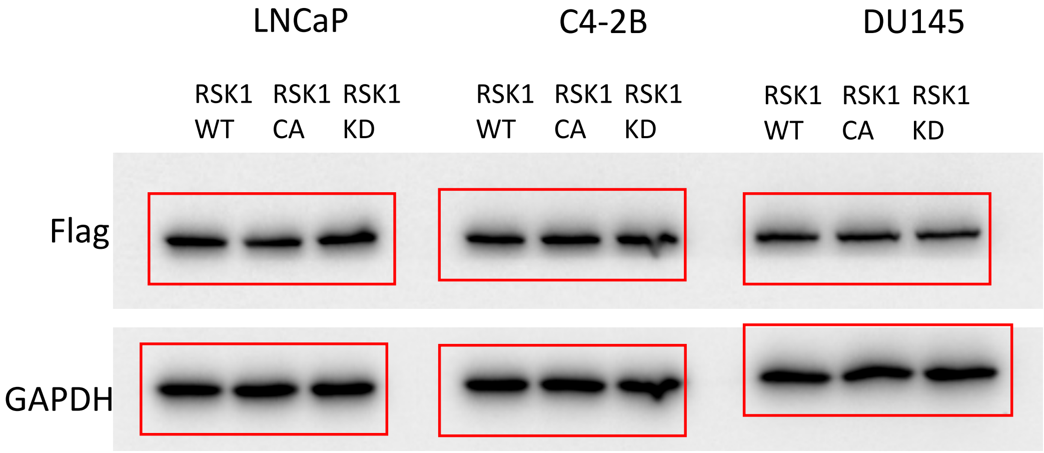

Full unedited blot for

Figure S6F   Figure S6H

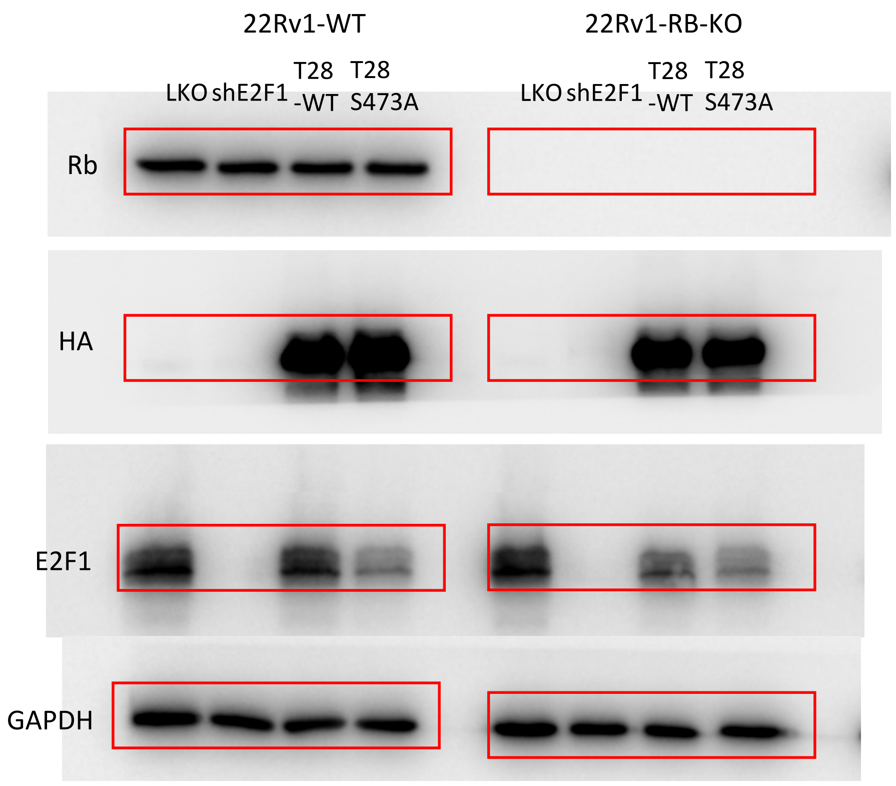

Figure S6J, S6M, S6P

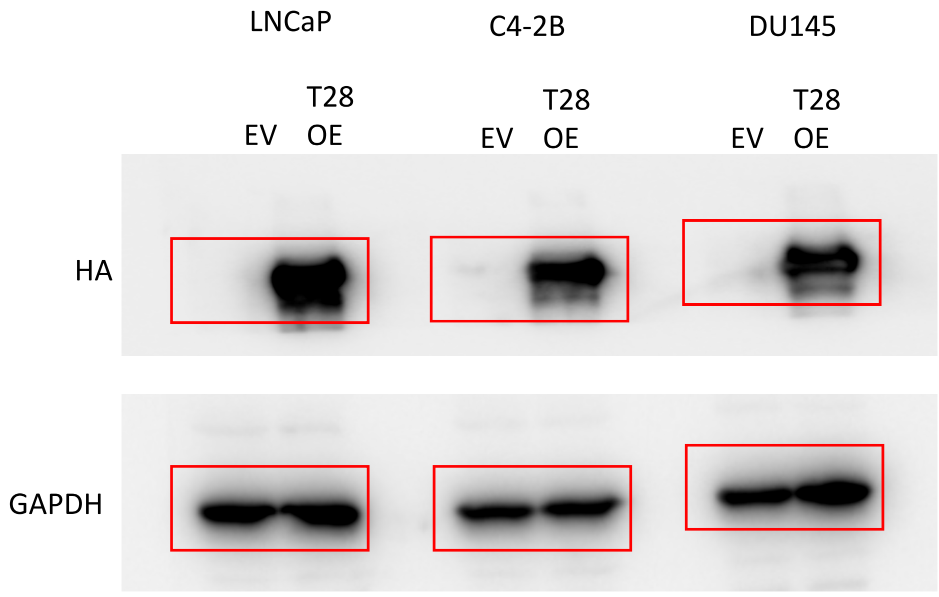

Figure S7E   Figure S7F   Figure S7G

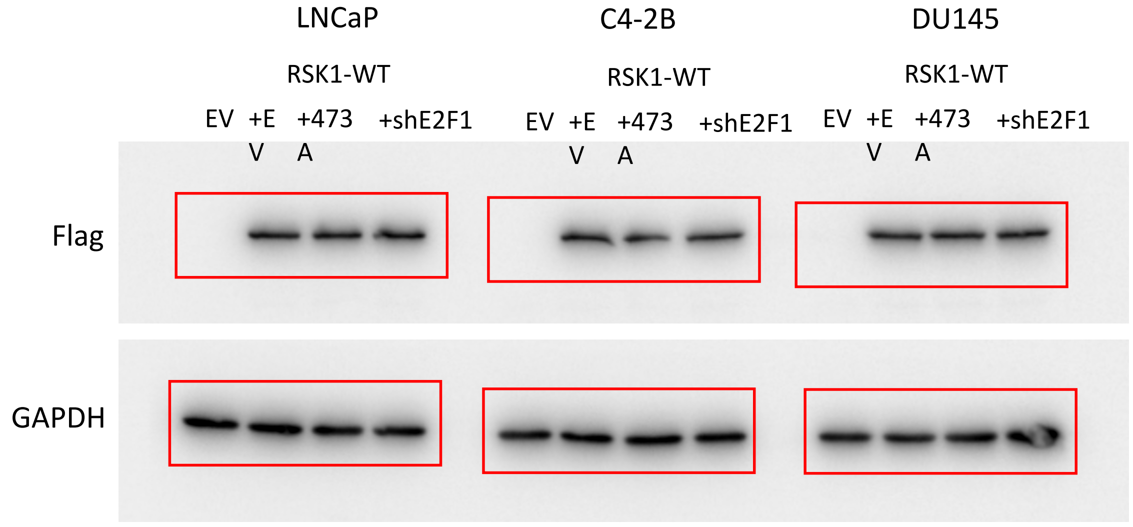

Full unedited blot for

Figure 7A

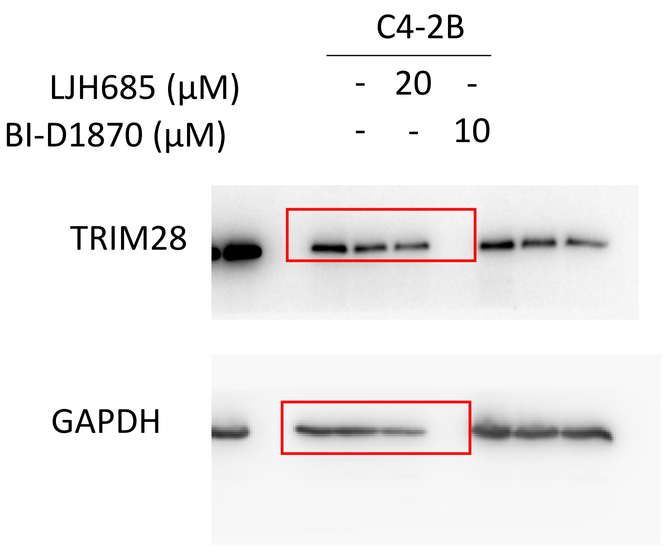

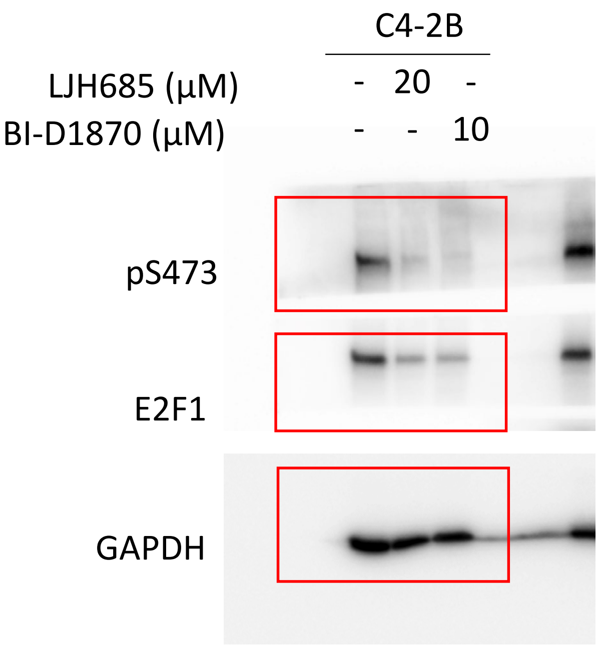

Full unedited blot for

Figure 7B

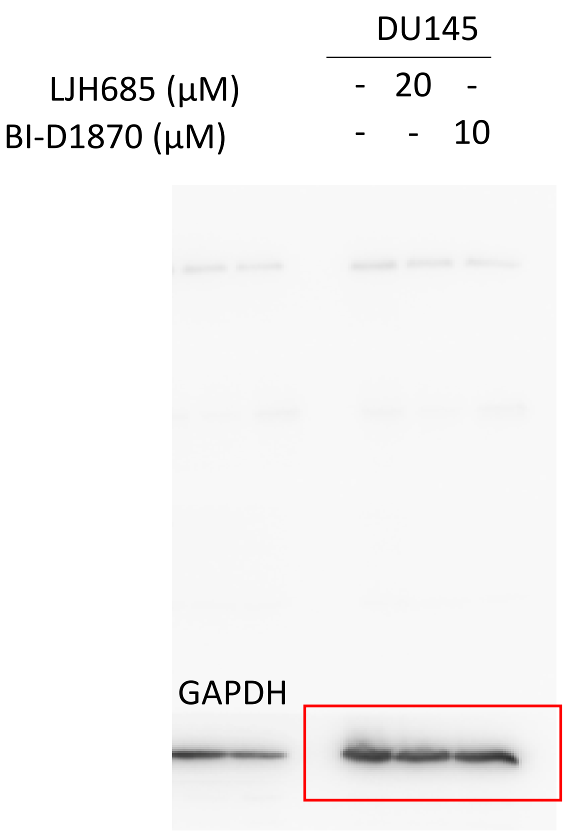

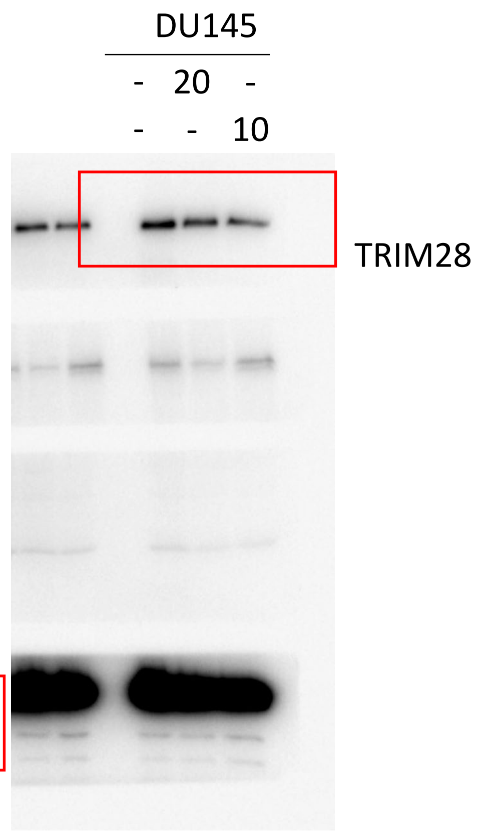

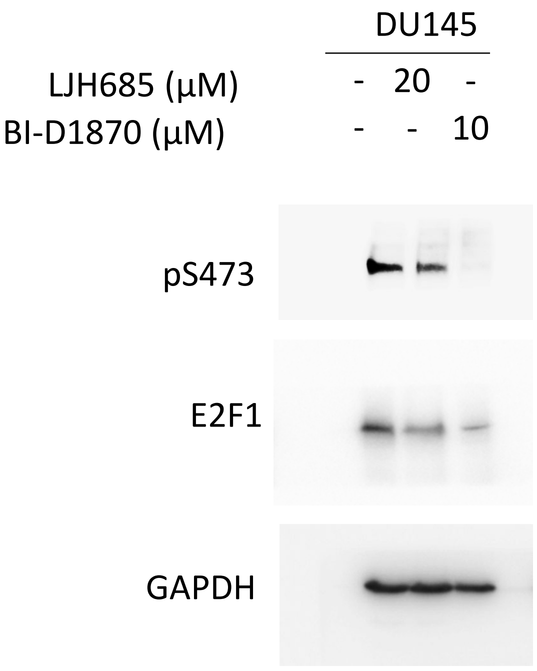

Full unedited blot for

Figure S8A

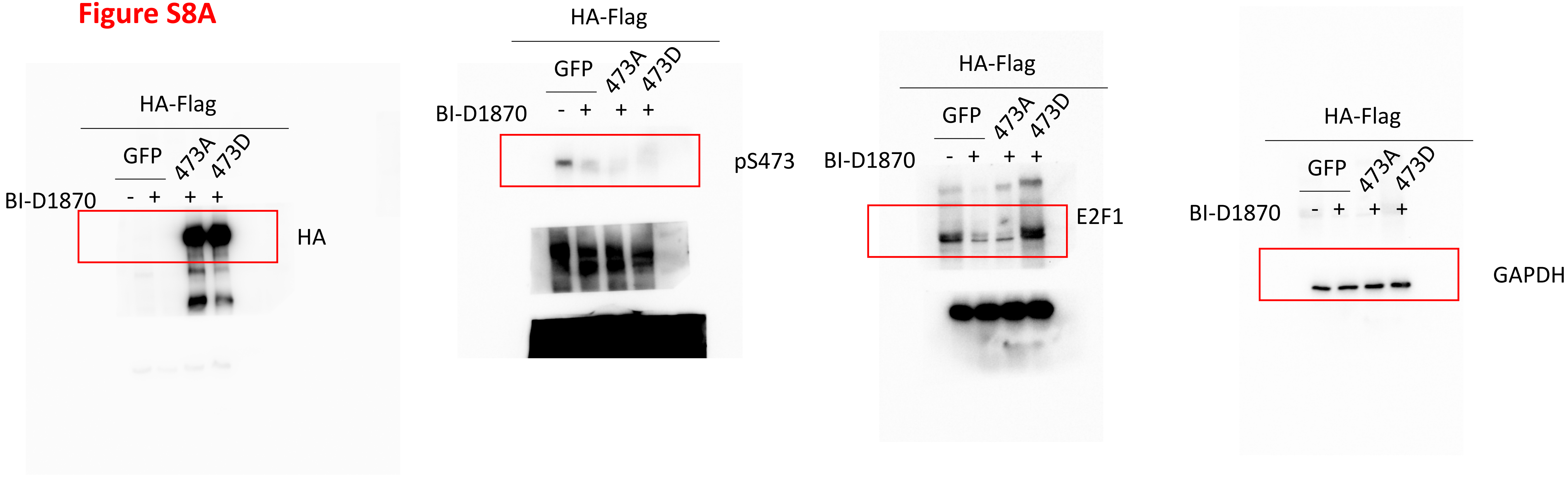

Full unedited blot for

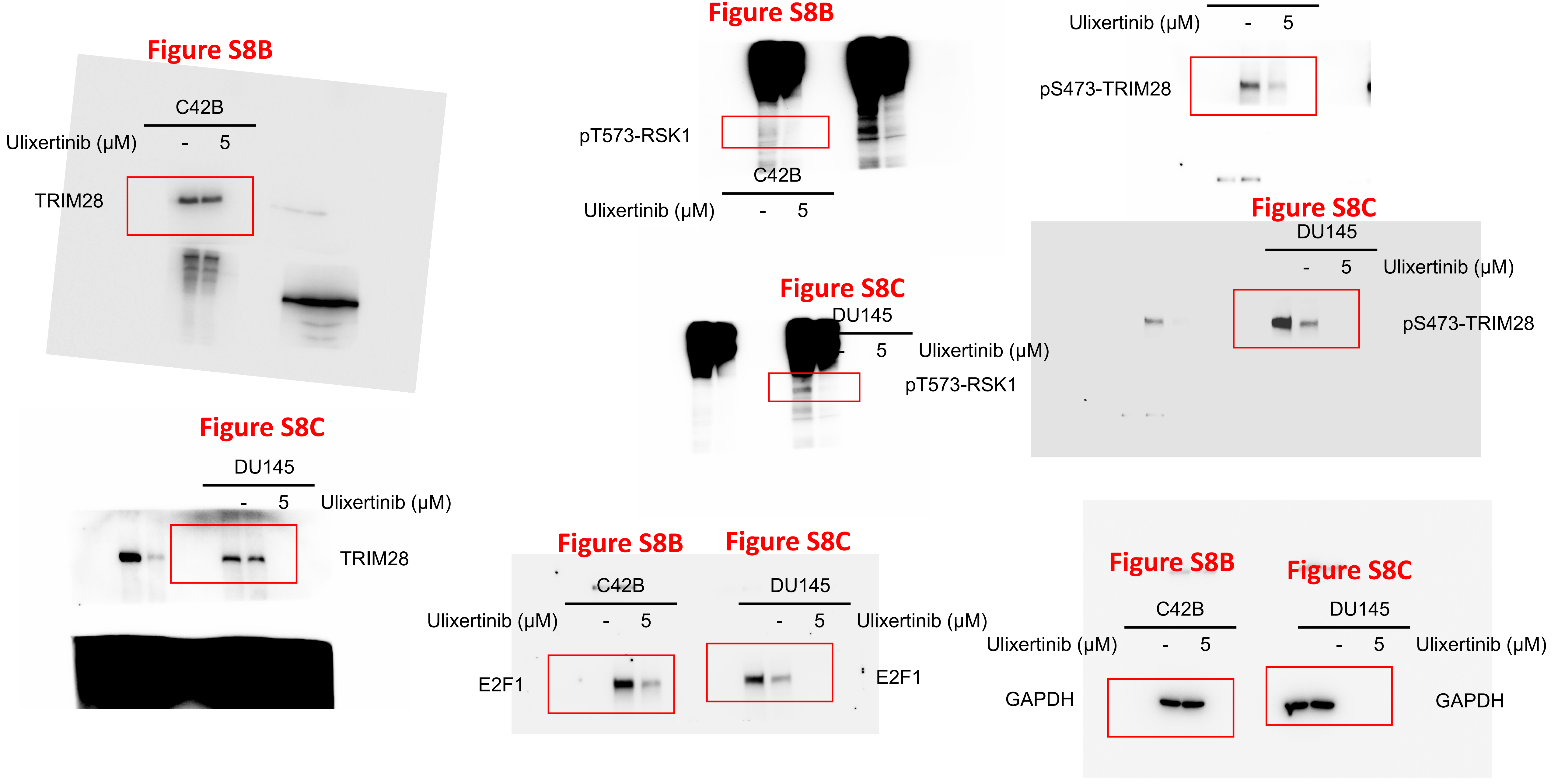

Full unedited blot for

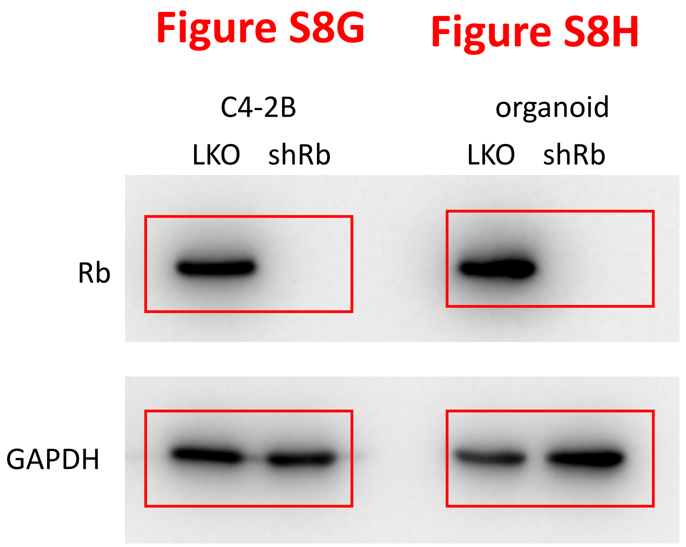

Supplement: Unedited blot and gel images [file jci-135-185119-s151.pdf]
